# Supplementary material for: Cyclin F drives proliferation through SCF-dependent degradation of the retinoblastoma-like tumor suppressor p130/RBL2
Source: eLife. 2021 Dec 1;10:e70691. doi: 10.7554/eLife.70691 (PMC8670743; doi:10.7554/eLife.70691)
Supplement: Source code 1. [file elife-70691-supp1.zip › split_violin_plot.rtf]

# plot measurement across all conditionsfeature =  "Int_pRB_over_RB_nuc"fig,ax = plt.subplots(1,1, figsize=(10,7))colors = ('red', 'black')ax = sns.violinplot(x="dox", y=feature, split=True, hue='p130', data=all_data_mod,                     palette=colors, inner=None, legend=False)ax.set_ylabel('phospho/total RB (RFU)', fontsize=22, labelpad=10)ax.set_xlabel('dox induction (d)', fontsize=22, labelpad=10)ax.get_legend().remove()plt.legend(fontsize=15)plt.xticks(fontsize=18)plt.yticks(fontsize=18)ax.xaxis.set_tick_params(width=2)ax.yaxis.set_tick_params(width=2)for axis in ['top','bottom','left','right']:    ax.spines[axis].set_linewidth(2)plt.show()
