# Supplementary figures and images for: Cyclin F drives proliferation through SCF-dependent degradation of the retinoblastoma-like tumor suppressor p130/RBL2
Source: eLife. 2021 Dec 1;10:e70691. doi: 10.7554/eLife.70691 (PMC8670743; doi:10.7554/eLife.70691)

# Enrico\_Fig7 S2\_Source Data 1 For Fig7S2A

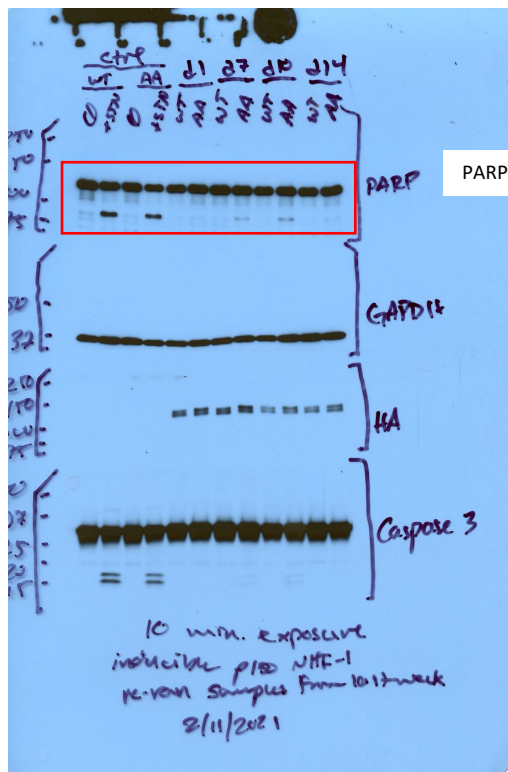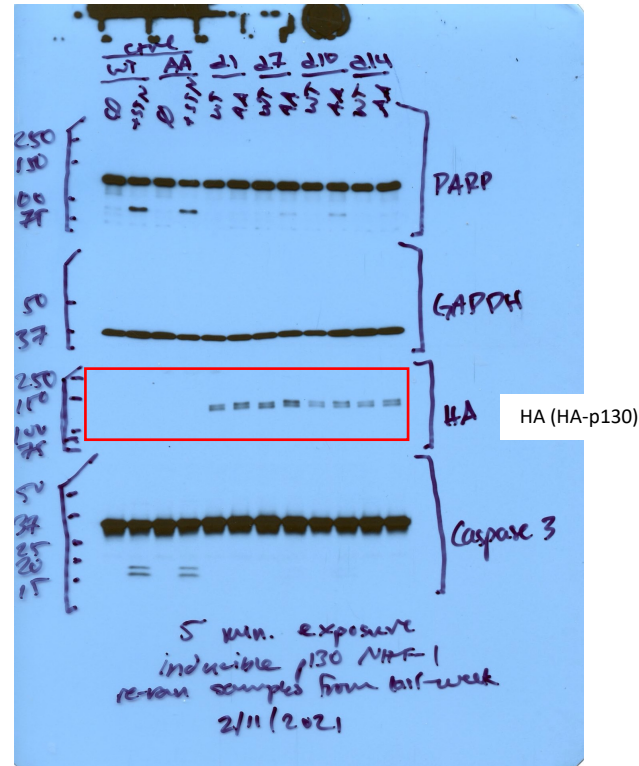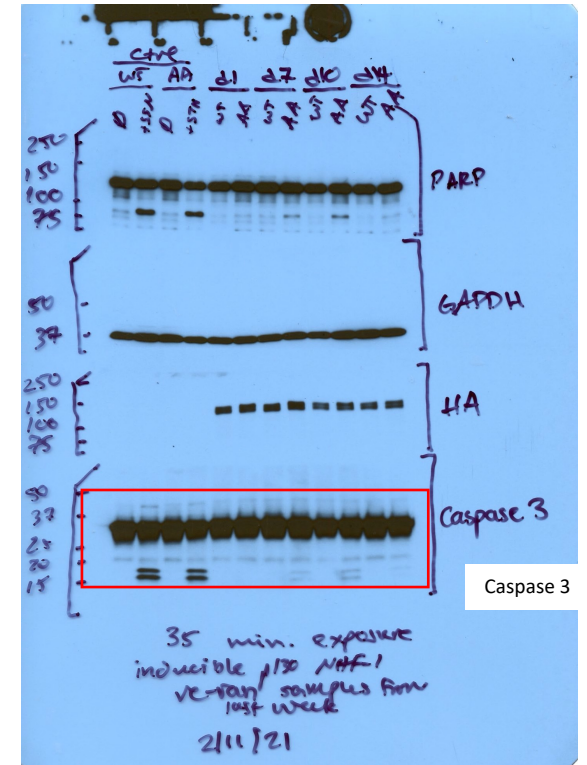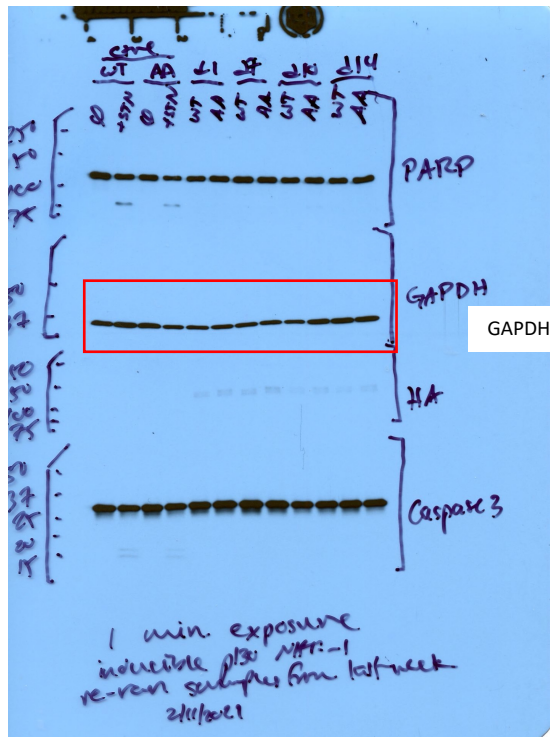

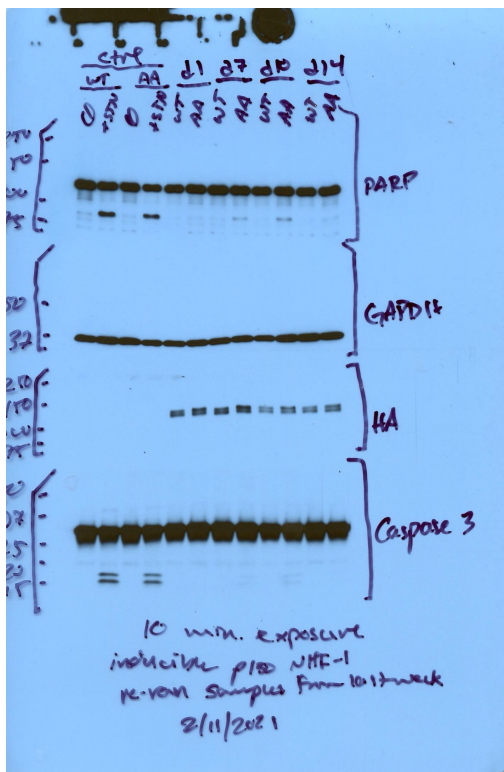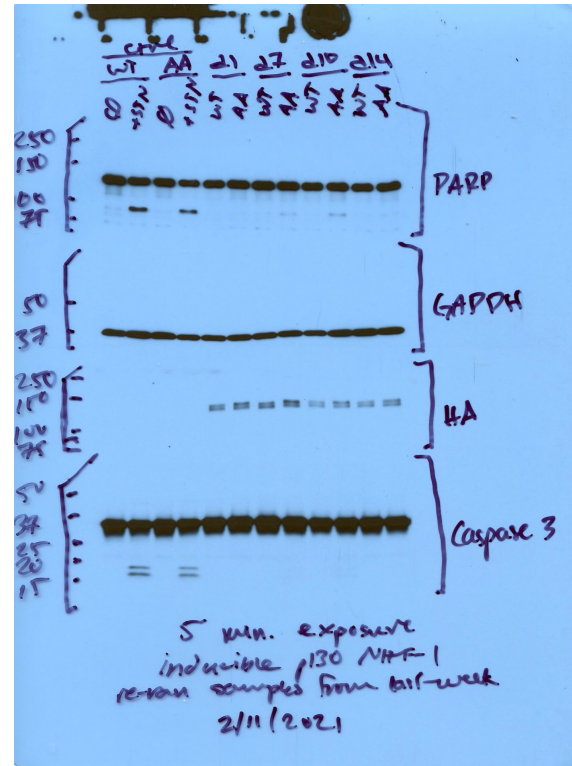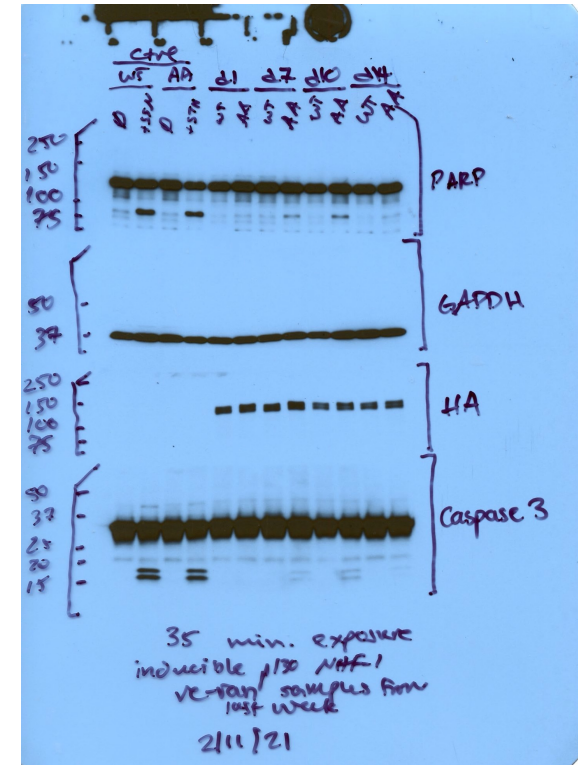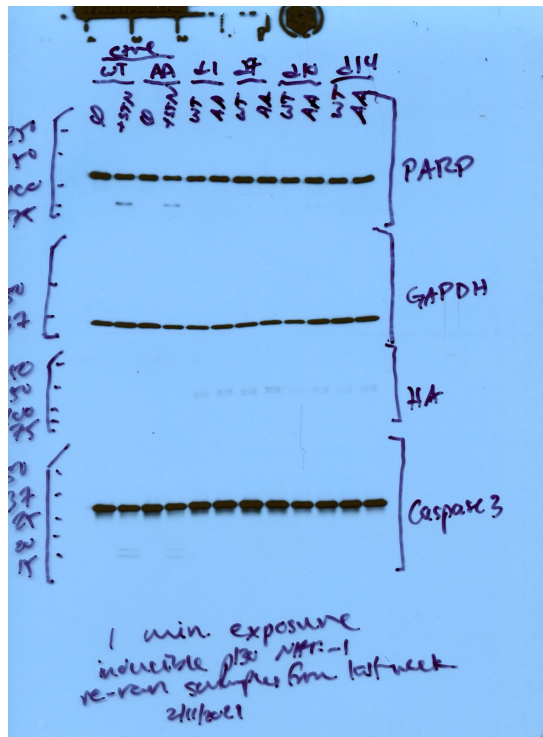

Supplement: Source data 1. — This source data file includes all uncropped blots used to generate data for the main figures and figure supplements. Additionally, copies of the uncropped images are shown a second time where blot strips shown in figures are highlighted with a red square and the protein that was blotted for is noted. [file elife-70691-supp2.zip › Source Data 1/Enrico-Fig7S2-source-data.pdf]

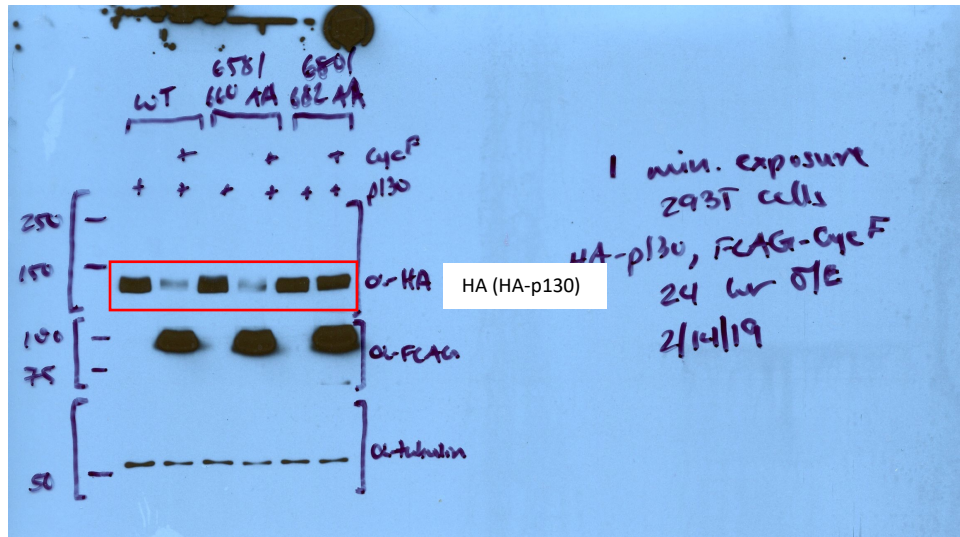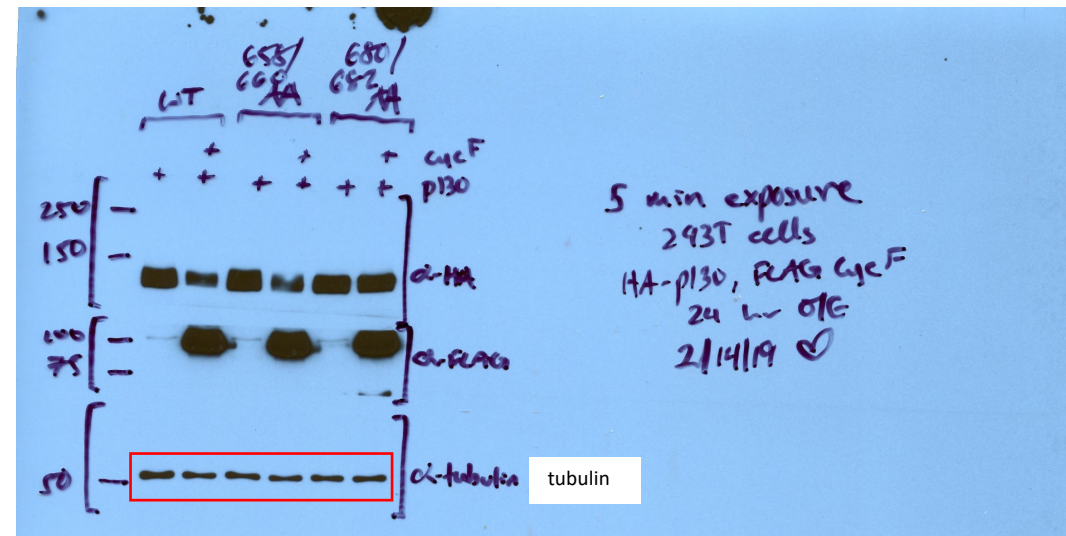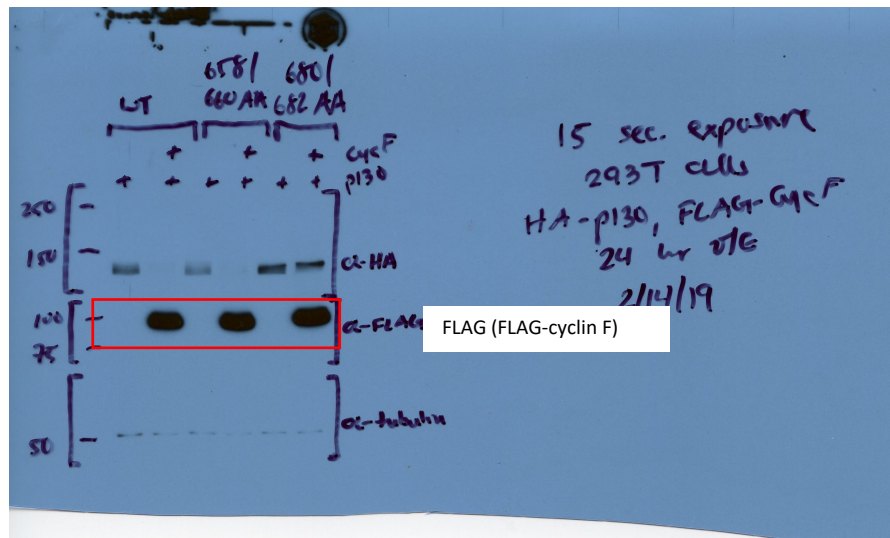

Left side:

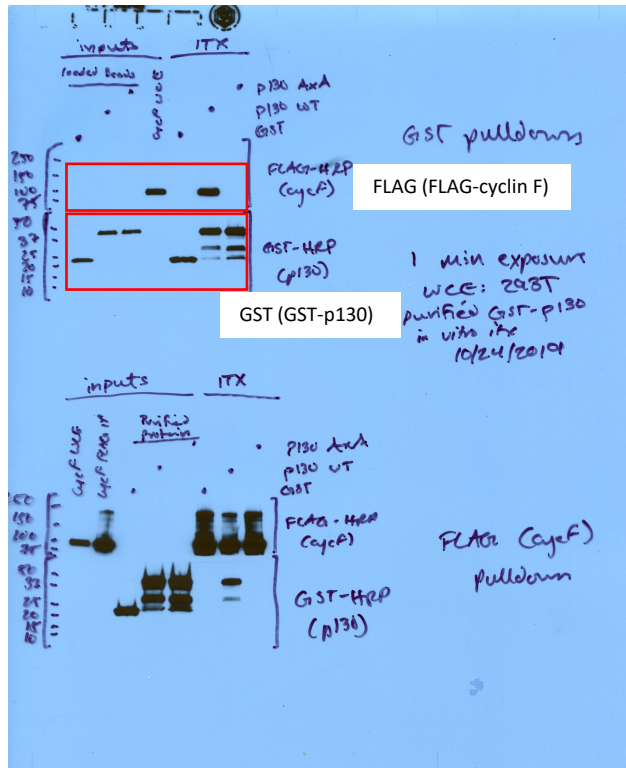

Right side:

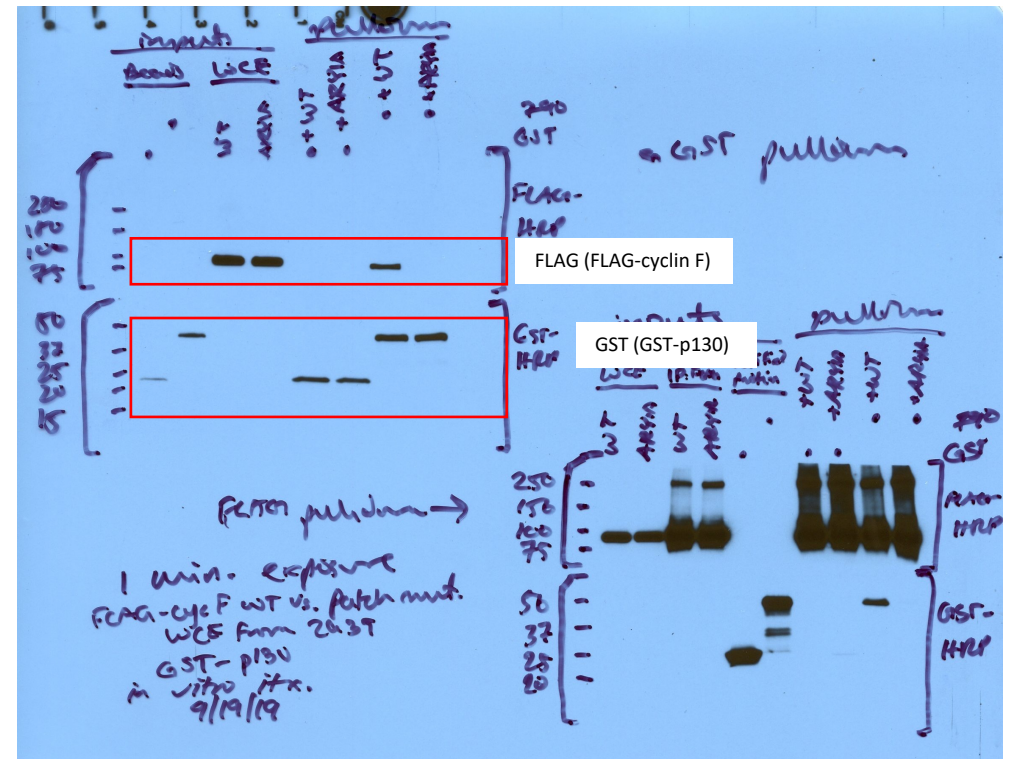

**Right side:**

**Left side:**

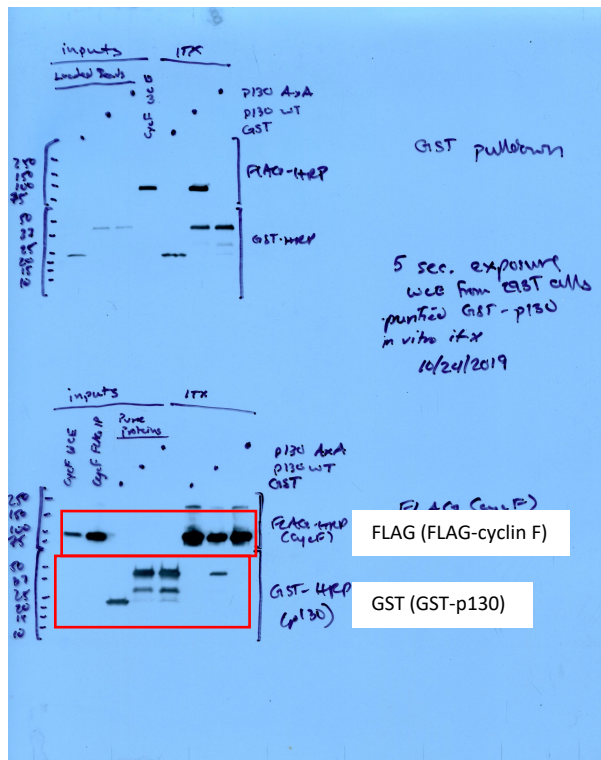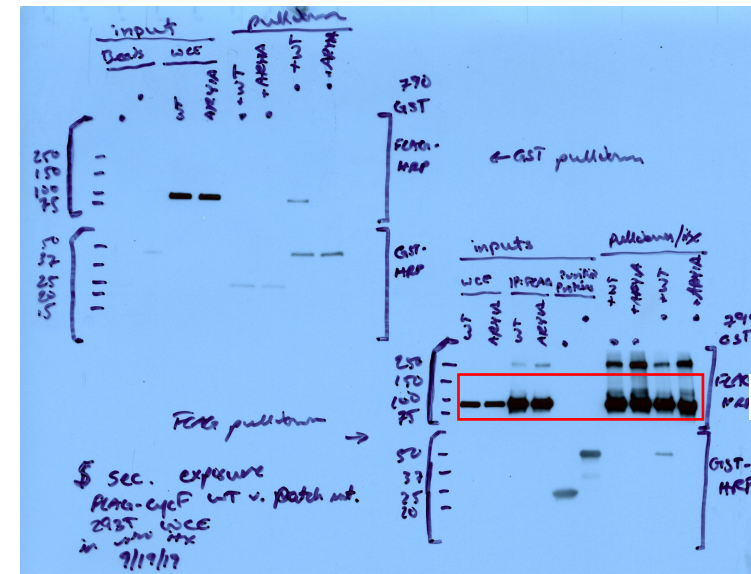

FLAG (FLAG-cyclin F)

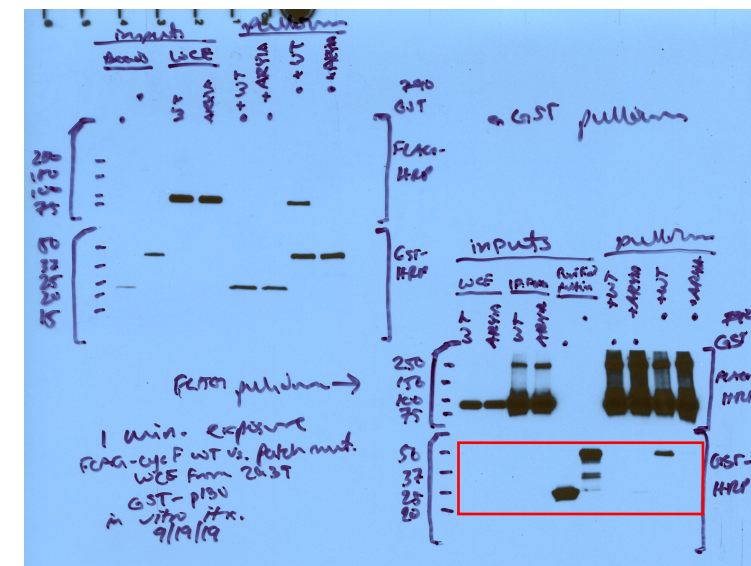

GST (GST-p130)

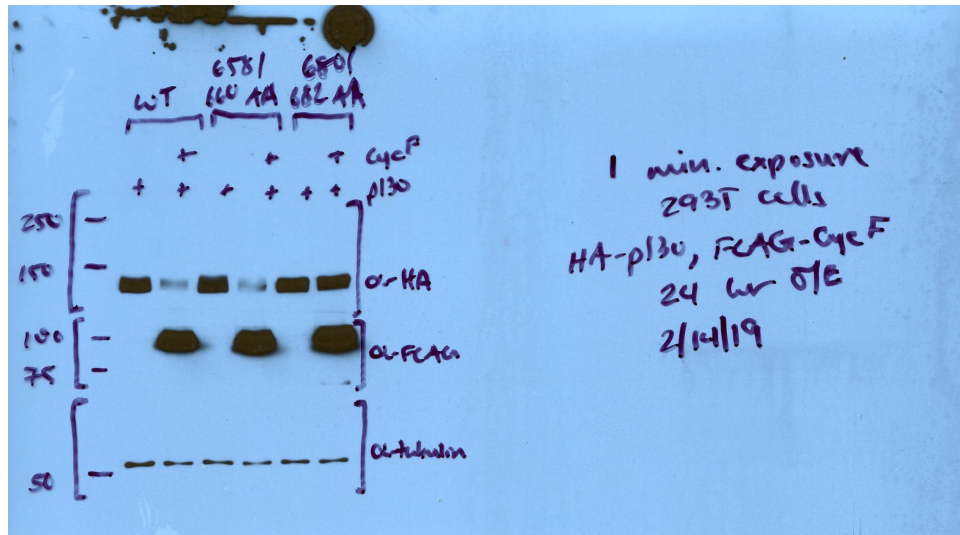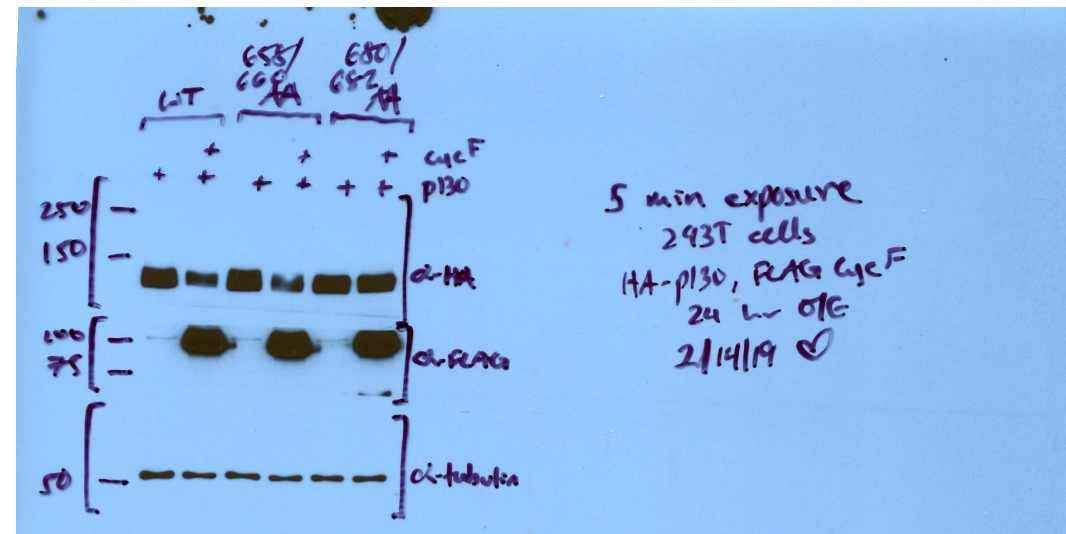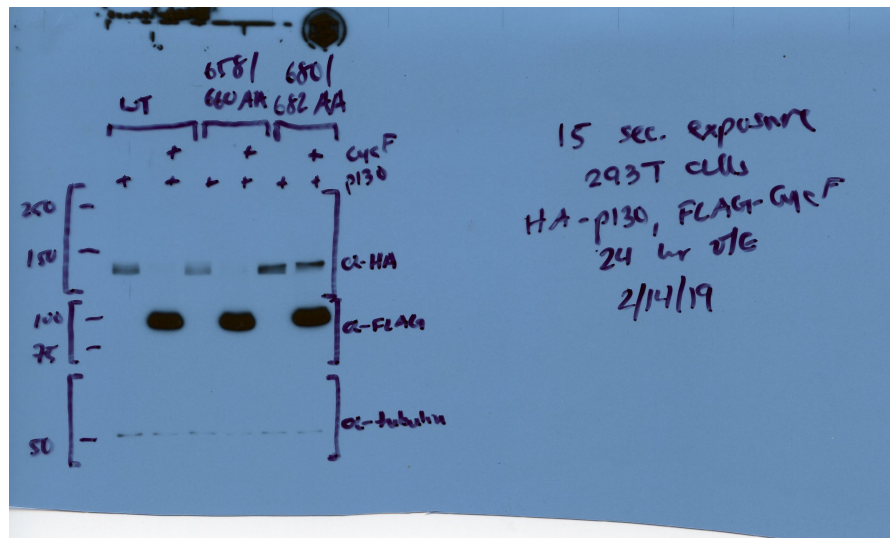

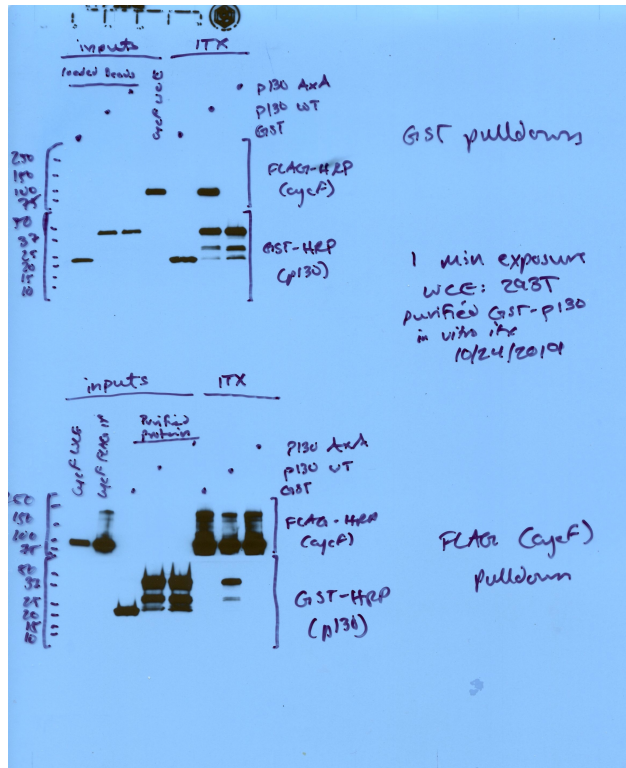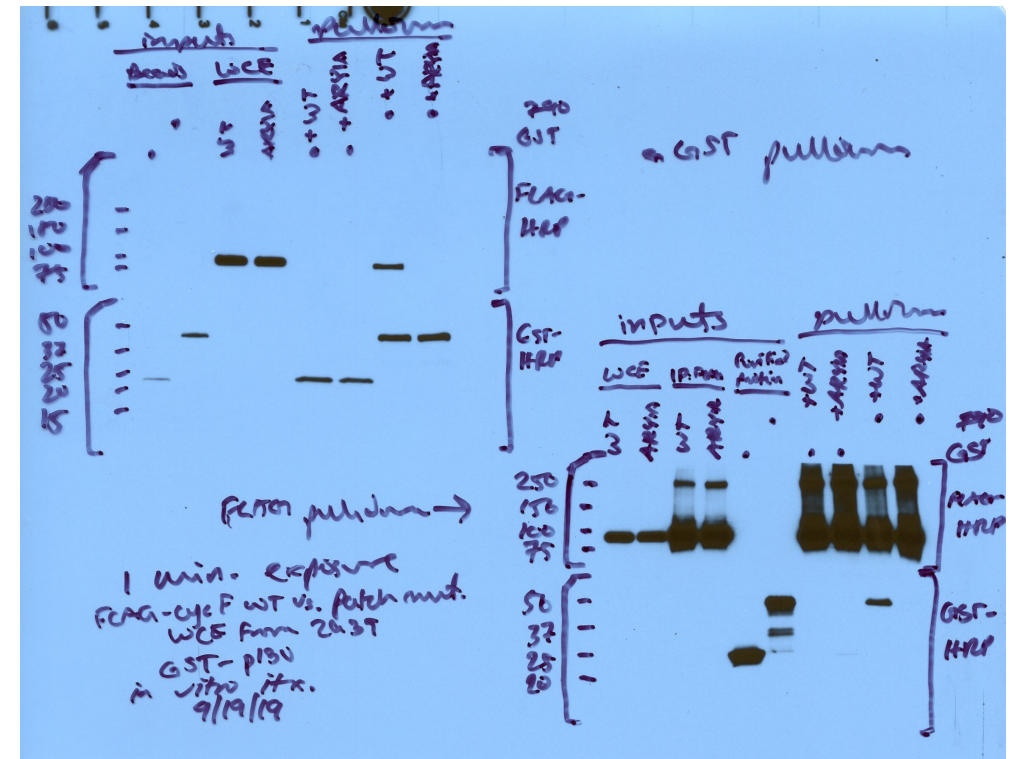

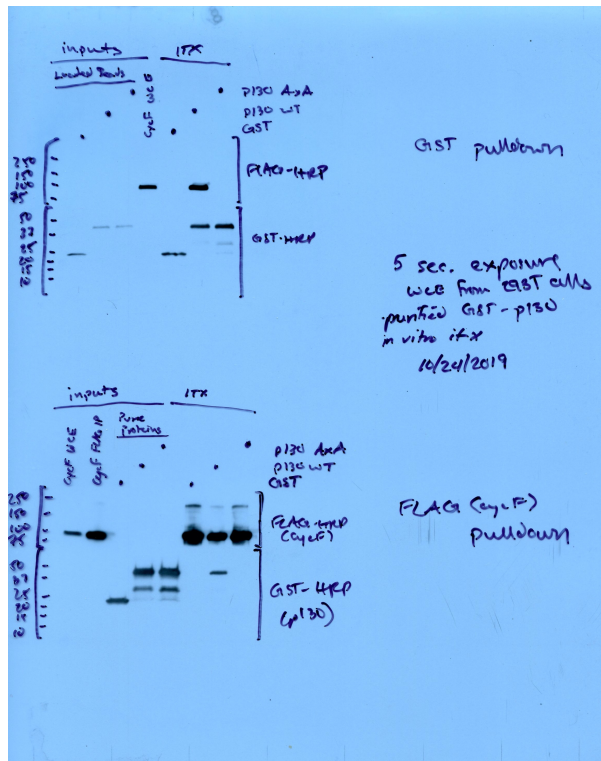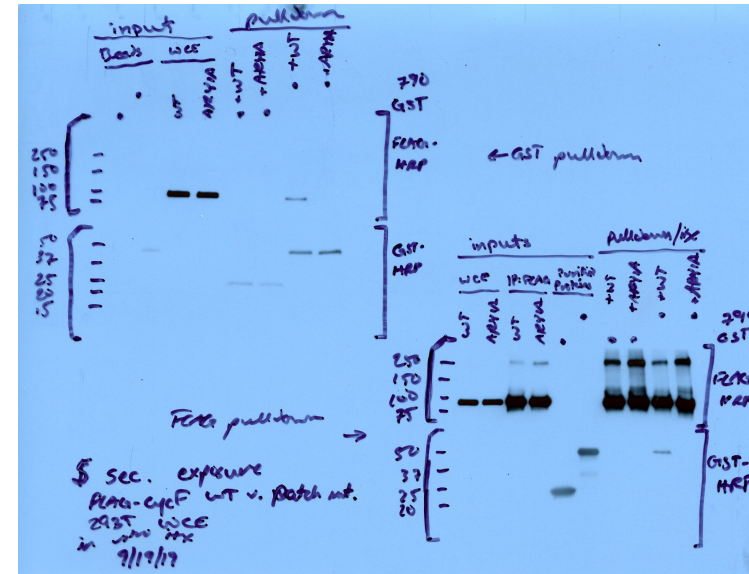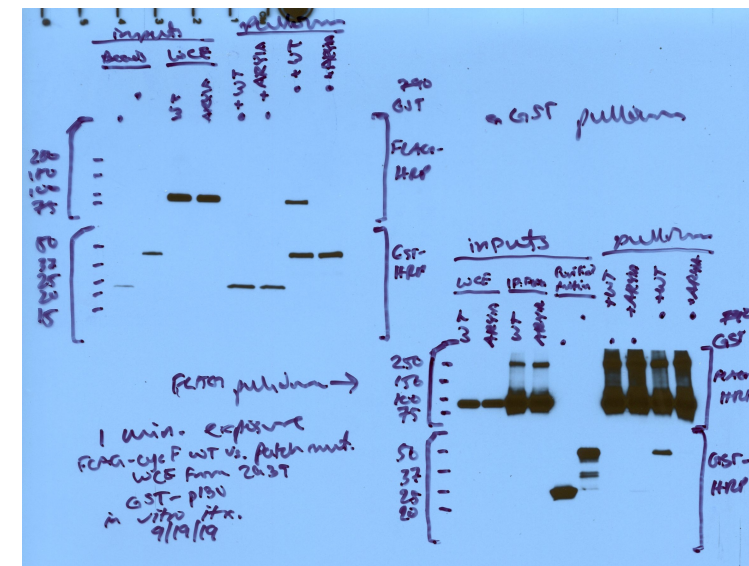

Supplement: Source data 1. — This source data file includes all uncropped blots used to generate data for the main figures and figure supplements. Additionally, copies of the uncropped images are shown a second time where blot strips shown in figures are highlighted with a red square and the protein that was blotted for is noted. [file elife-70691-supp2.zip › Source Data 1/Enrico-Fig4-source-data.pdf]

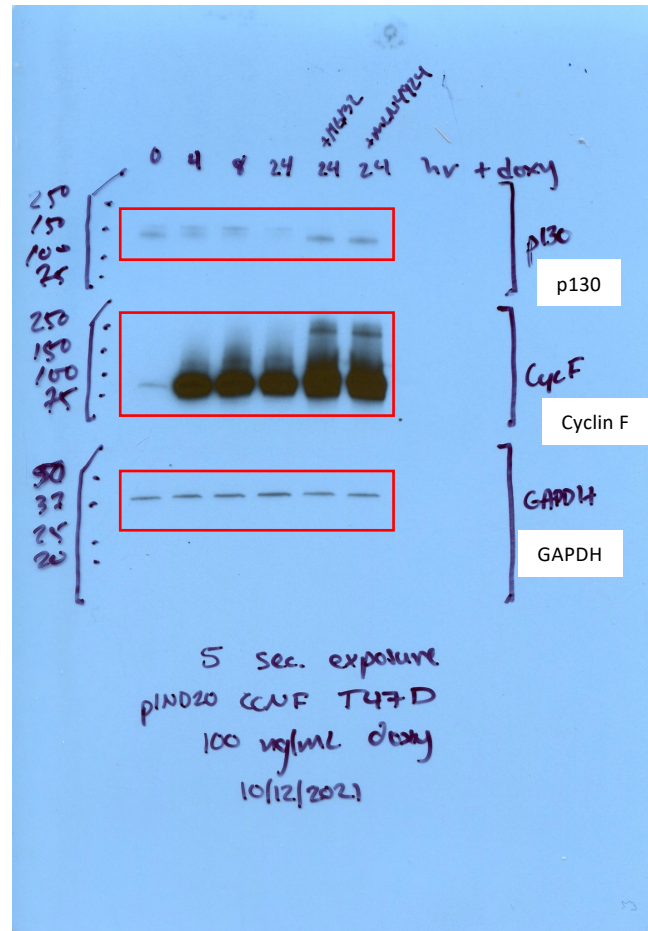

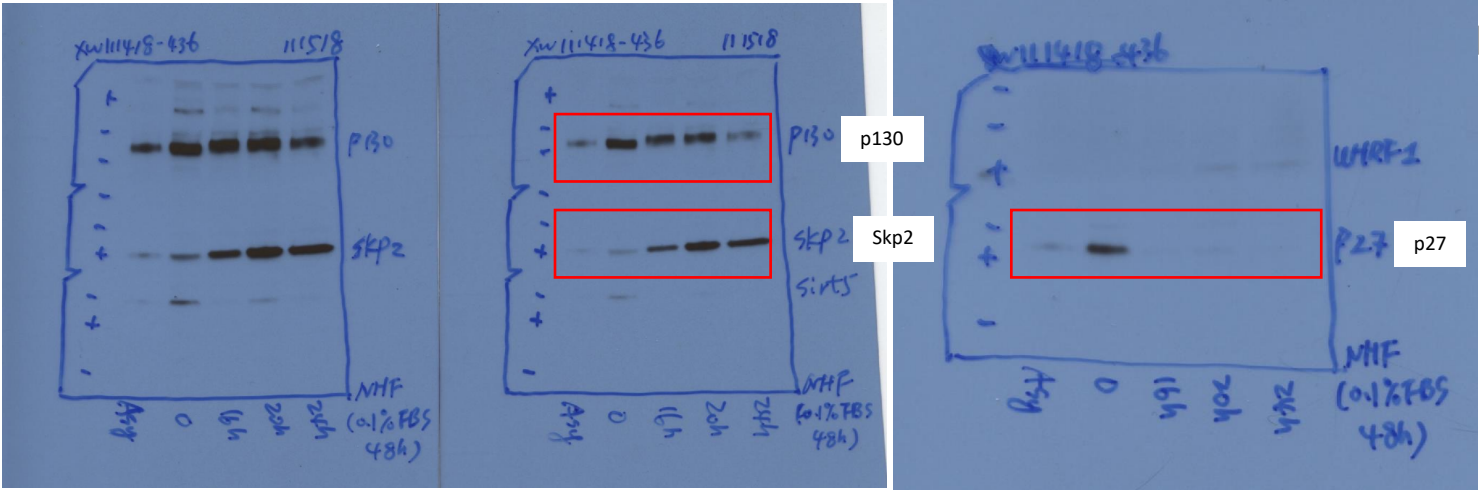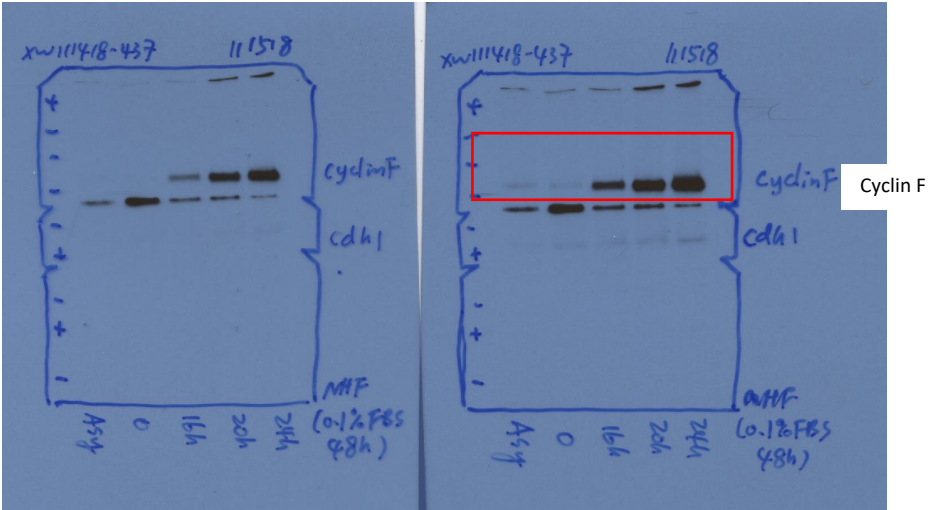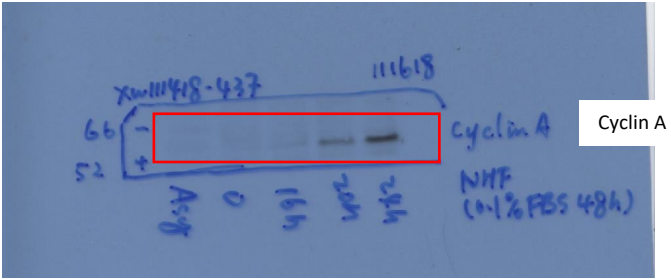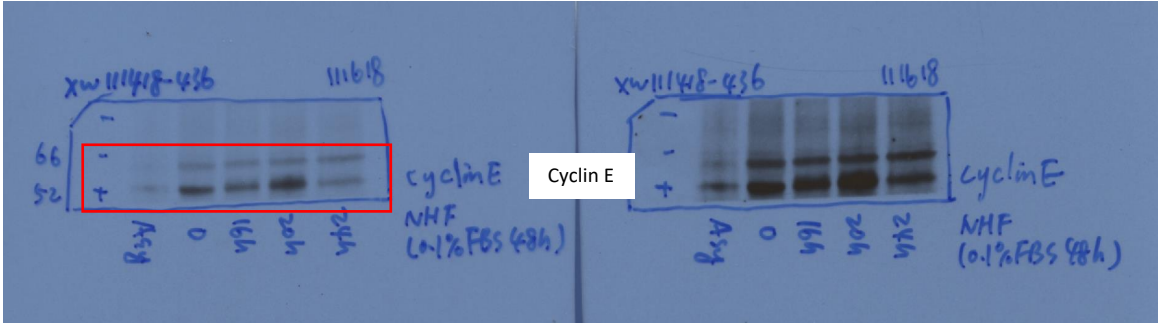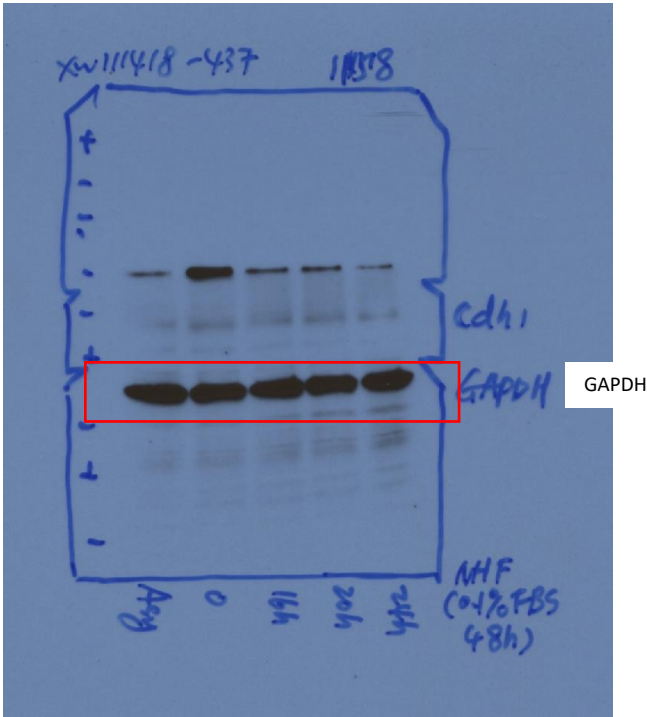

# Enrico\_Fig1 S3\_Source Data 3 For Fig1 S3D

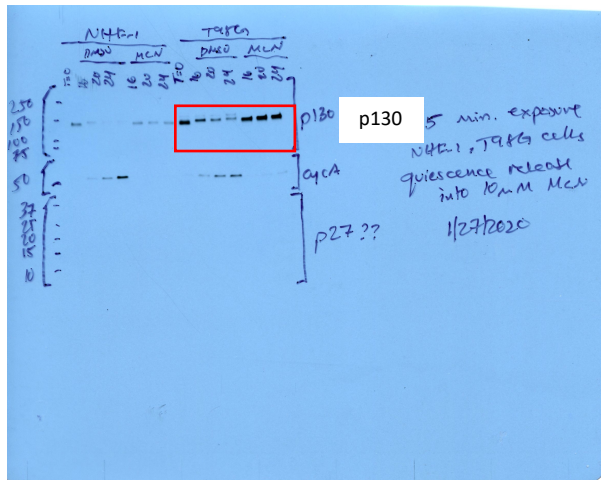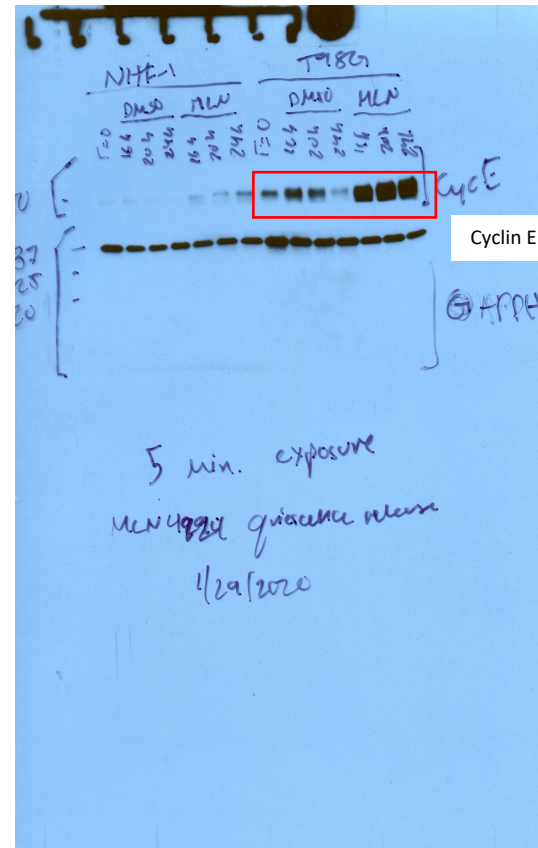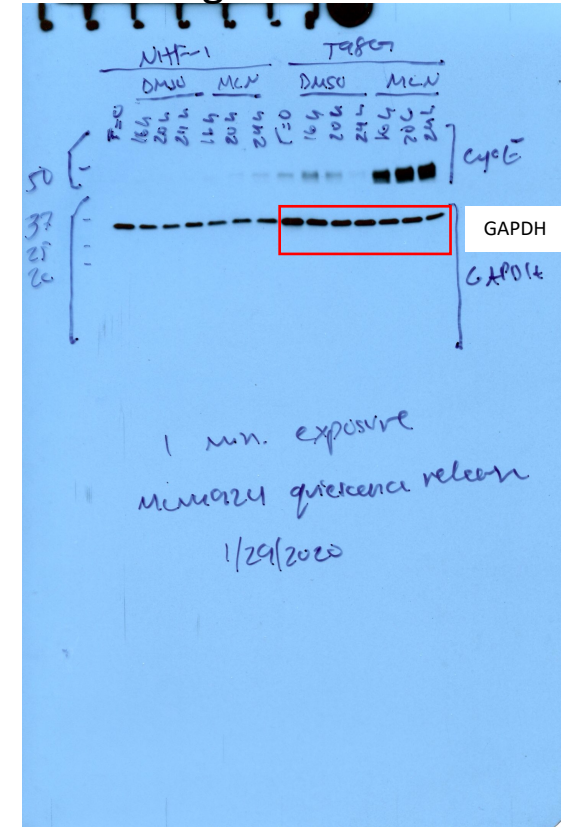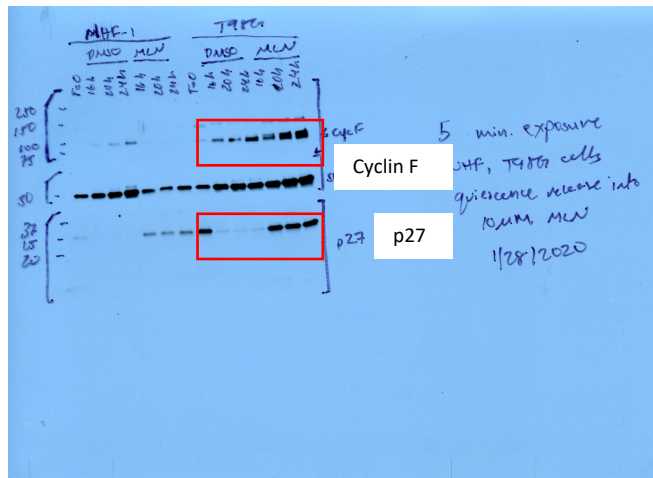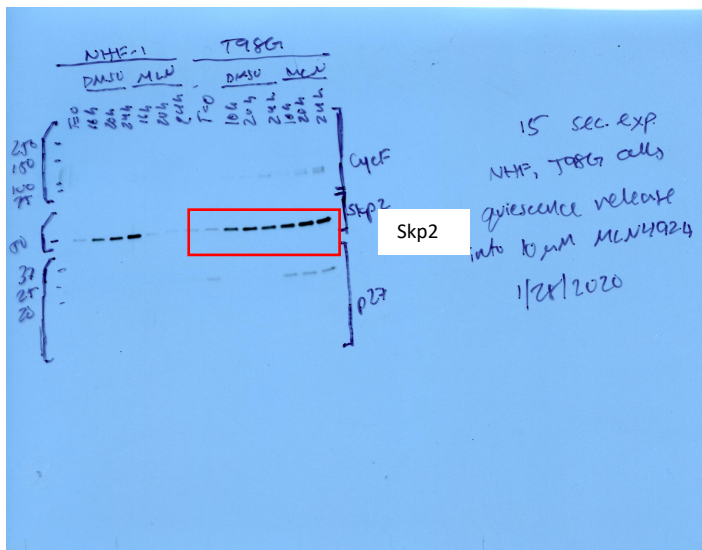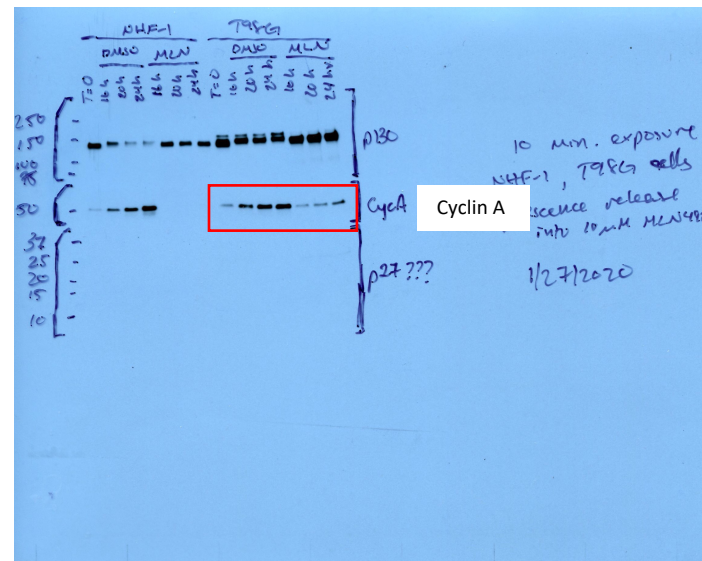

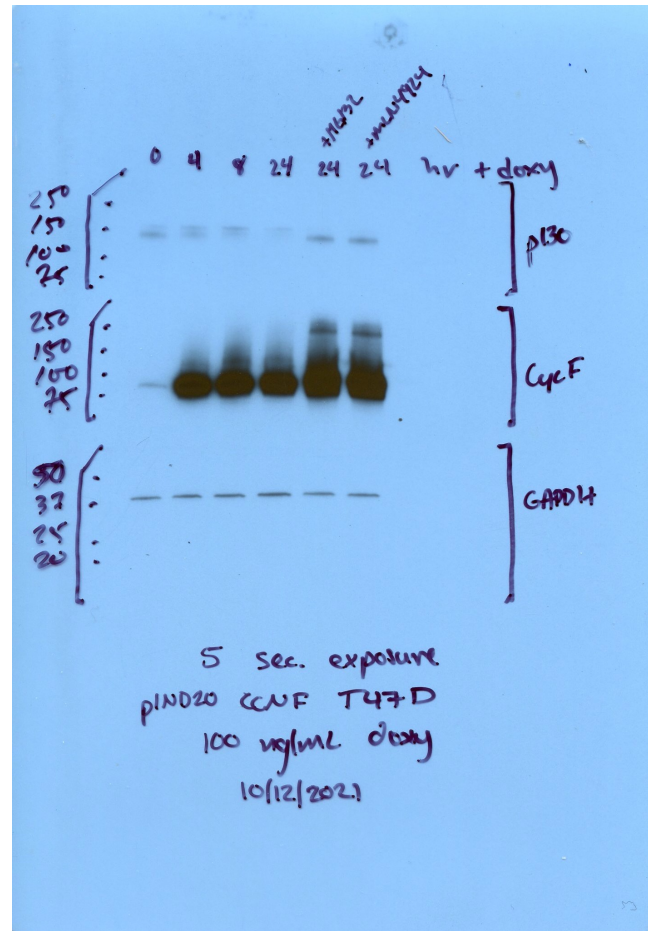

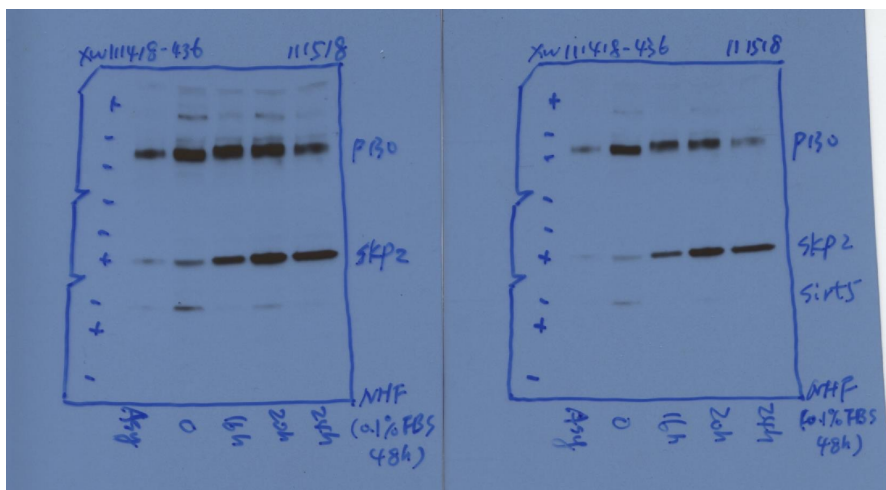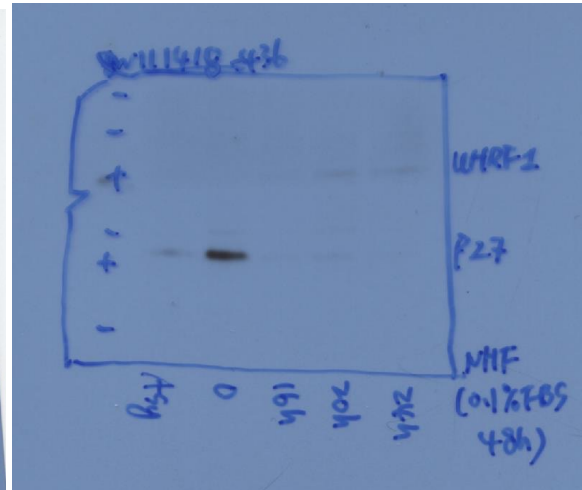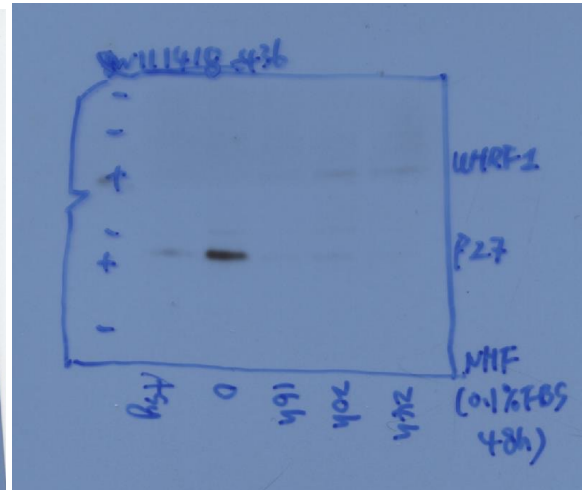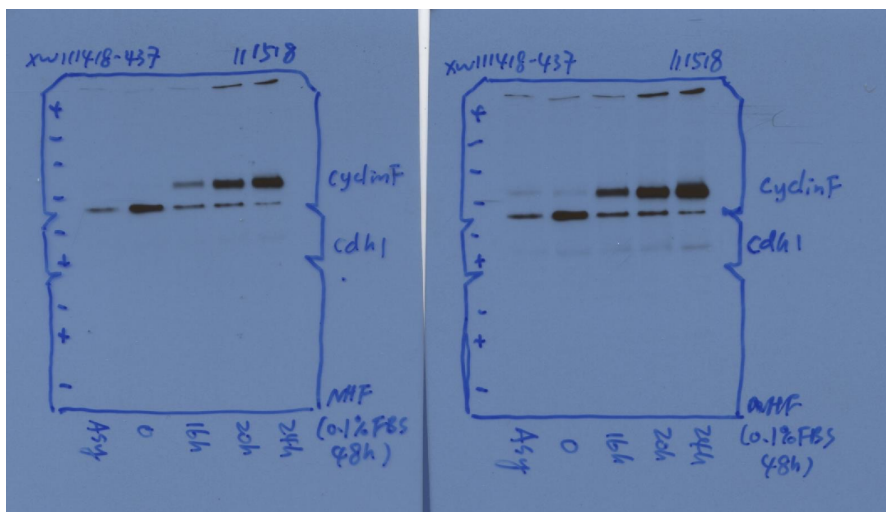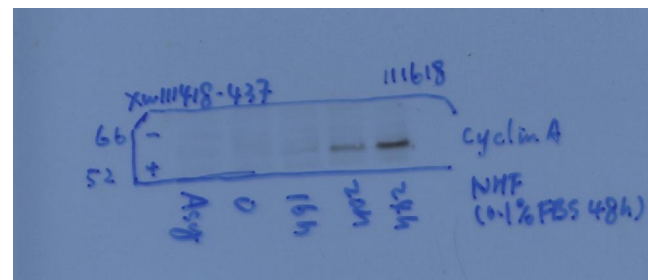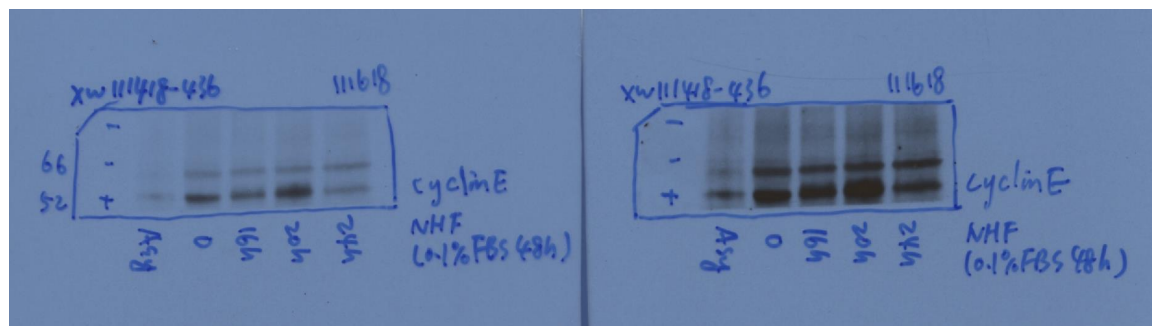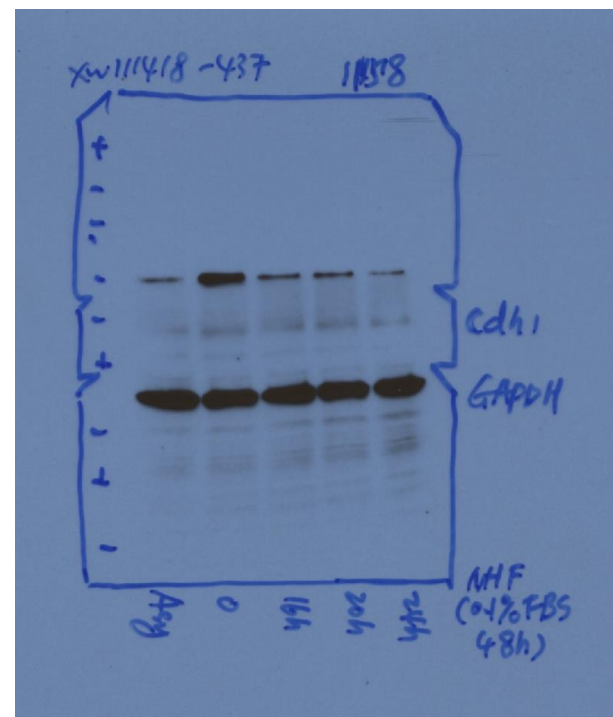

## Enrico\_Fig1 S3\_Source Data 6

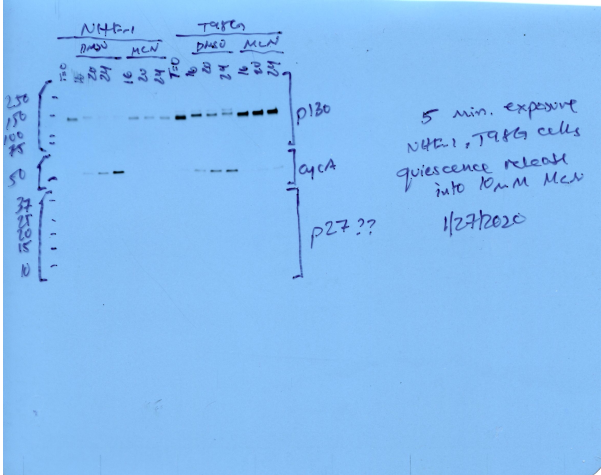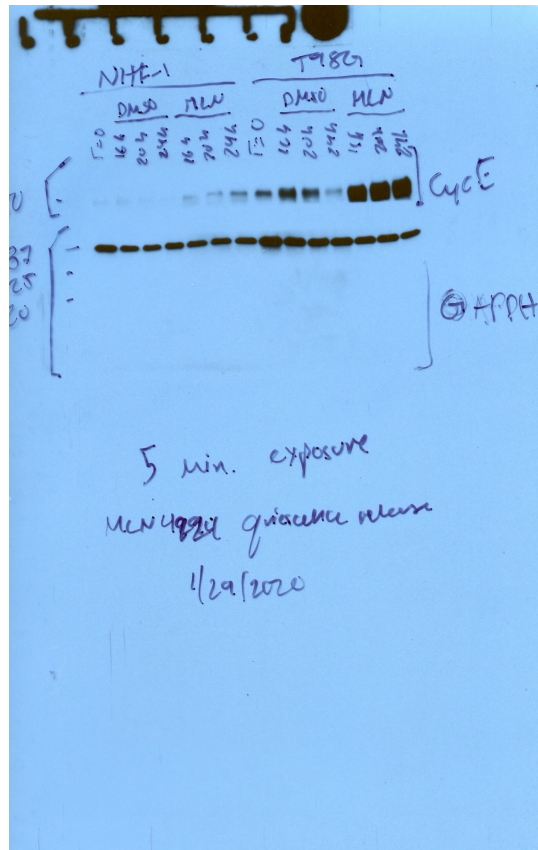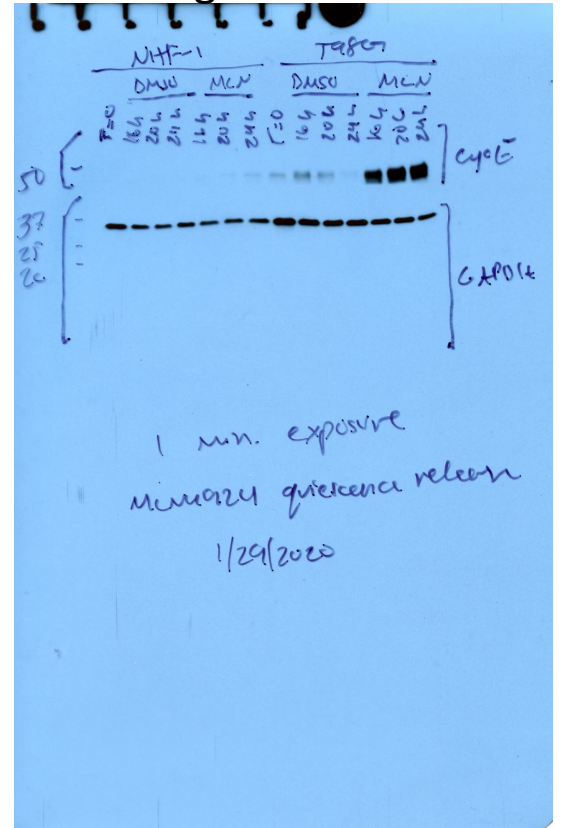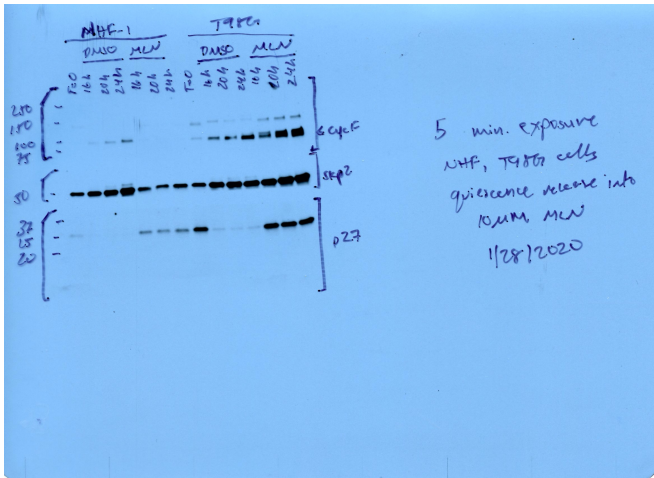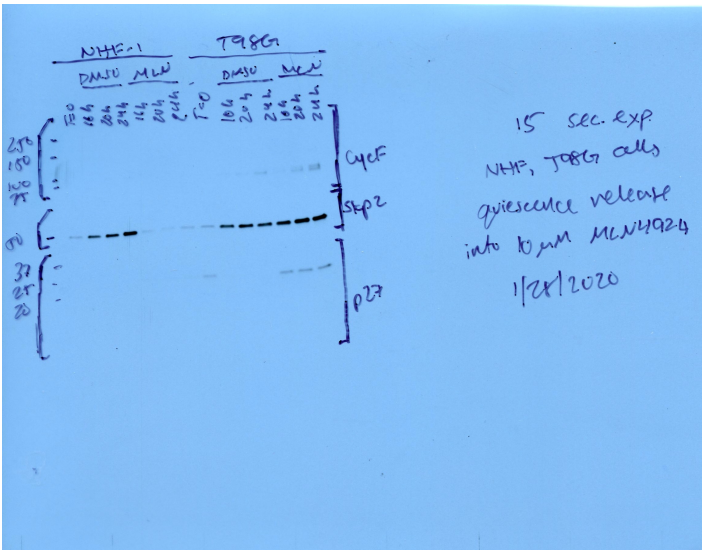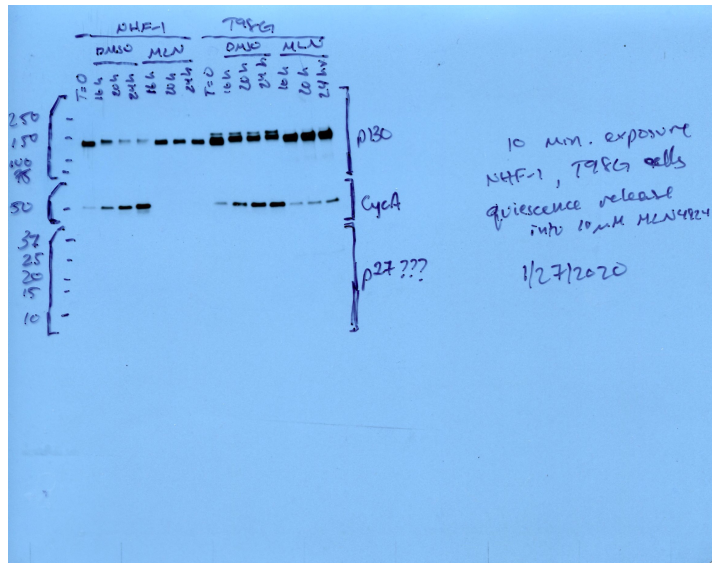

Supplement: Source data 1. — This source data file includes all uncropped blots used to generate data for the main figures and figure supplements. Additionally, copies of the uncropped images are shown a second time where blot strips shown in figures are highlighted with a red square and the protein that was blotted for is noted. [file elife-70691-supp2.zip › Source Data 1/Enrico-Fig1S3-source-data.pdf]

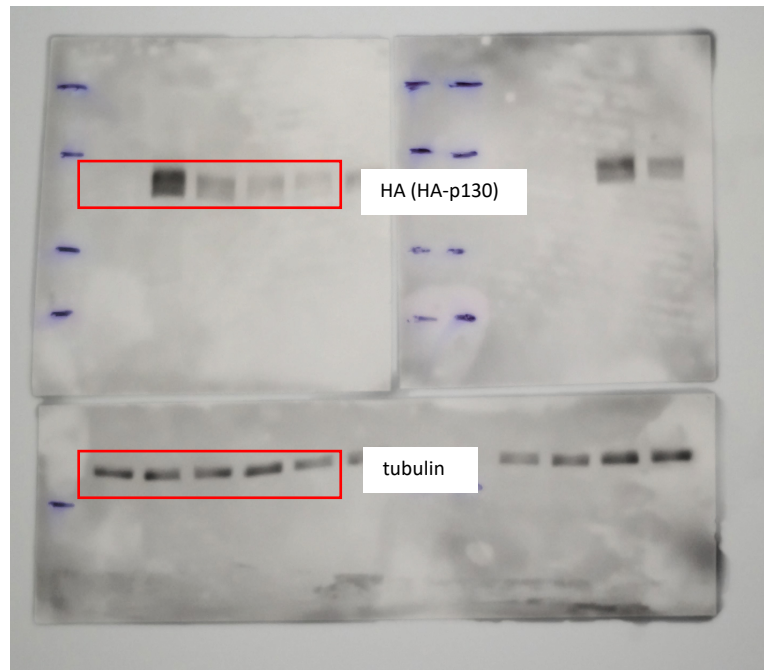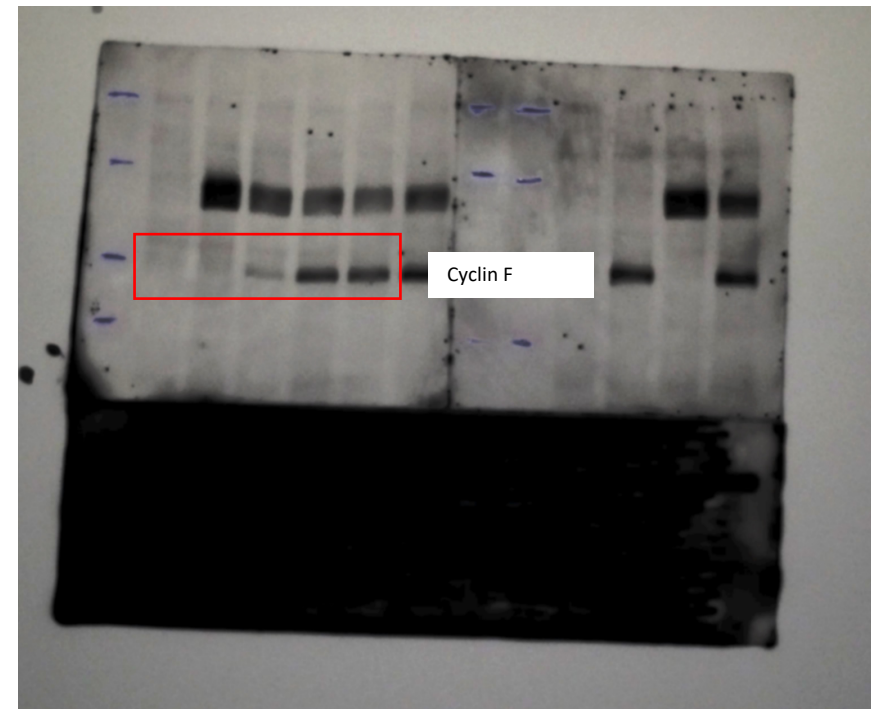

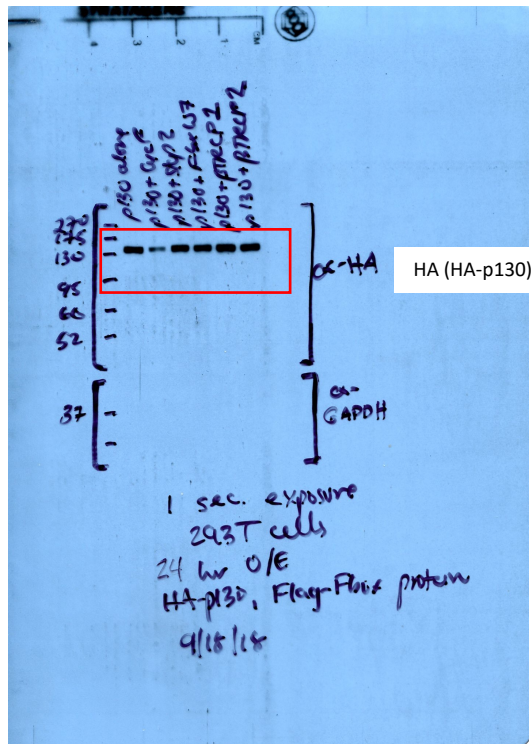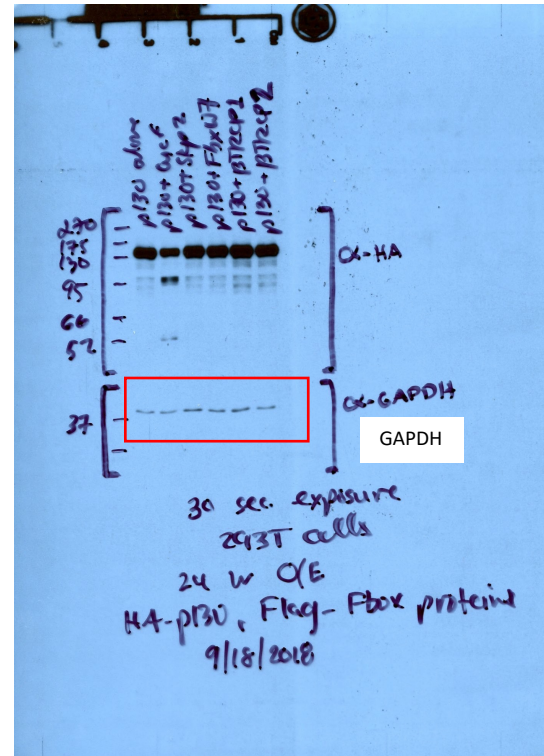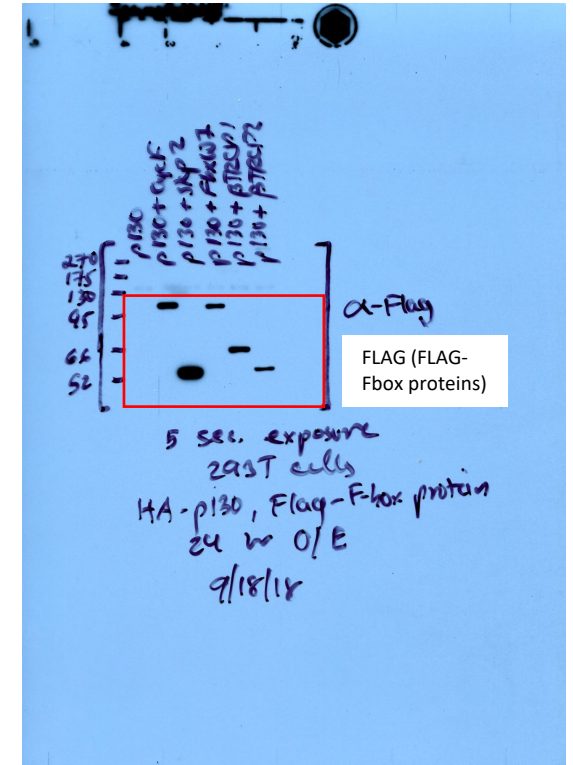

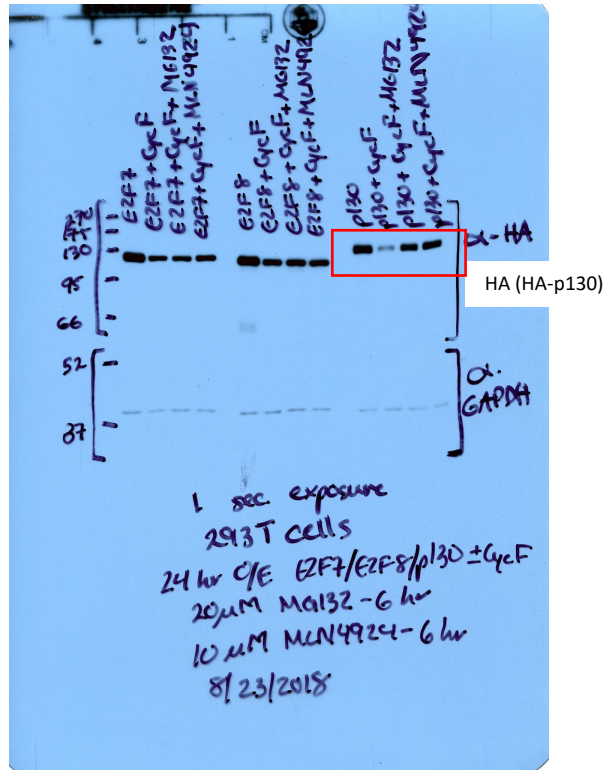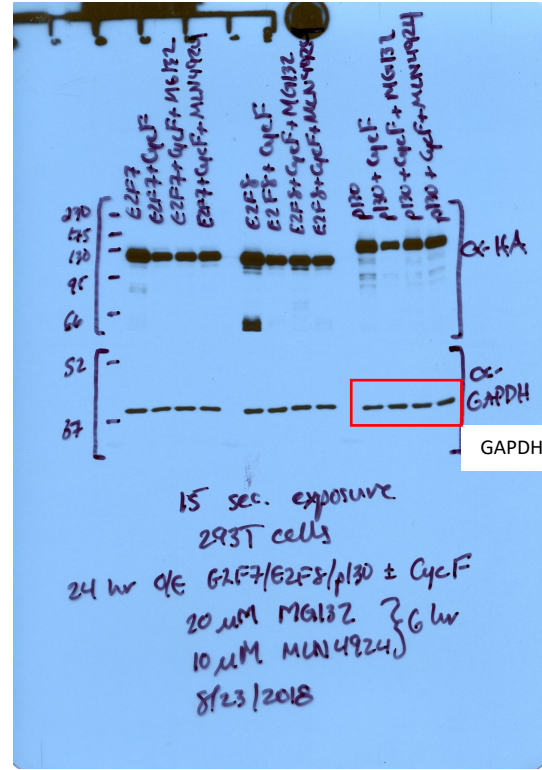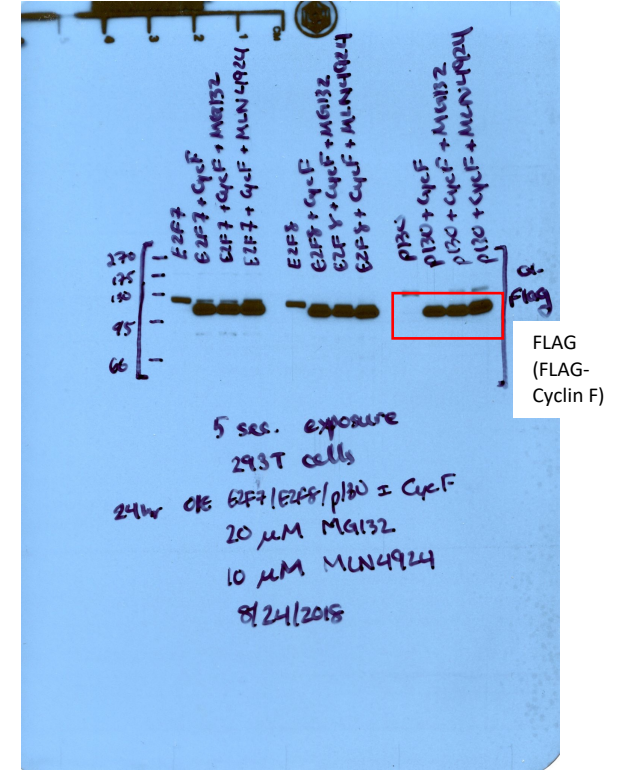

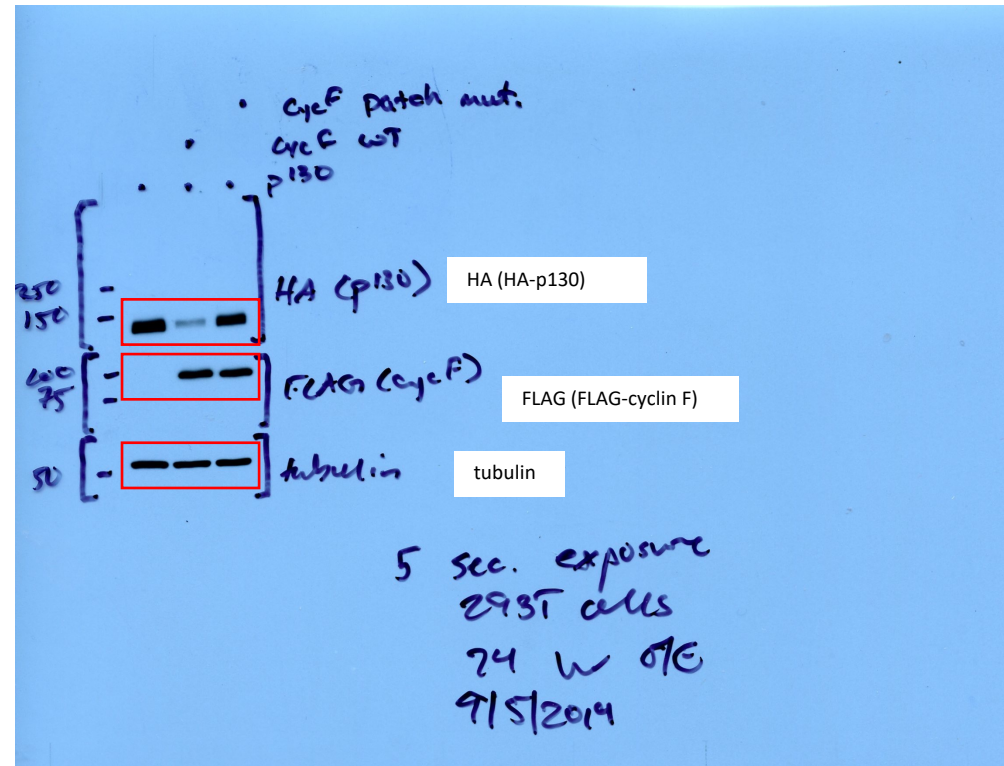

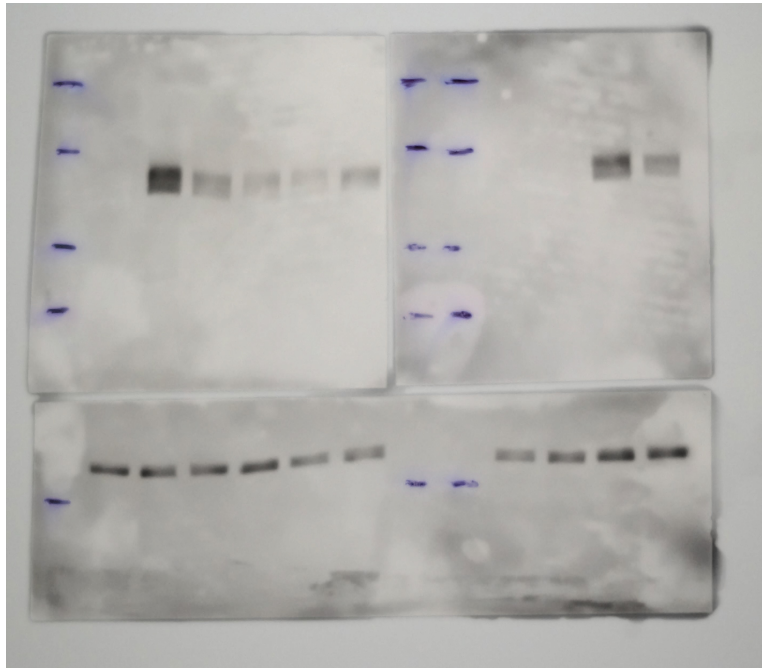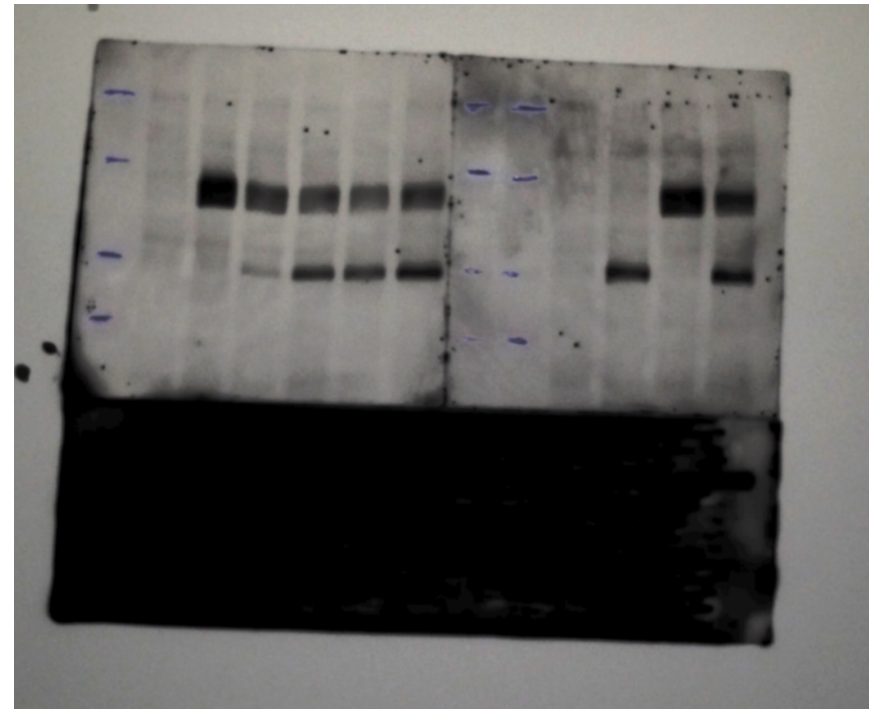

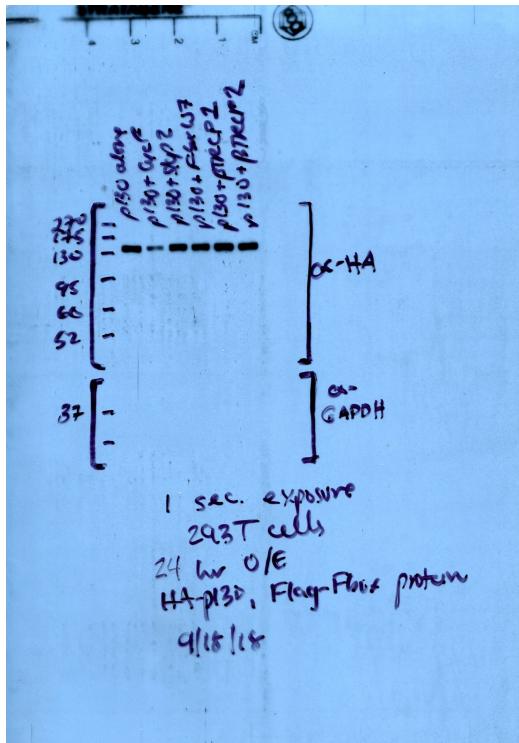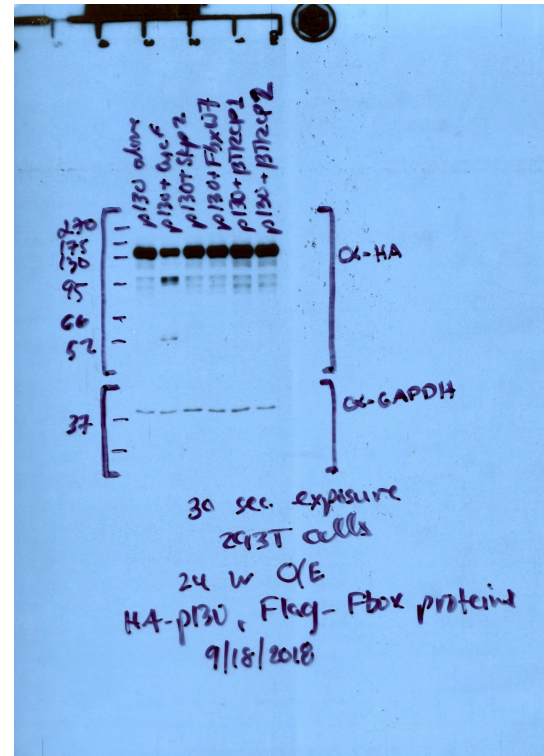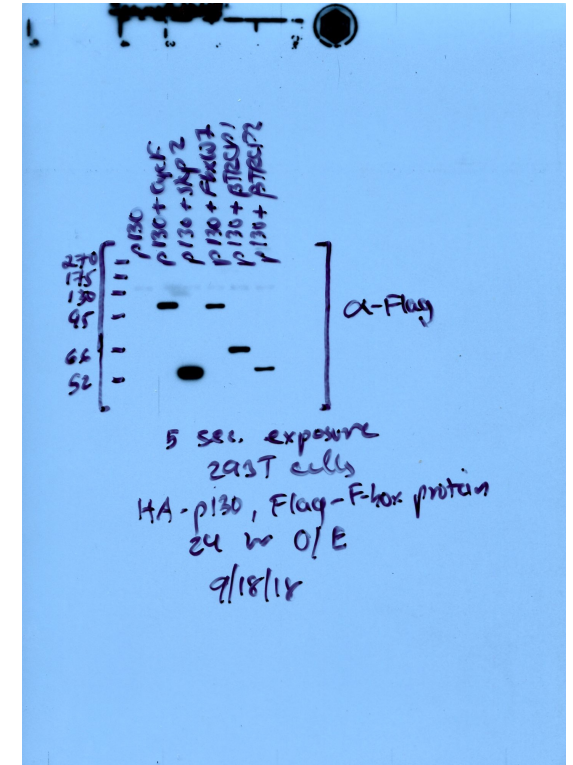

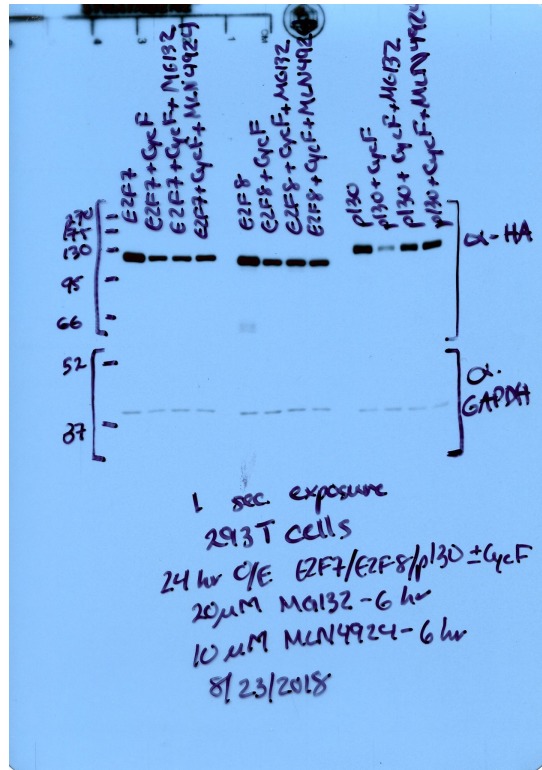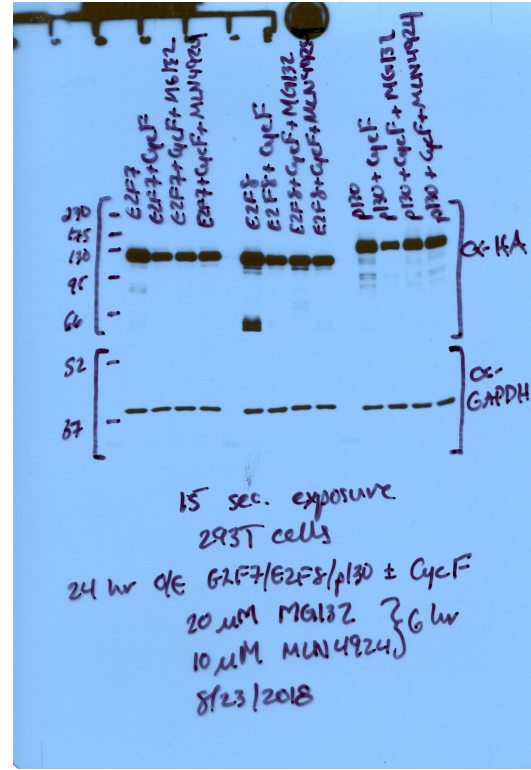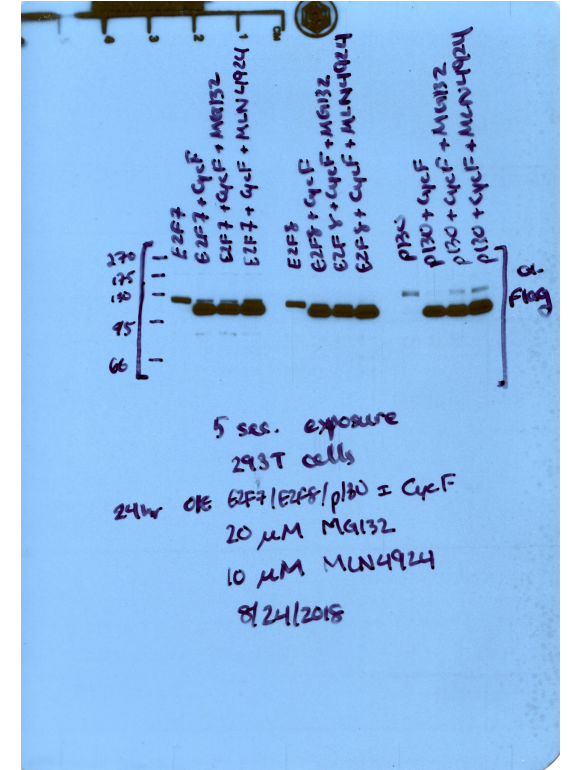

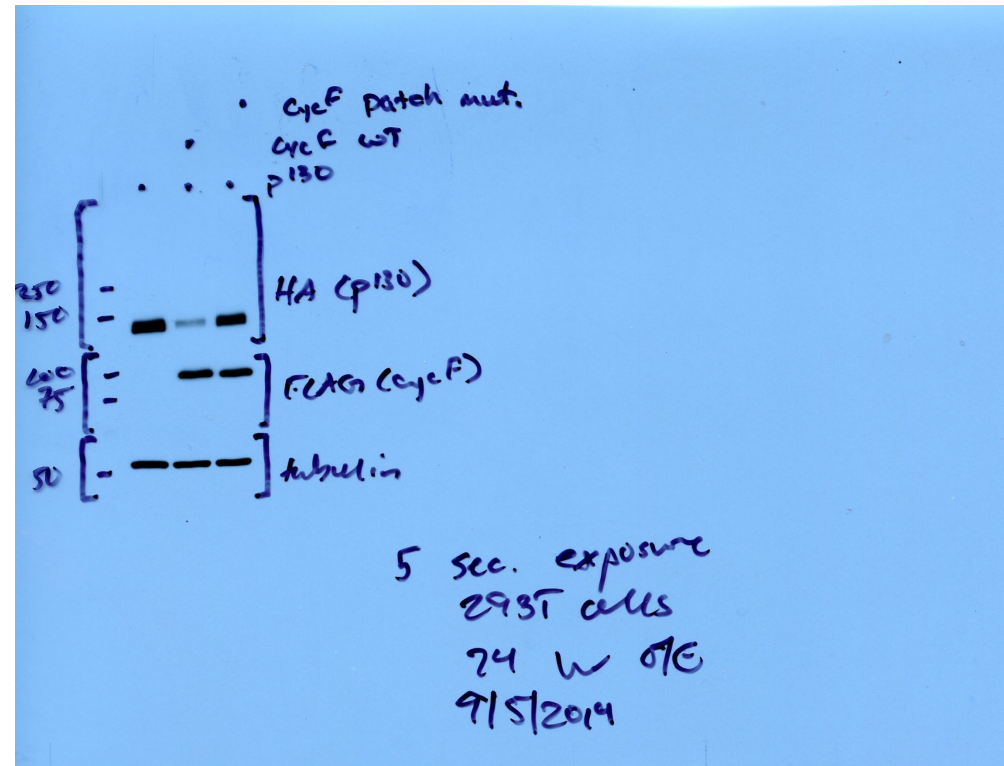

Supplement: Source data 1. — This source data file includes all uncropped blots used to generate data for the main figures and figure supplements. Additionally, copies of the uncropped images are shown a second time where blot strips shown in figures are highlighted with a red square and the protein that was blotted for is noted. [file elife-70691-supp2.zip › Source Data 1/Enrico-Fig3-source-data.pdf]

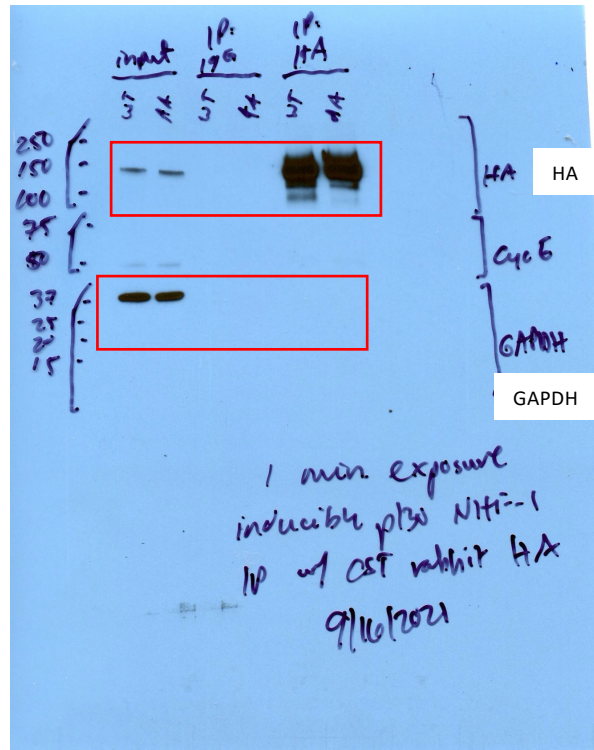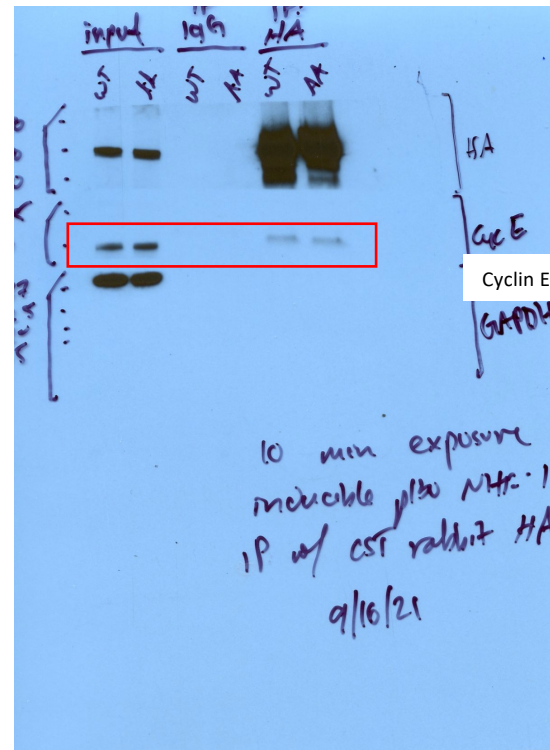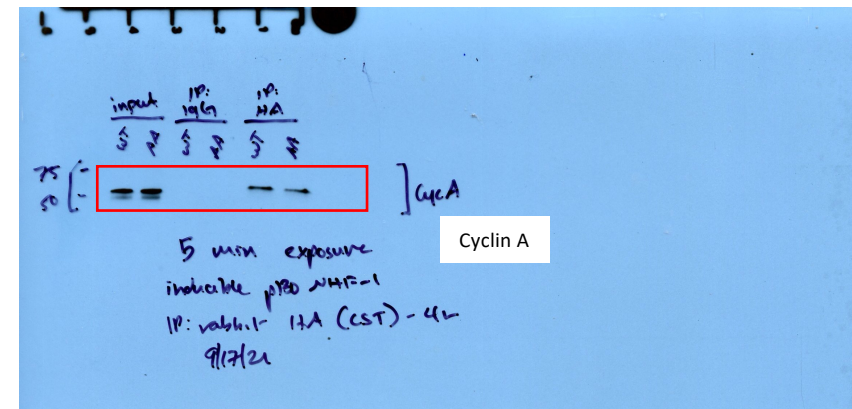

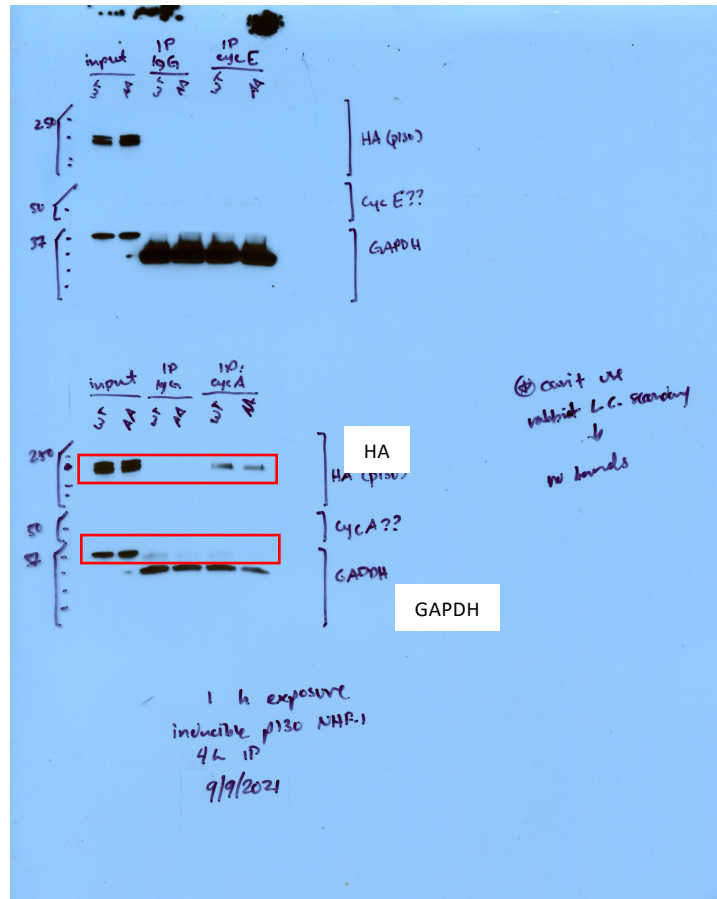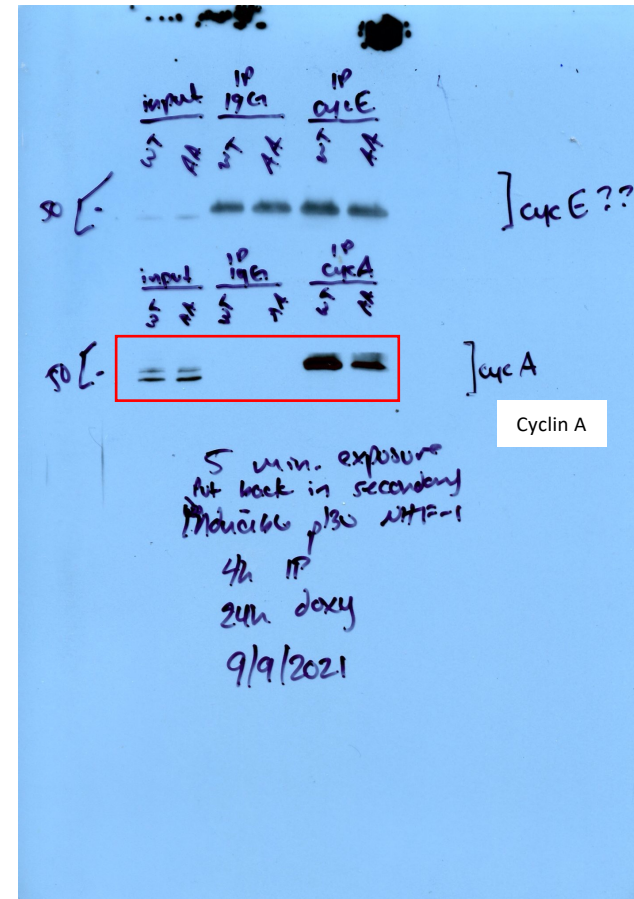

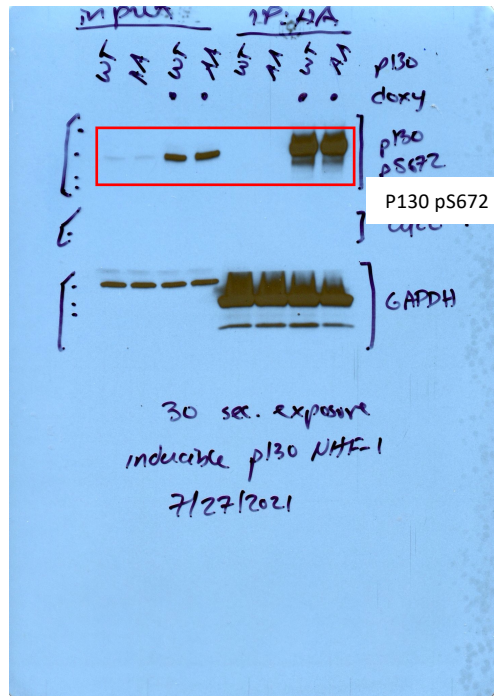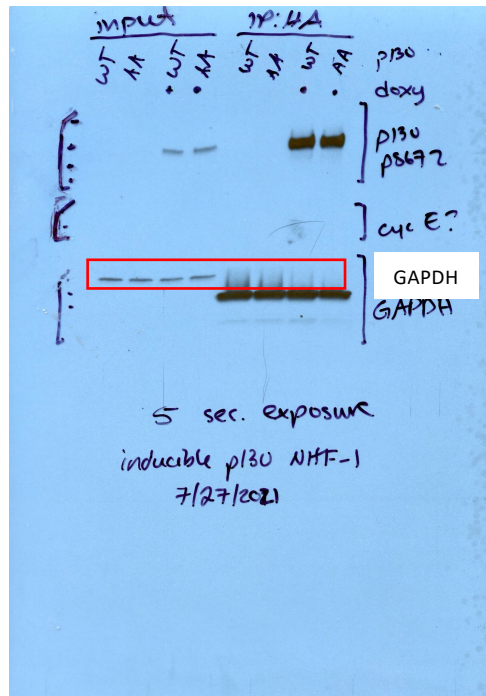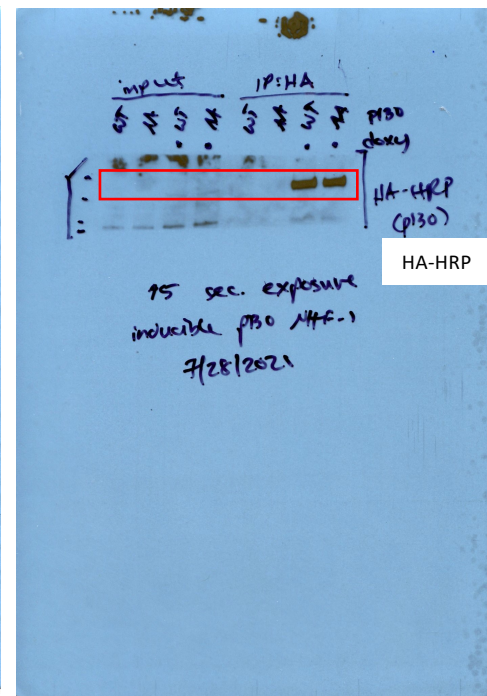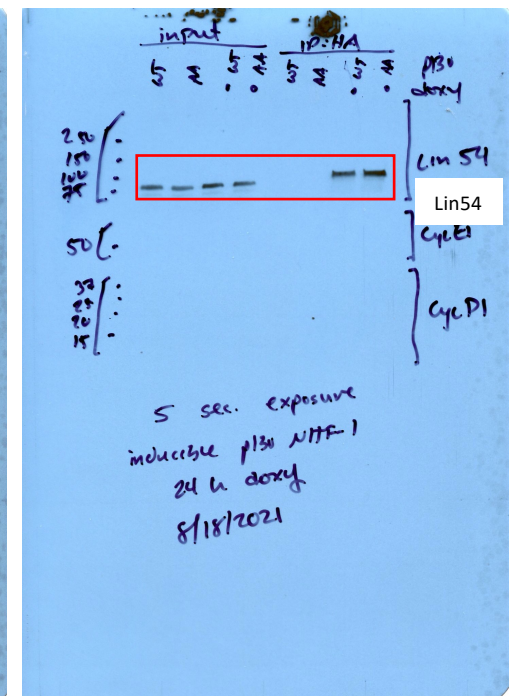

\*blotted membrane from 7/28 on  
8/18 once Lin54 antibody arrived

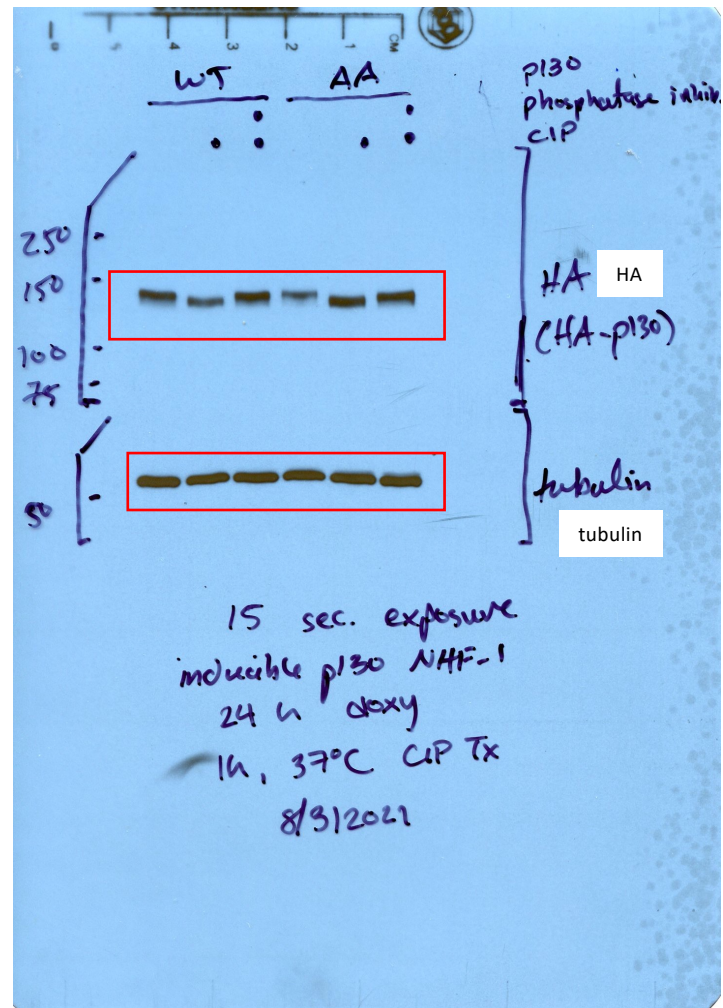

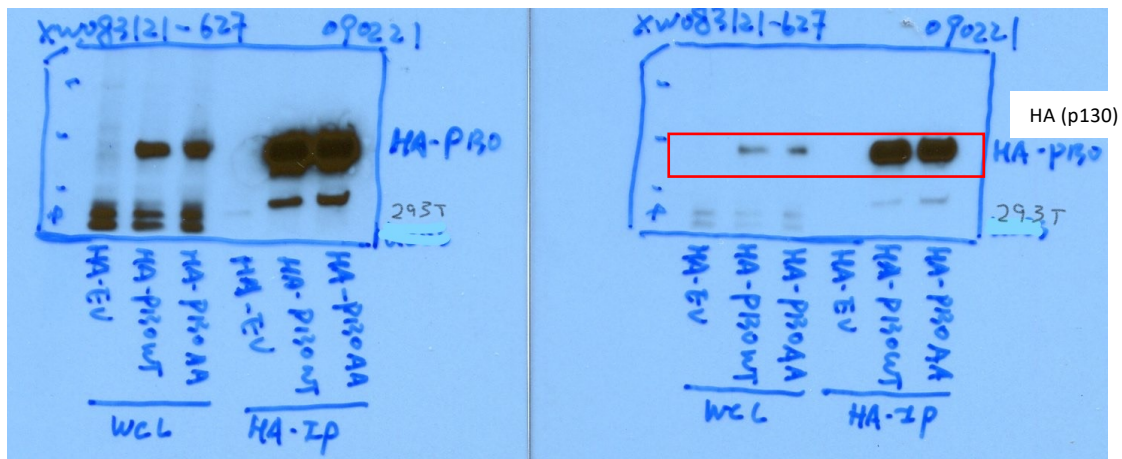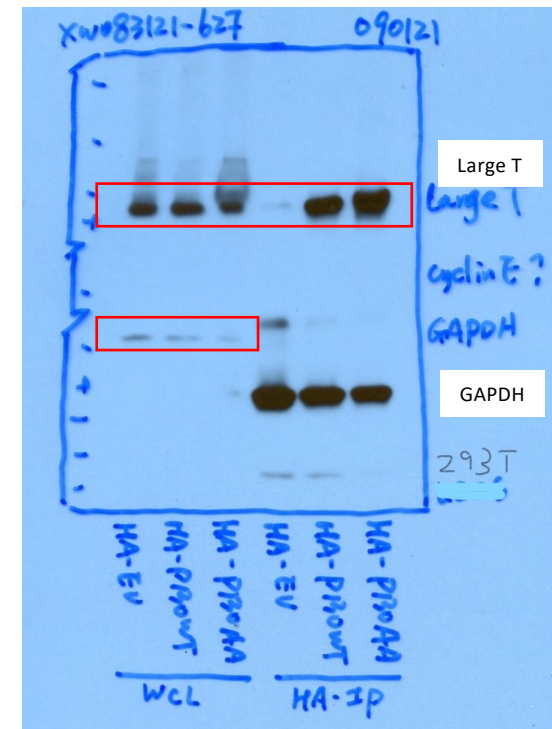

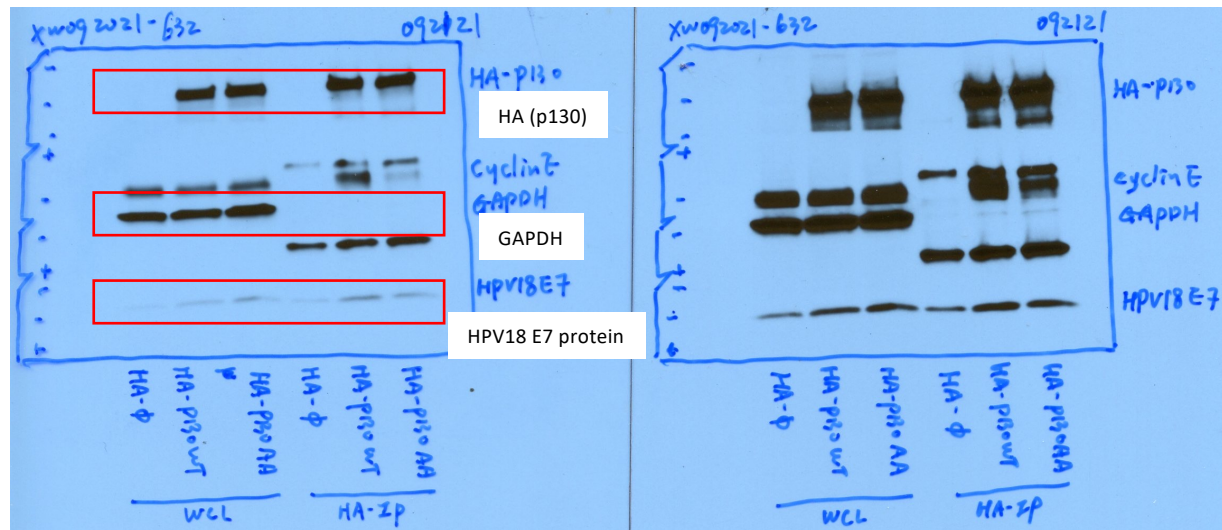

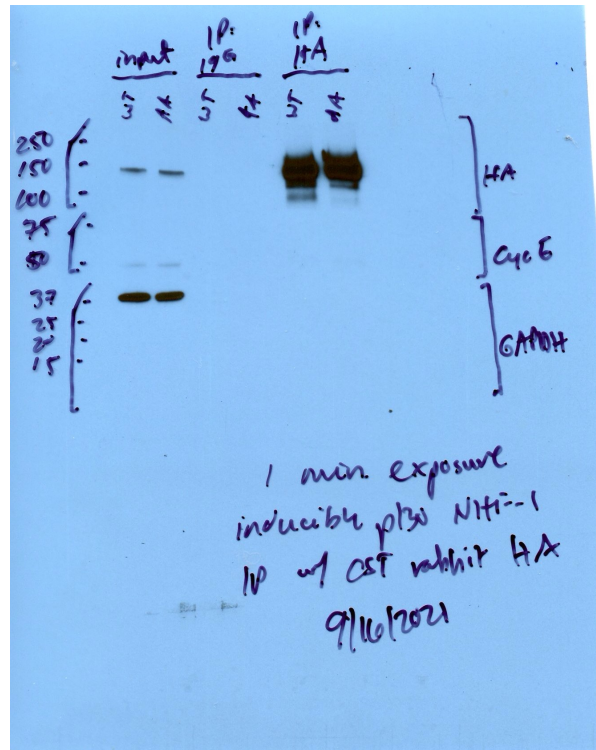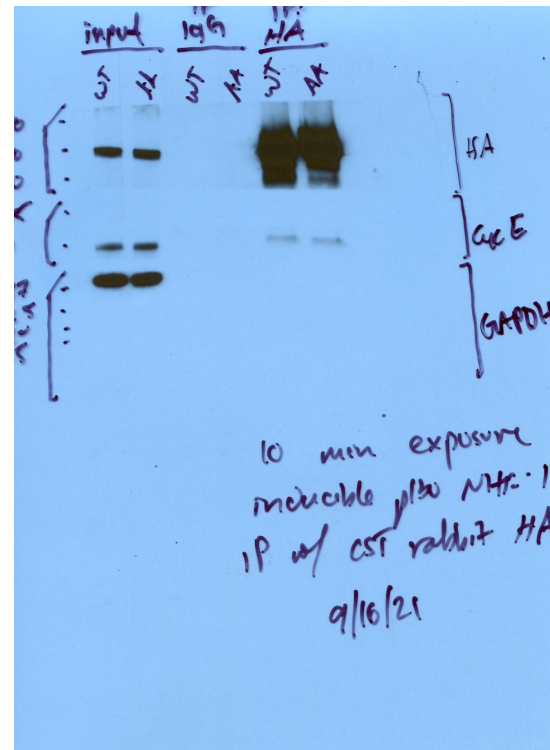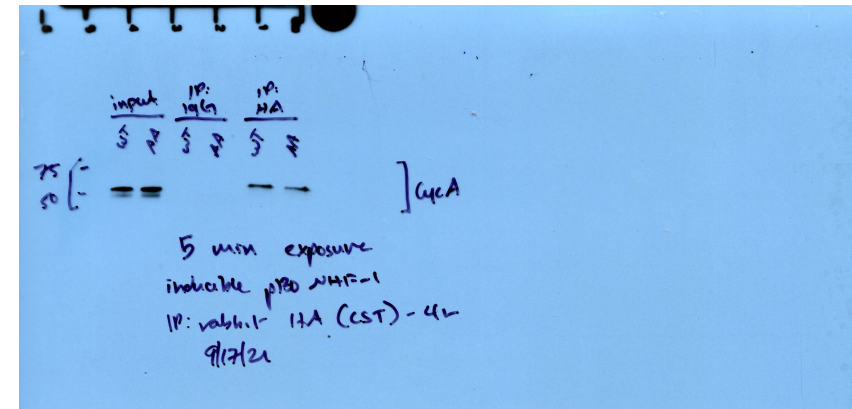

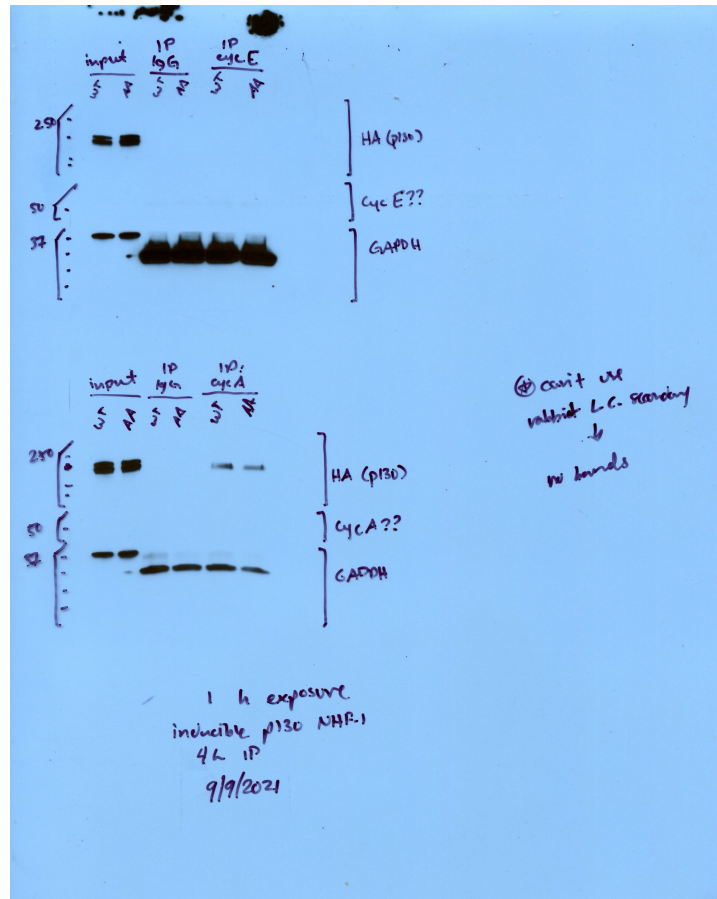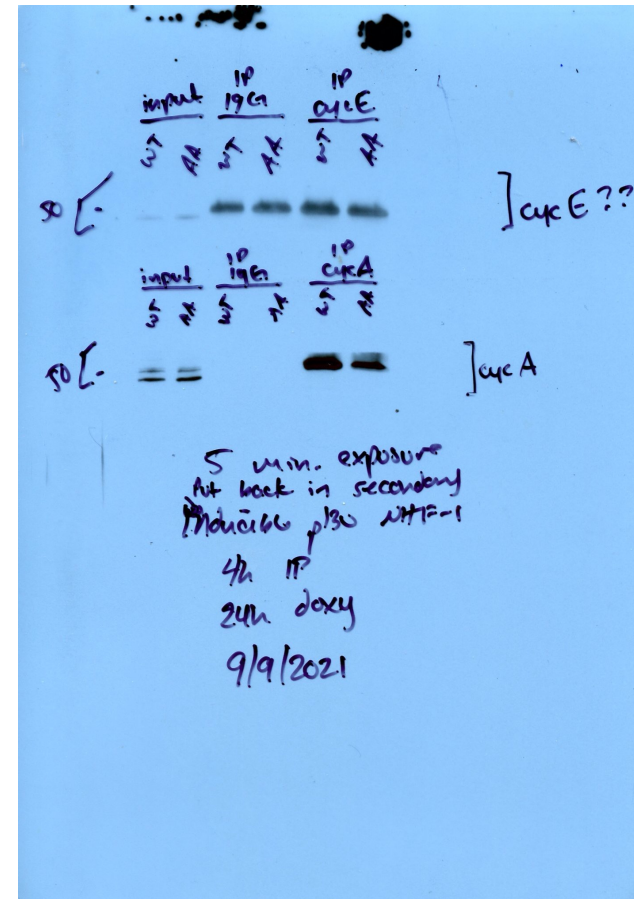

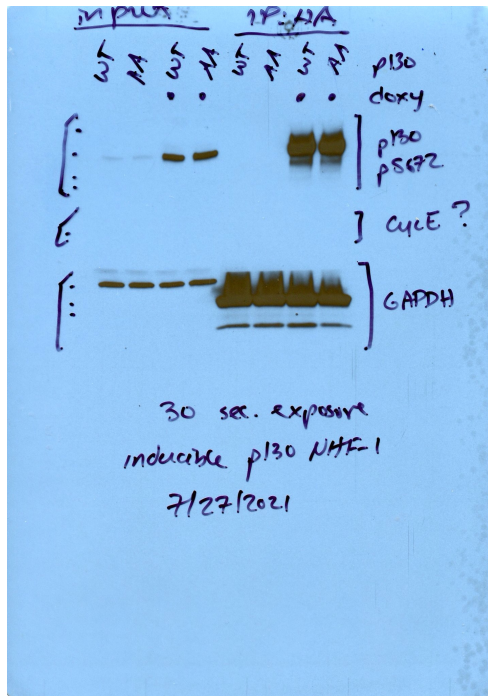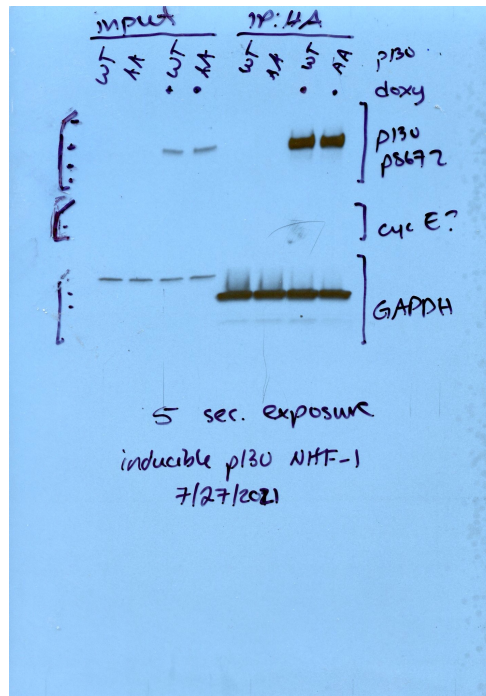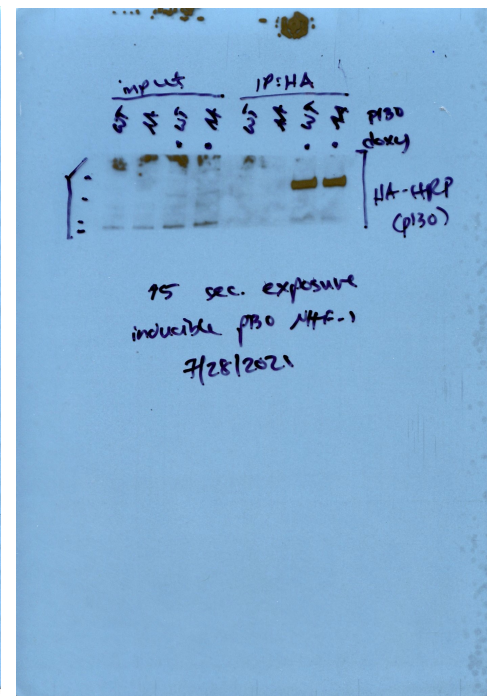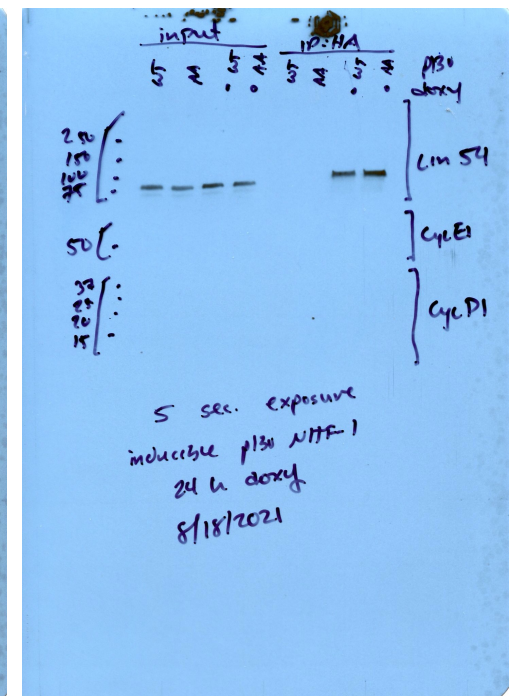

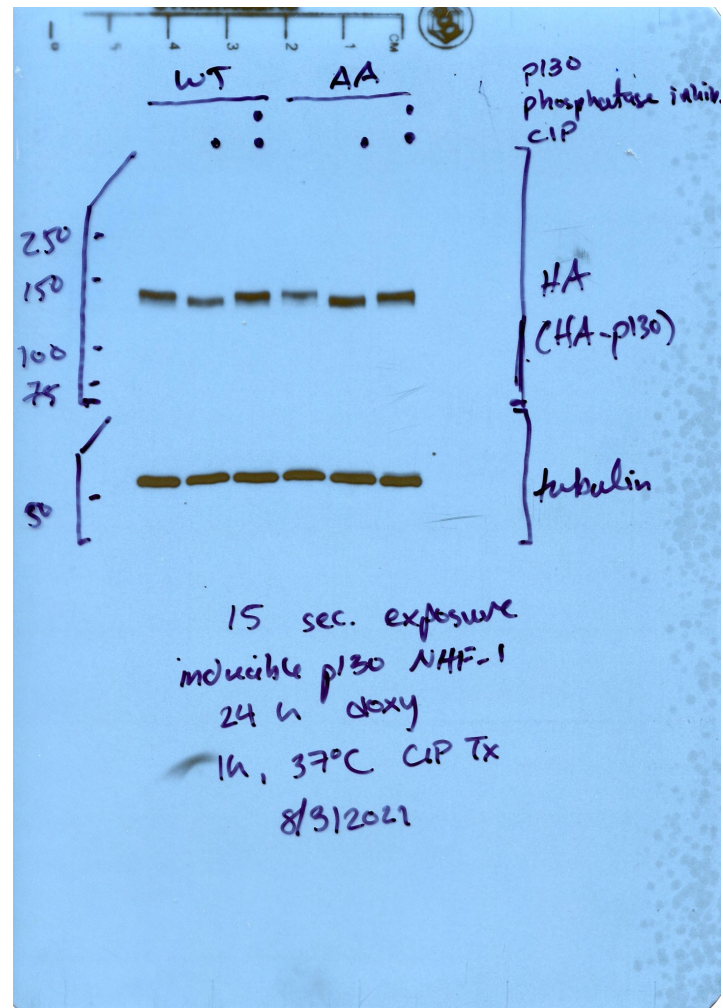

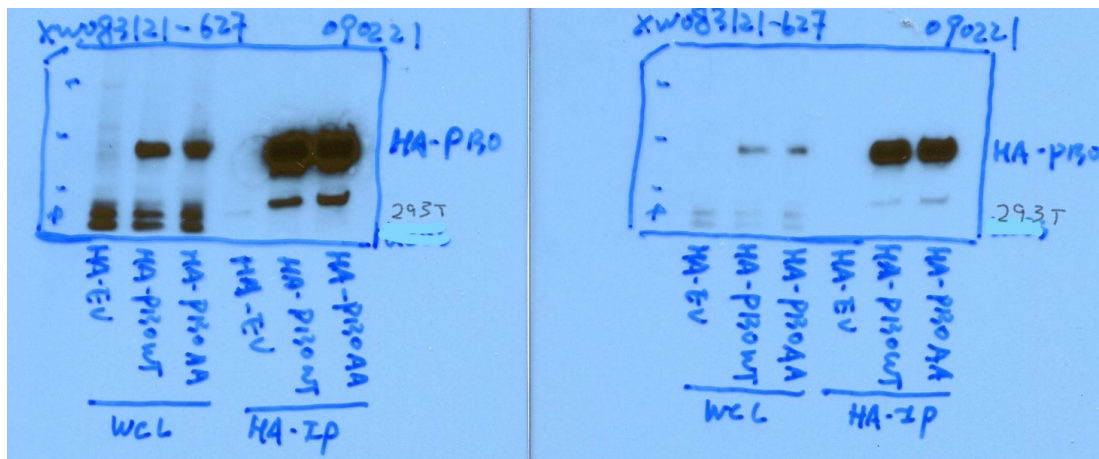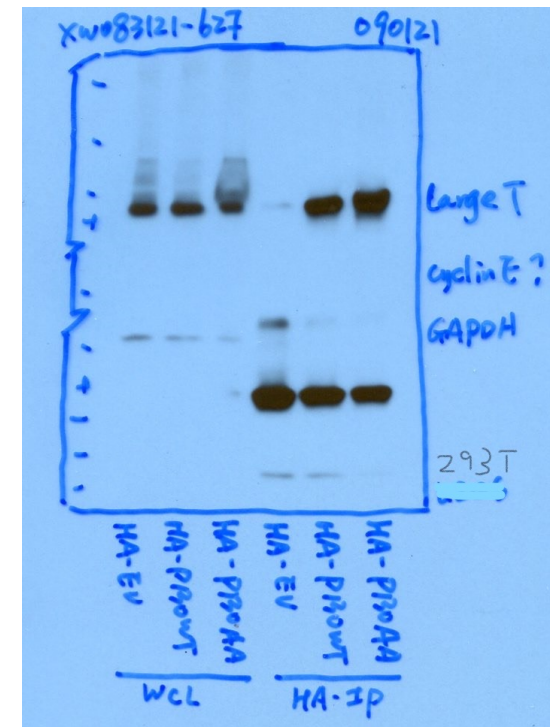

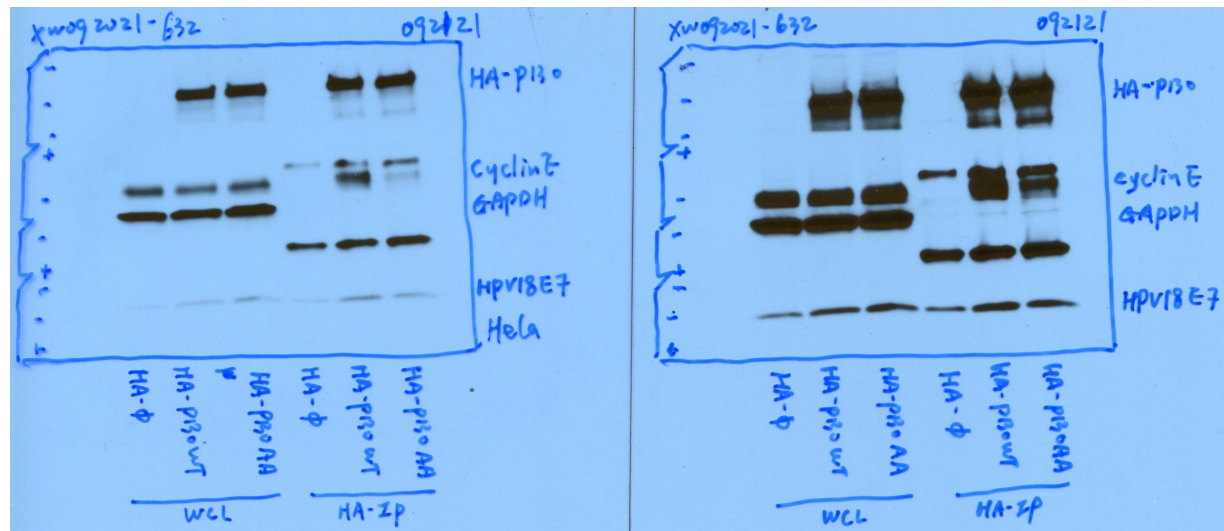

Supplement: Source data 1. — This source data file includes all uncropped blots used to generate data for the main figures and figure supplements. Additionally, copies of the uncropped images are shown a second time where blot strips shown in figures are highlighted with a red square and the protein that was blotted for is noted. [file elife-70691-supp2.zip › Source Data 1/Enrico-Fig5S2-source-data.pdf]

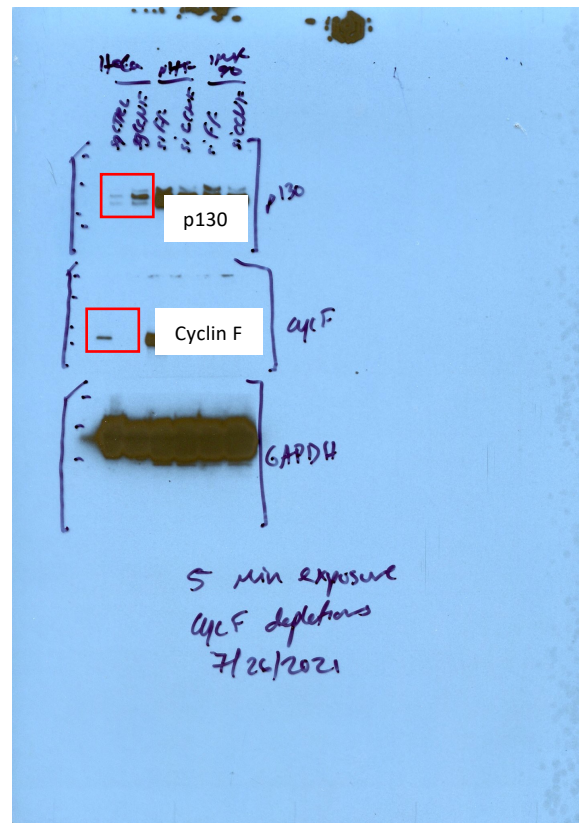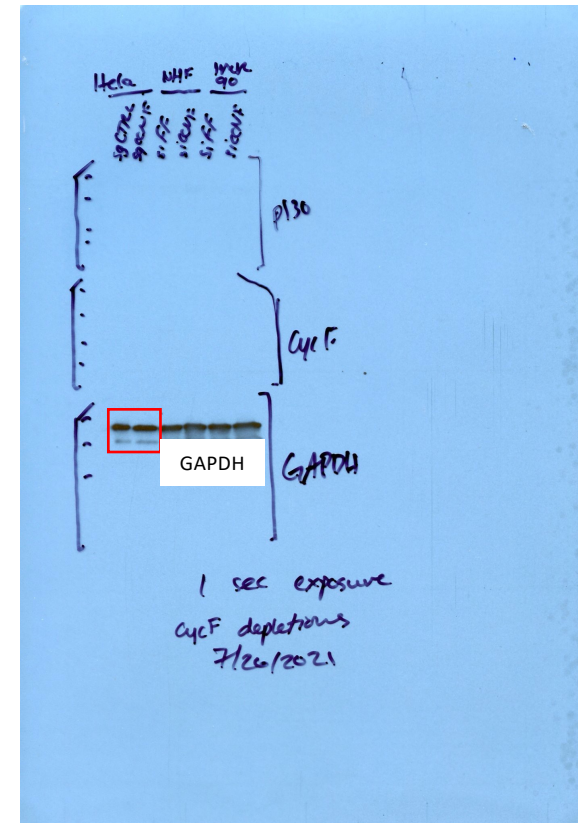

For the NHF-1 cells:

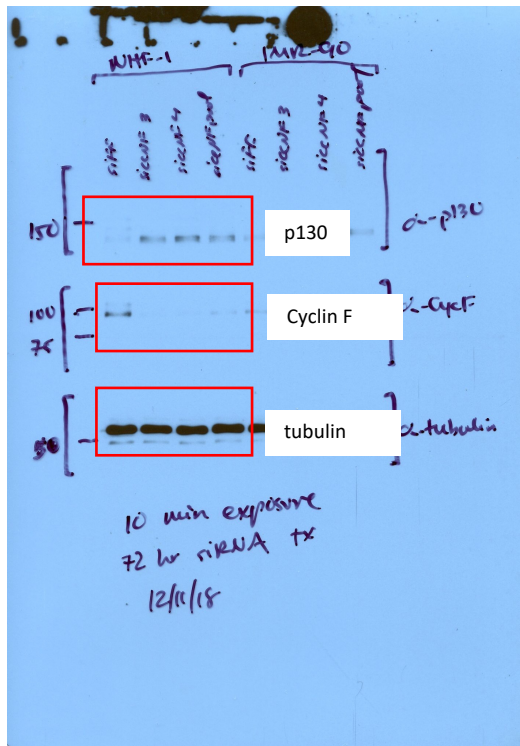

For the IMR-90 cells:

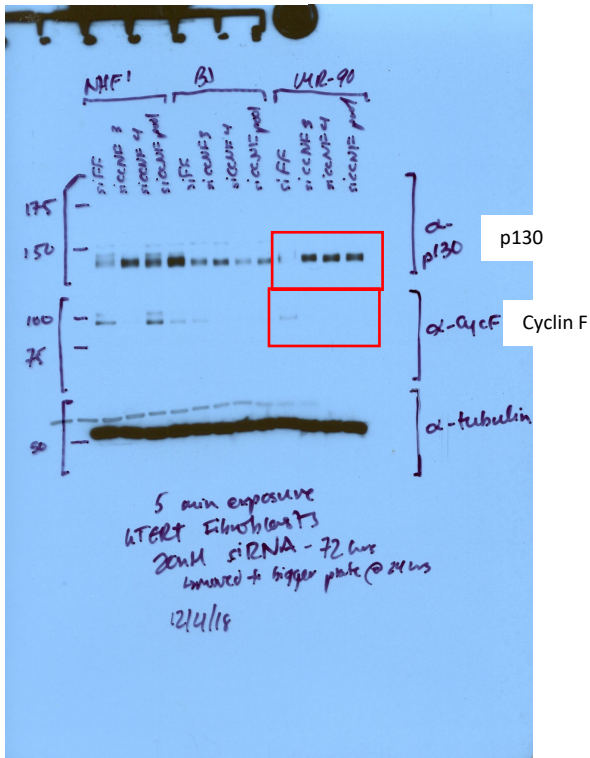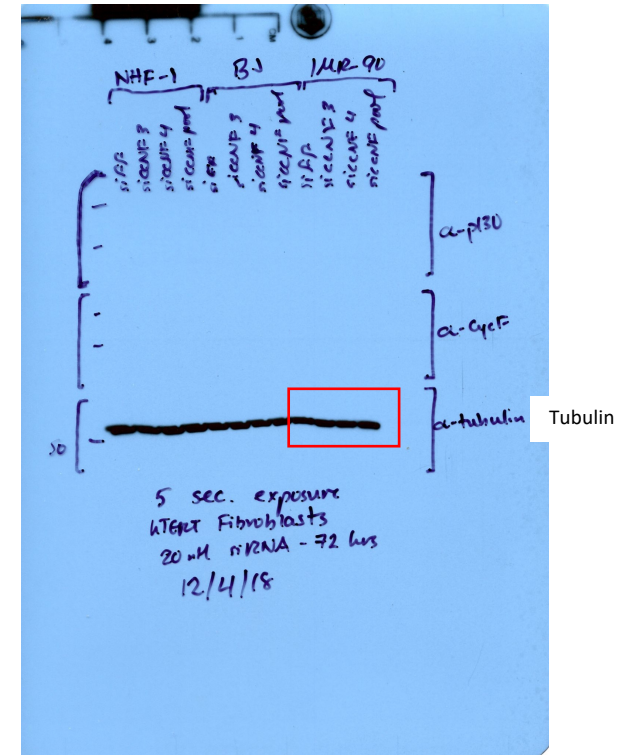

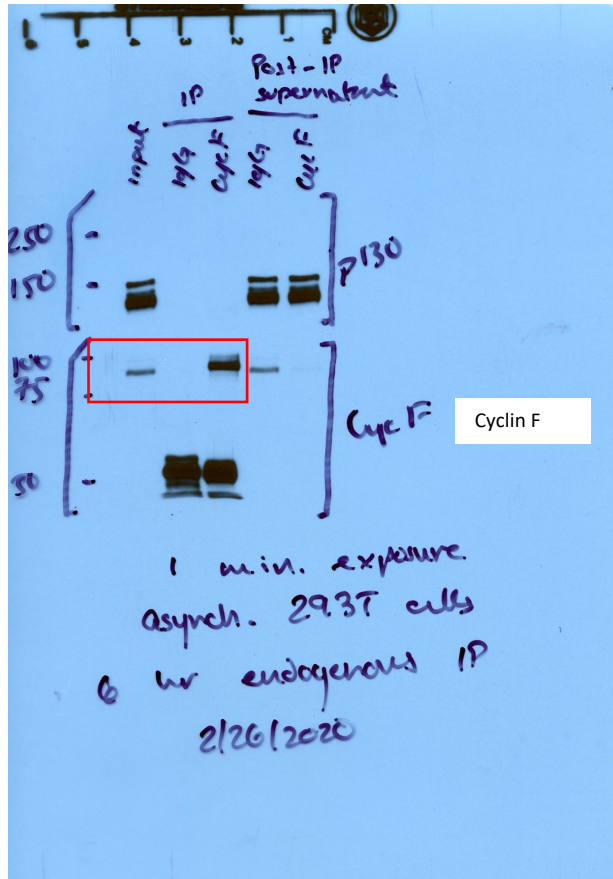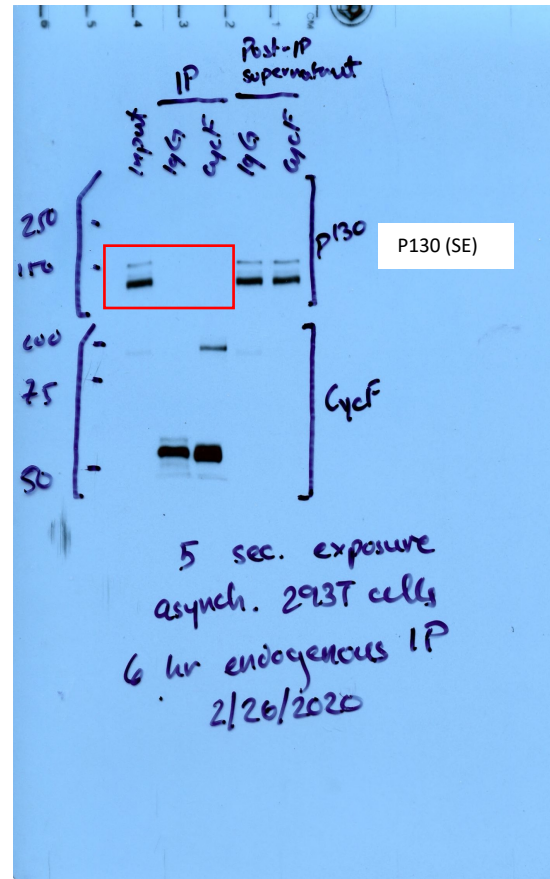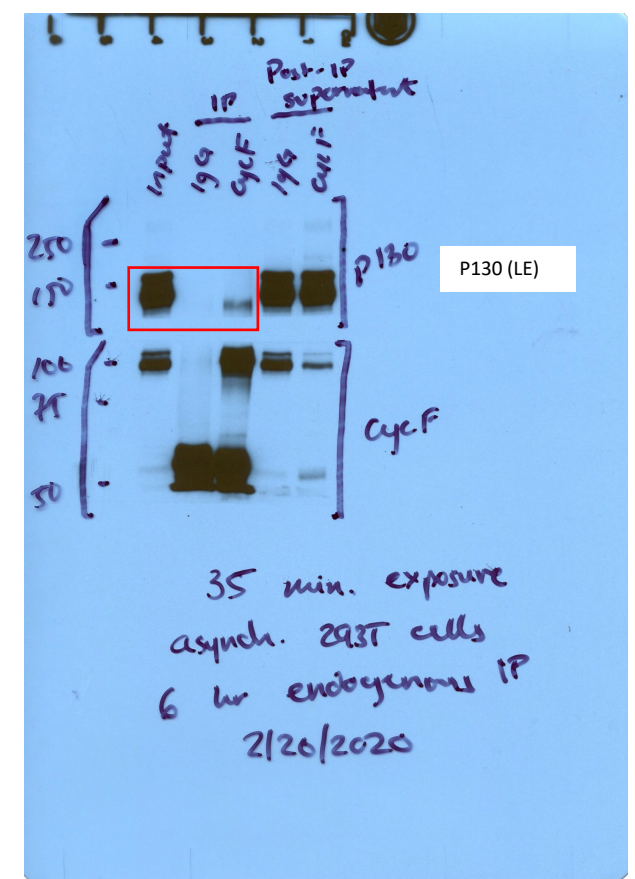

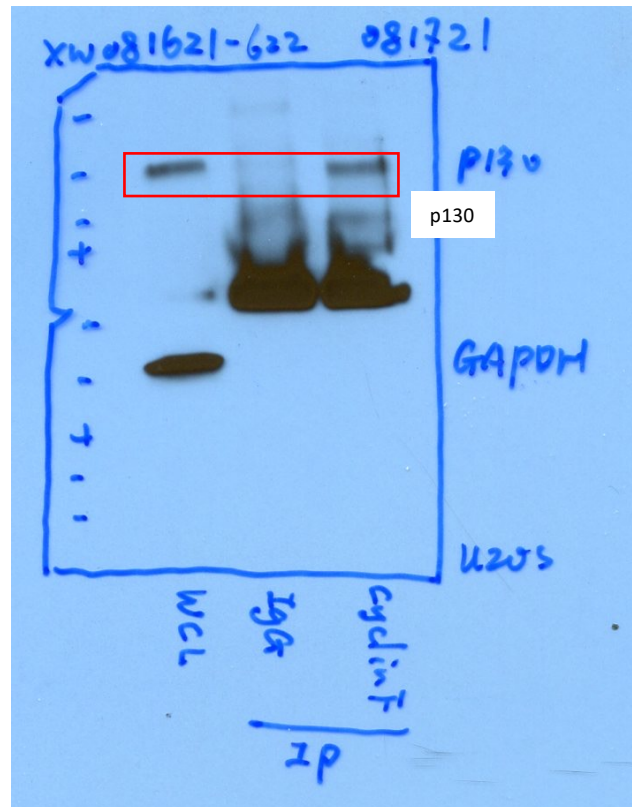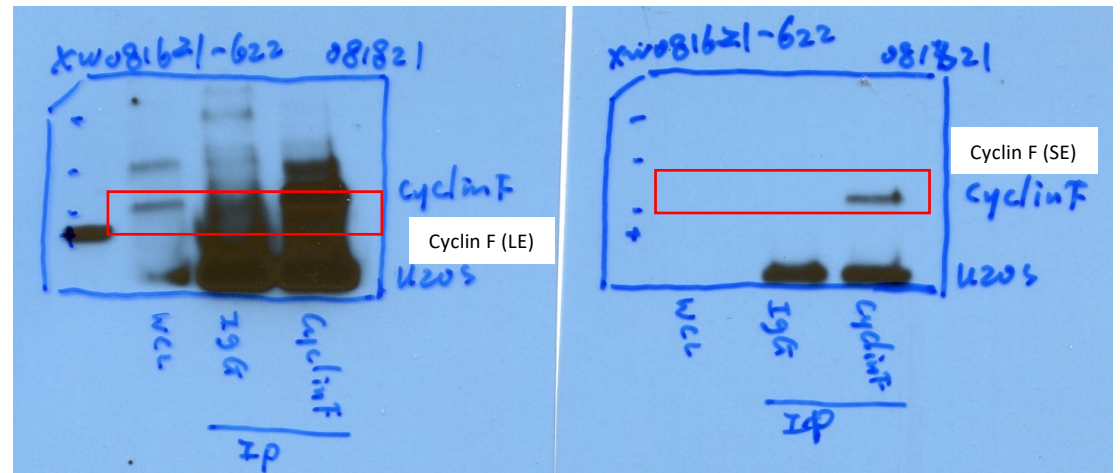

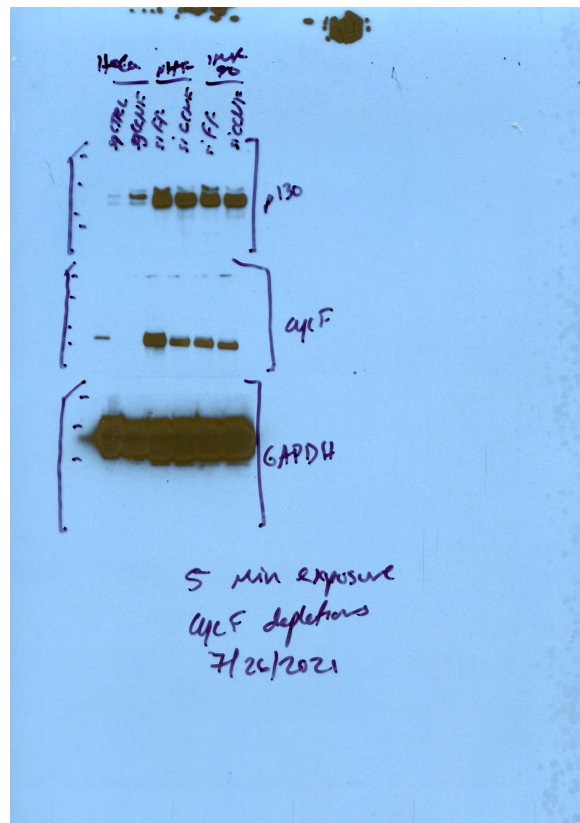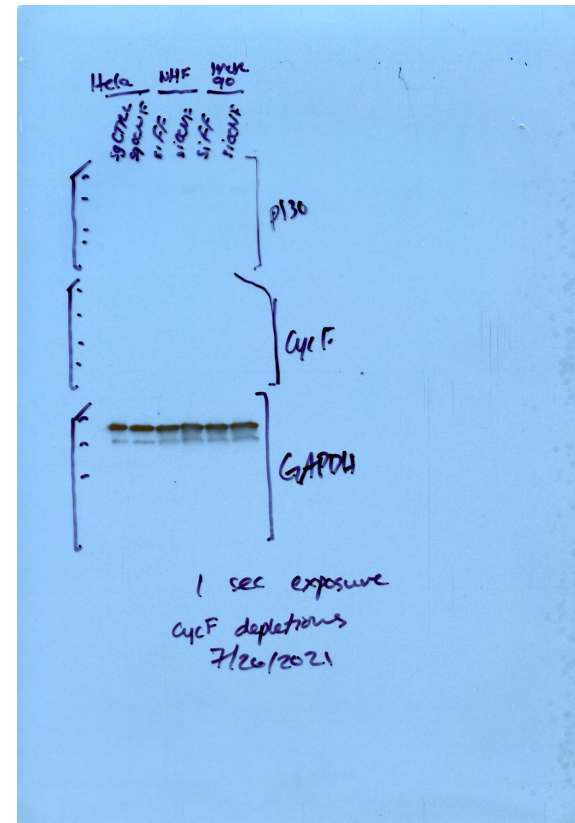

For the NHF-1 cells:

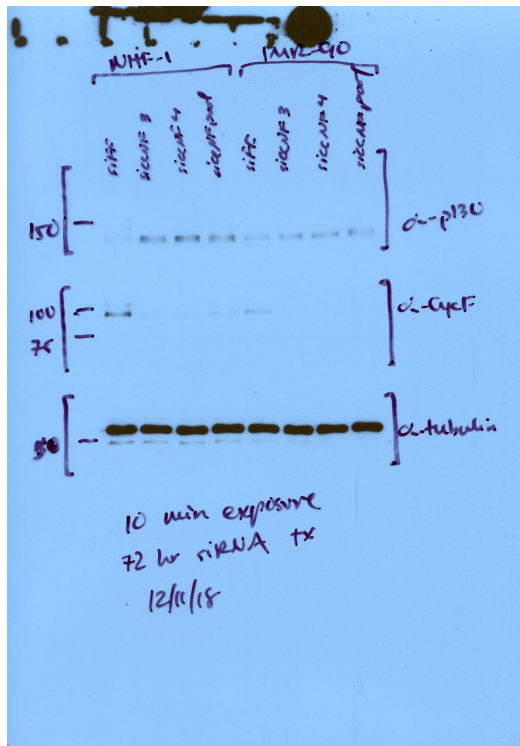

For the IMR-90 cells:

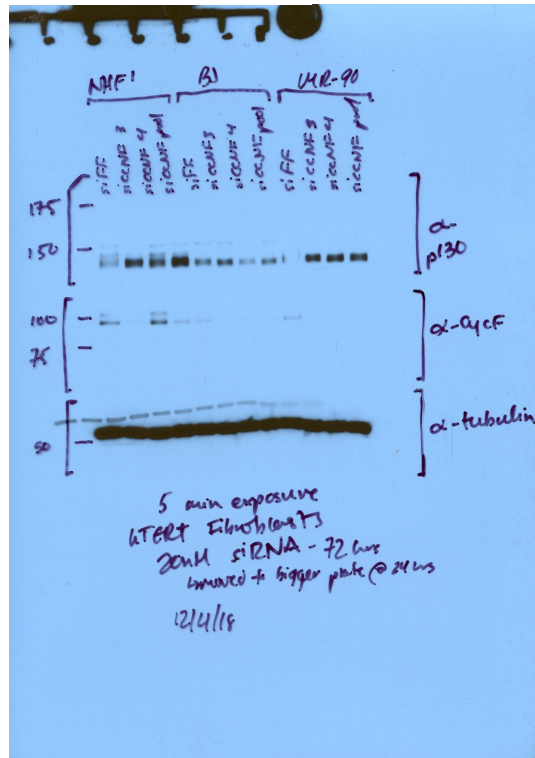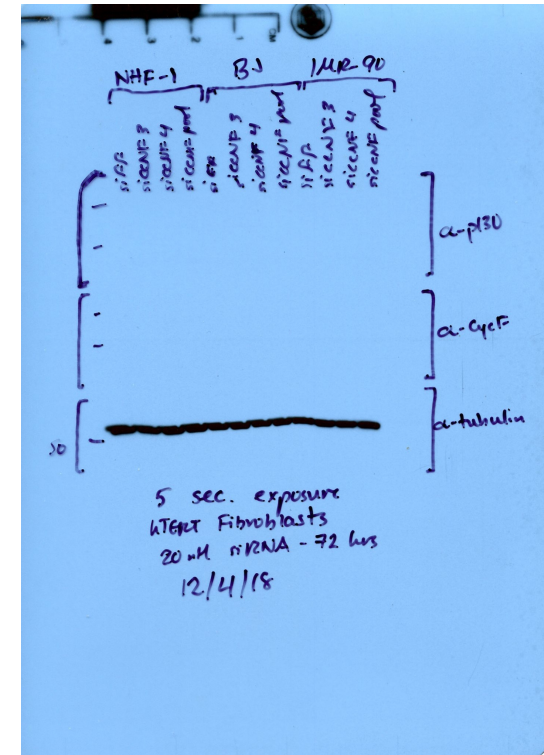

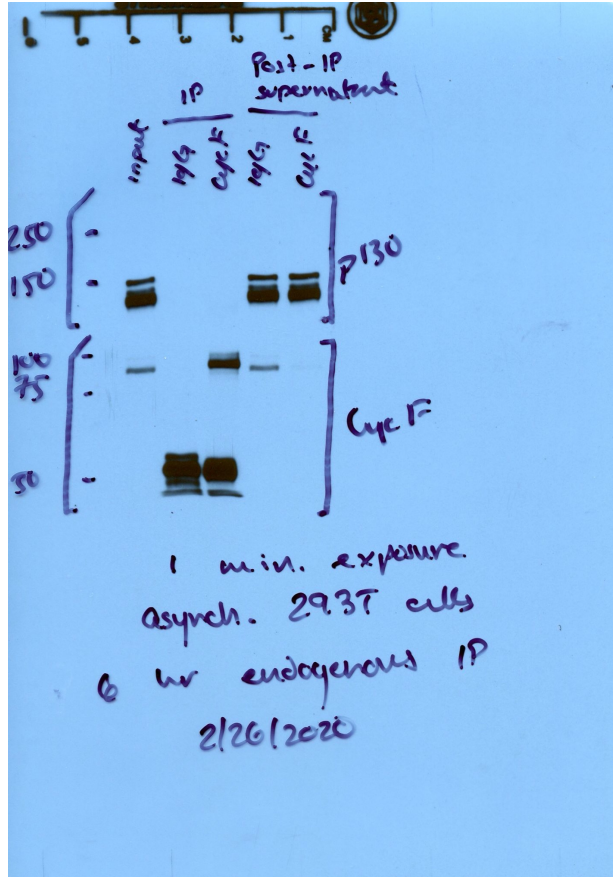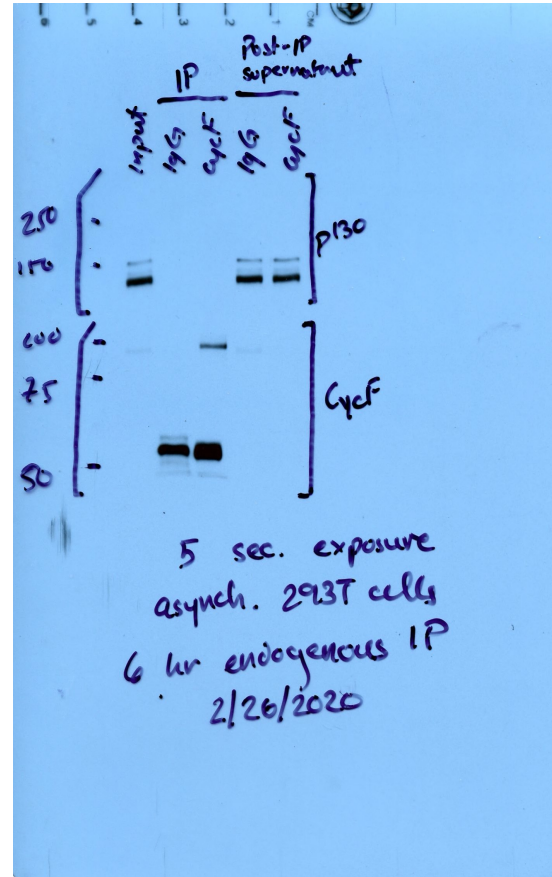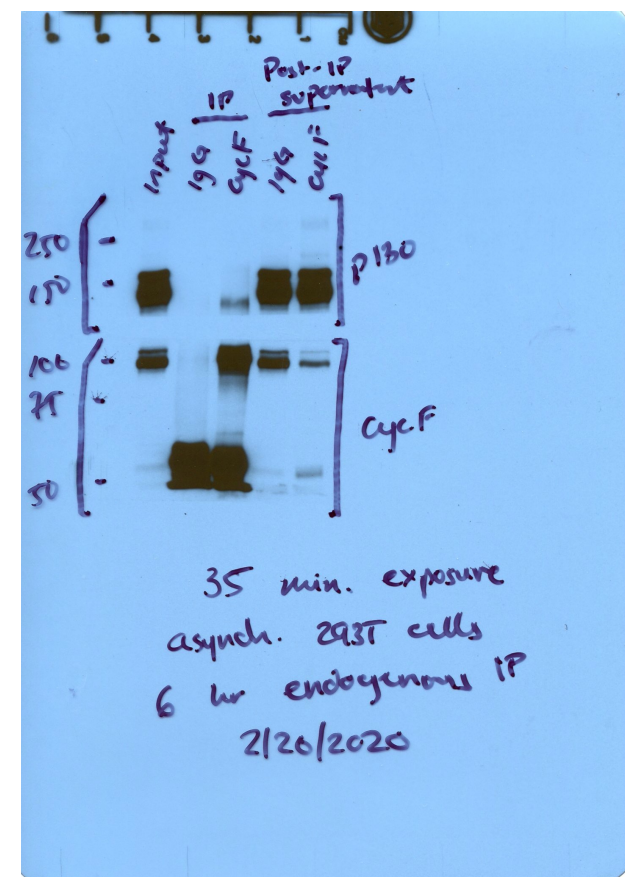

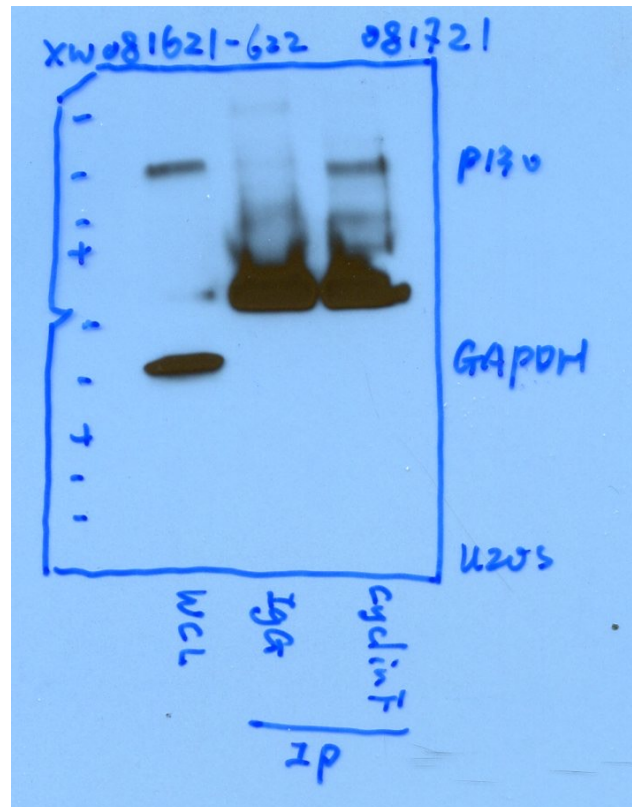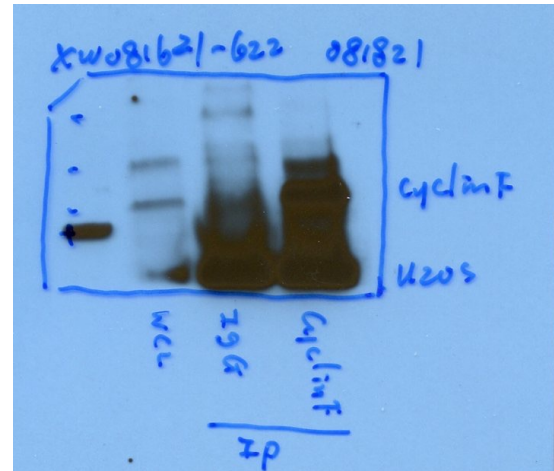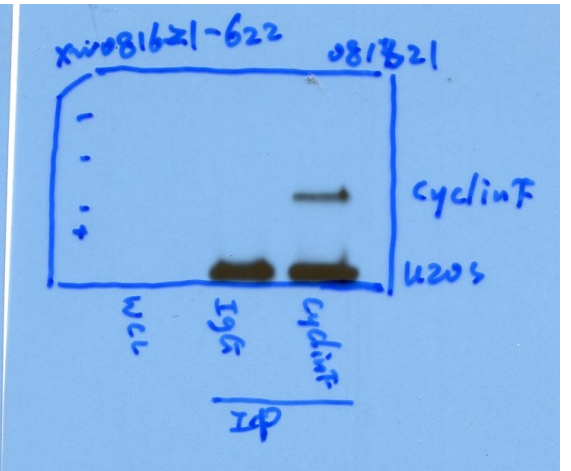

Supplement: Source data 1. — This source data file includes all uncropped blots used to generate data for the main figures and figure supplements. Additionally, copies of the uncropped images are shown a second time where blot strips shown in figures are highlighted with a red square and the protein that was blotted for is noted. [file elife-70691-supp2.zip › Source Data 1/Enrico-Fig2-source-data.pdf]

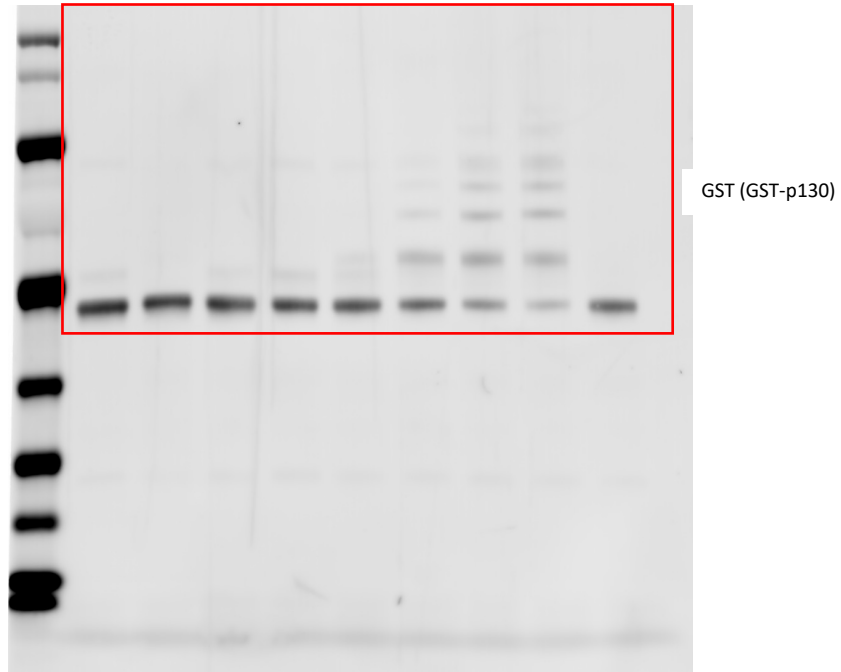

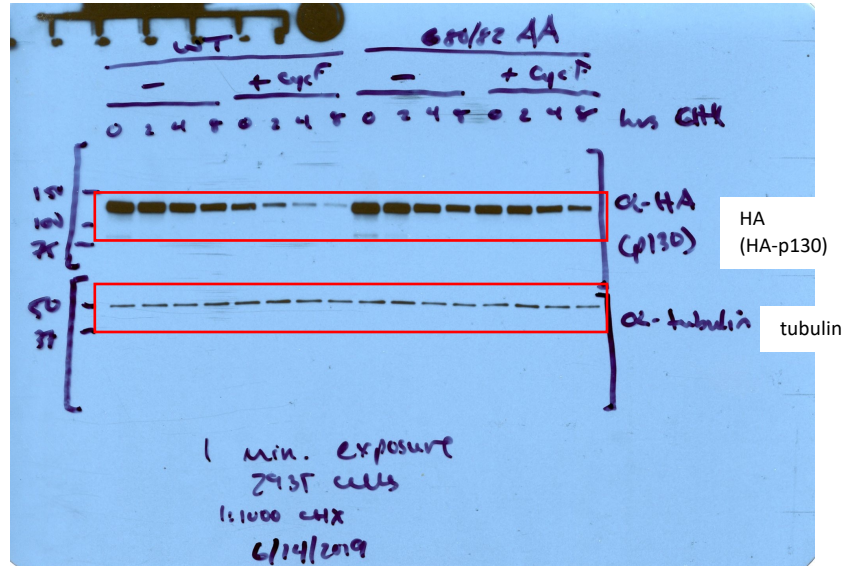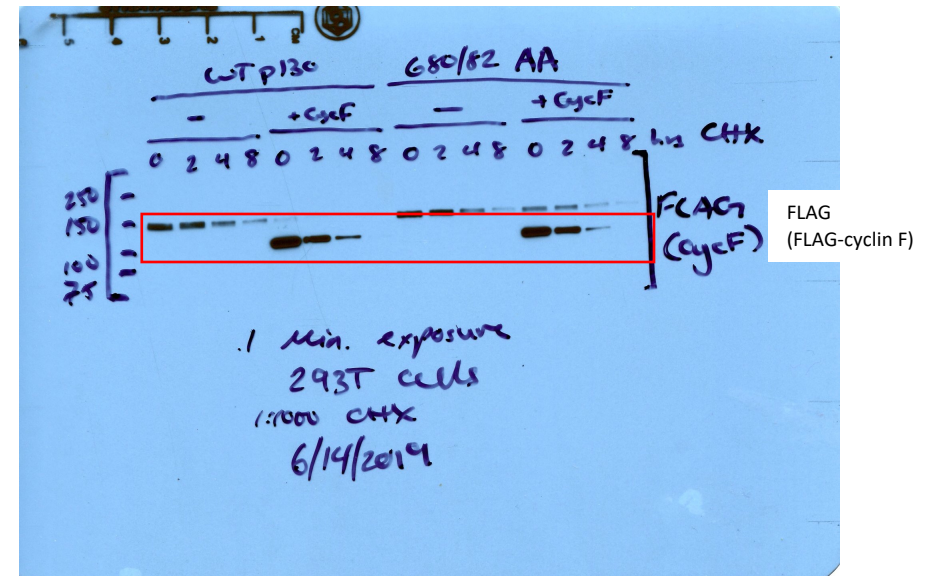

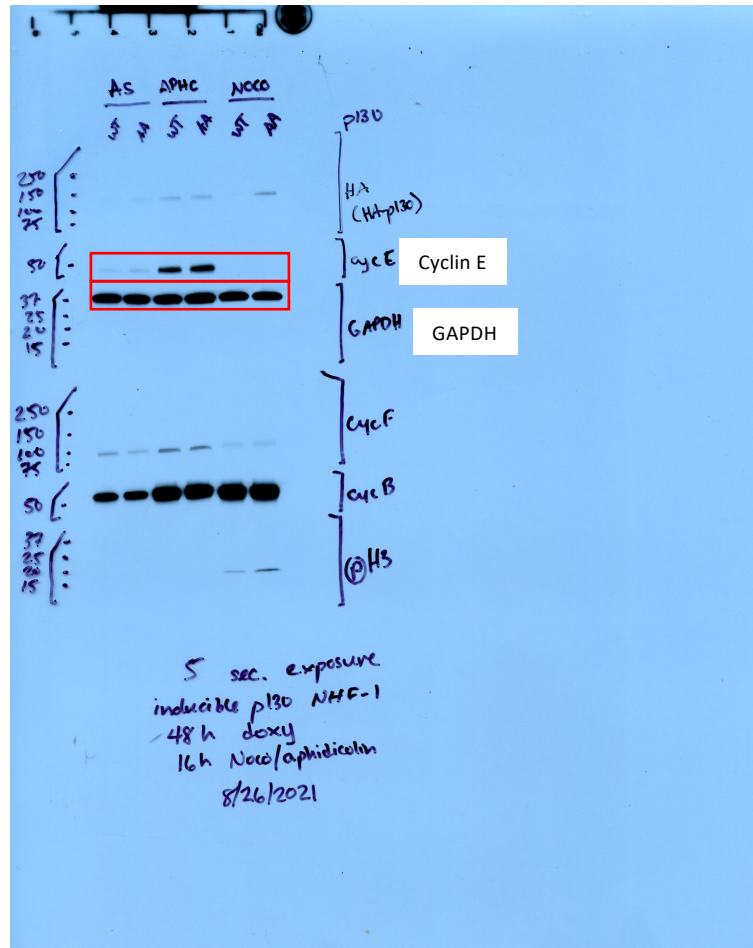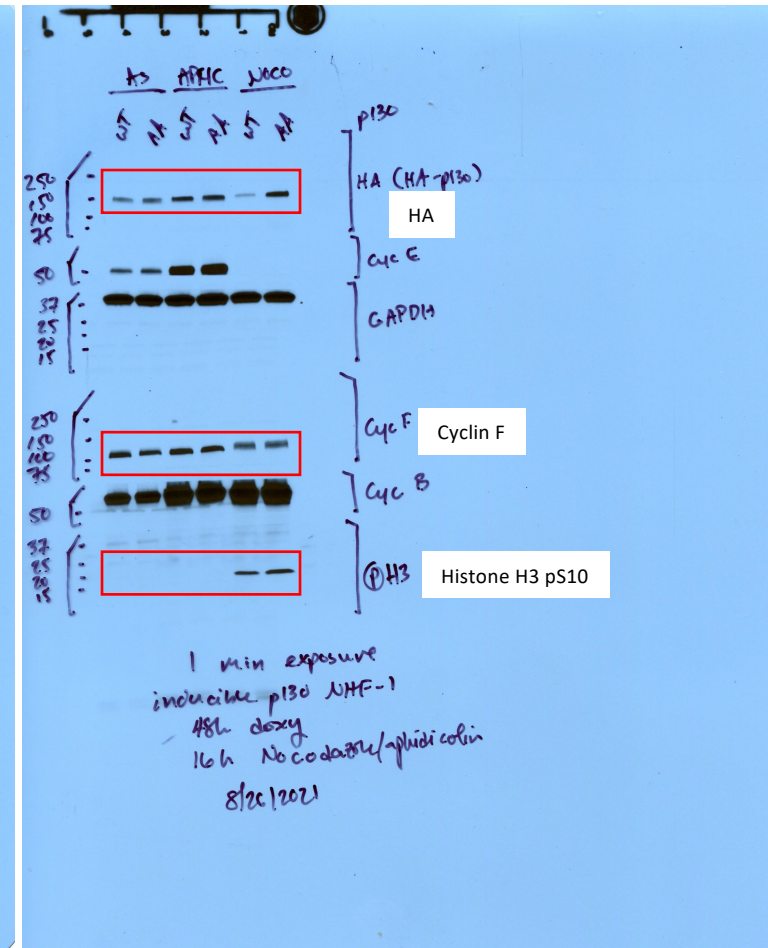

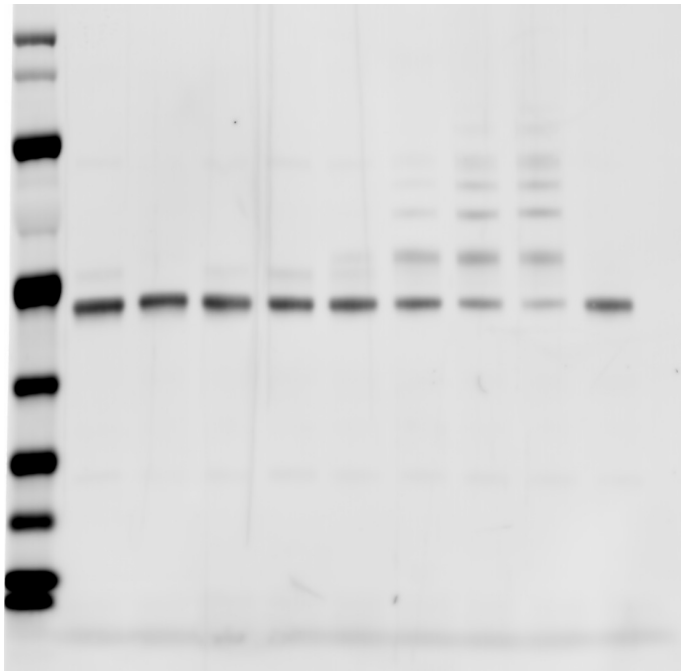

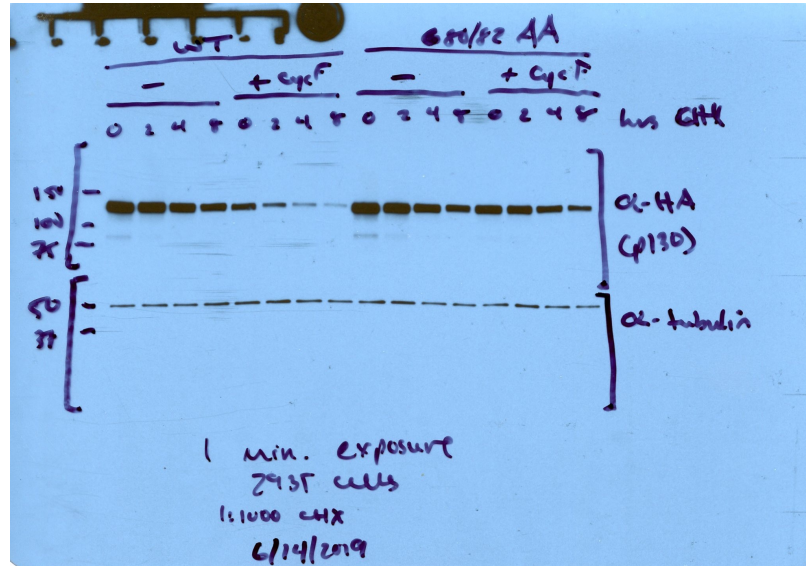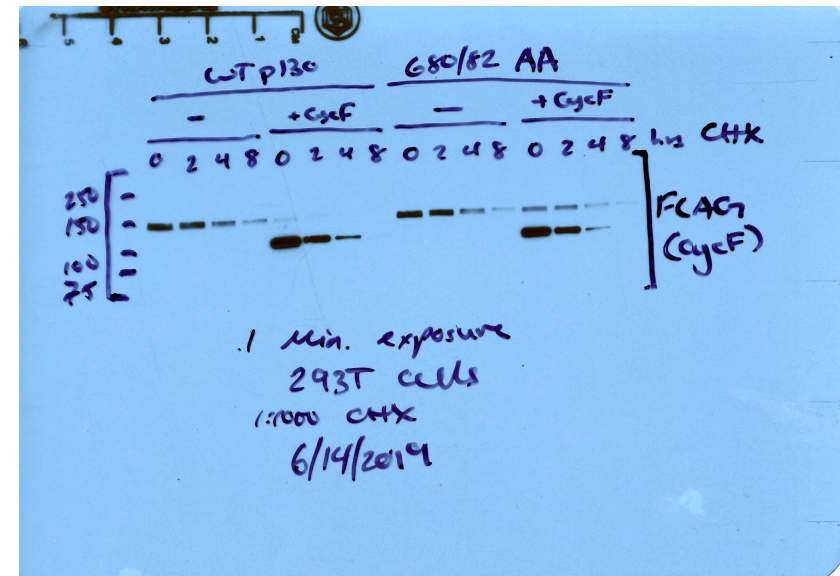

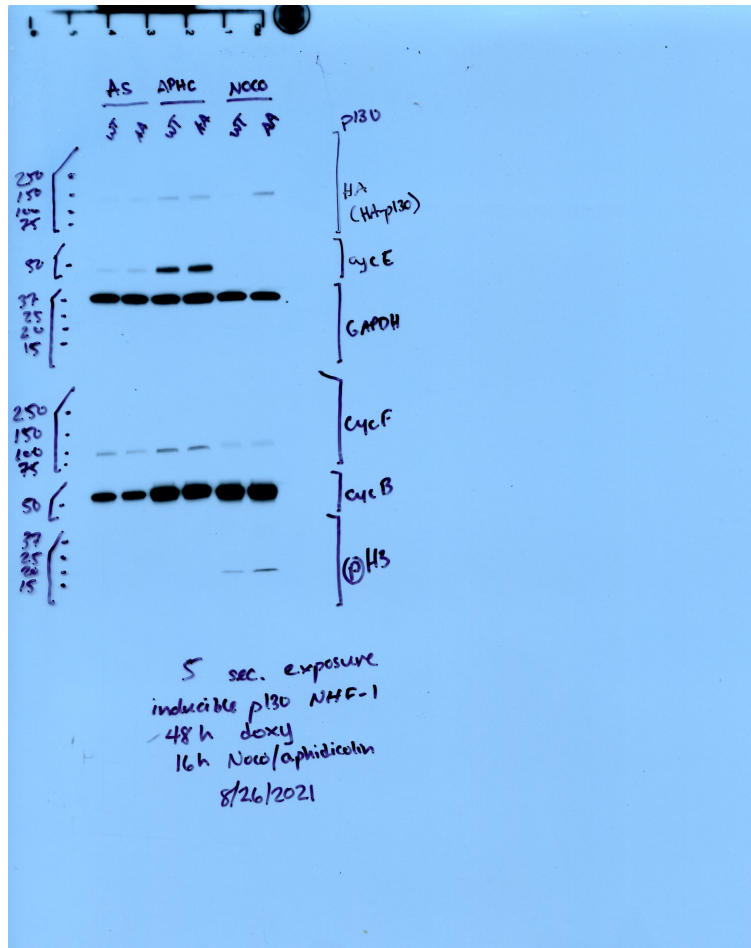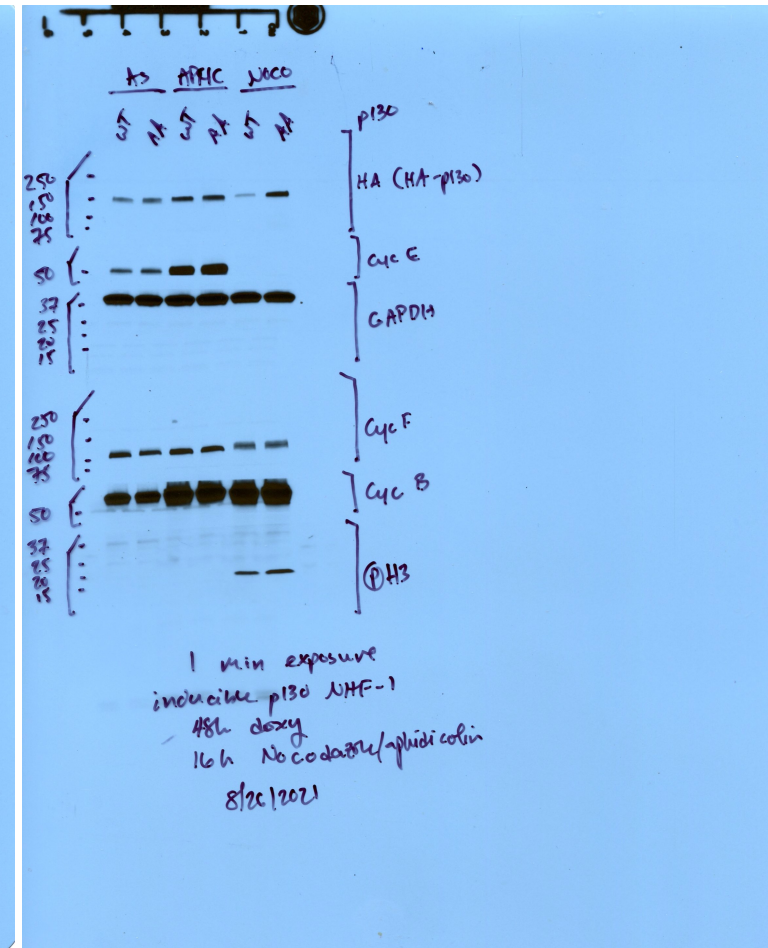

Supplement: Source data 1. — This source data file includes all uncropped blots used to generate data for the main figures and figure supplements. Additionally, copies of the uncropped images are shown a second time where blot strips shown in figures are highlighted with a red square and the protein that was blotted for is noted. [file elife-70691-supp2.zip › Source Data 1/Enrico-Fig5-source-data.pdf]

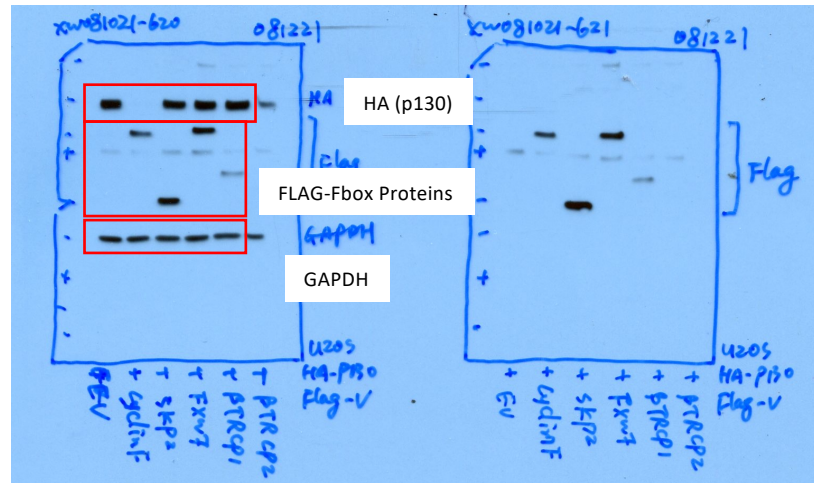

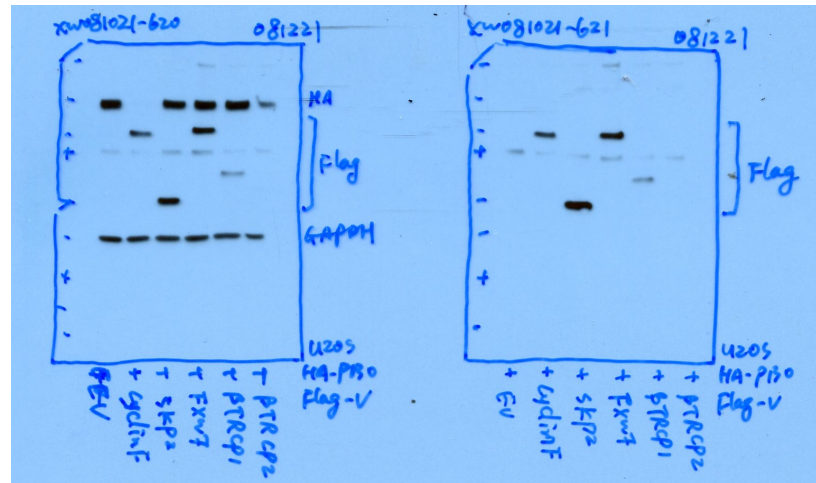

Supplement: Source data 1. — This source data file includes all uncropped blots used to generate data for the main figures and figure supplements. Additionally, copies of the uncropped images are shown a second time where blot strips shown in figures are highlighted with a red square and the protein that was blotted for is noted. [file elife-70691-supp2.zip › Source Data 1/Enrico-Fig3S1-source-data.pdf]

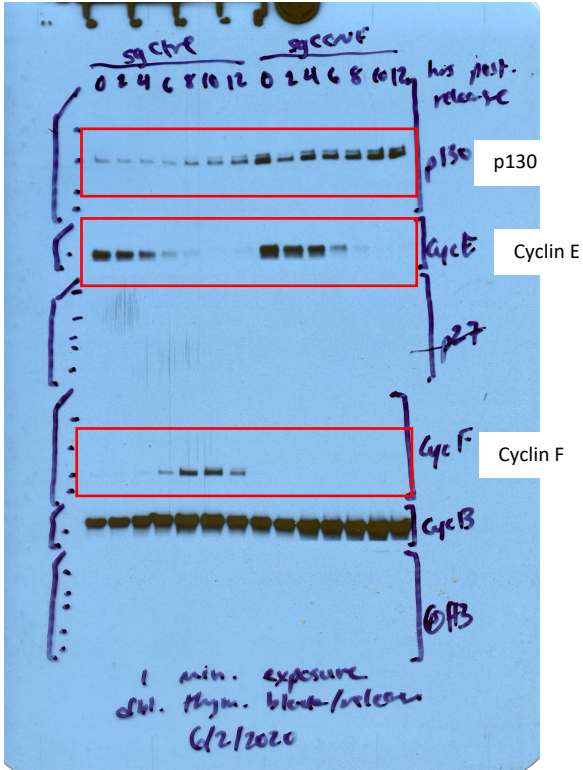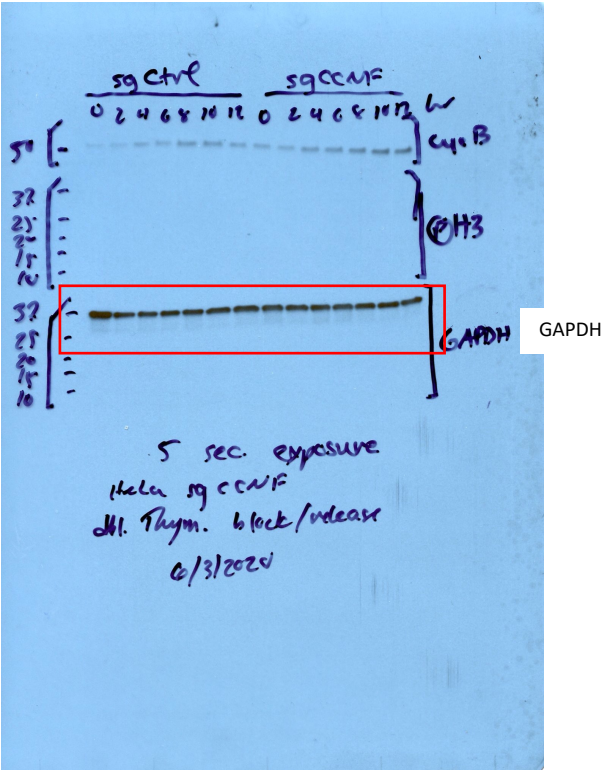

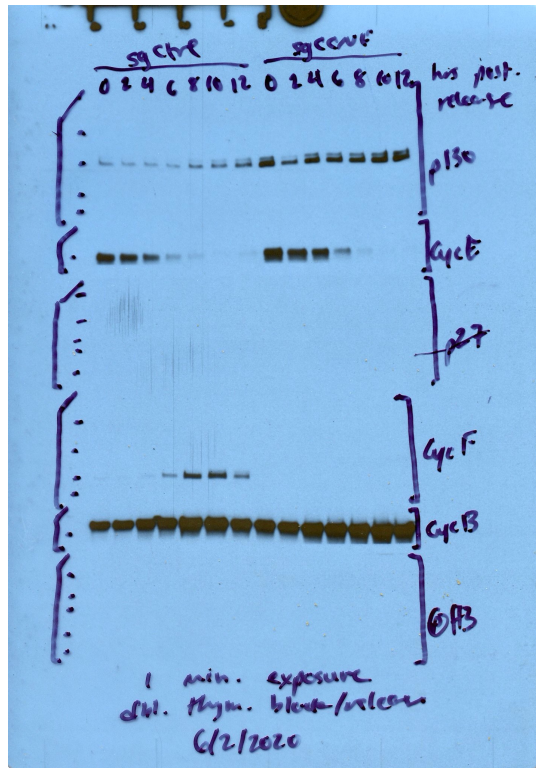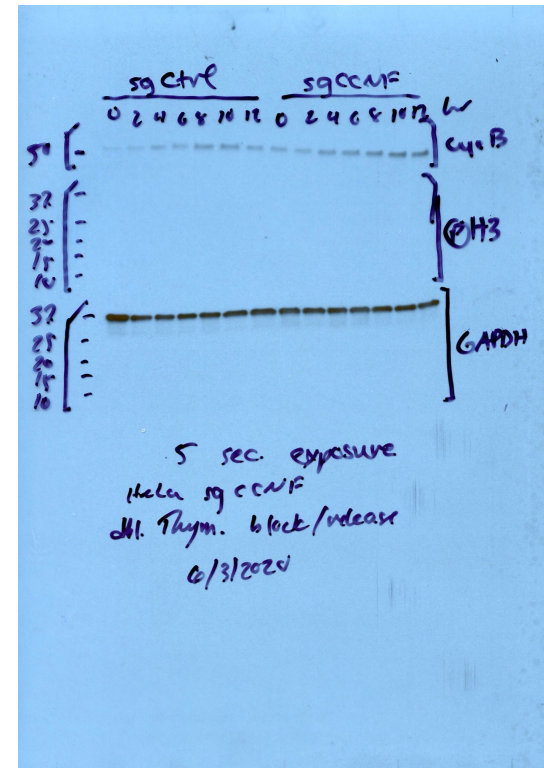

Supplement: Source data 1. — This source data file includes all uncropped blots used to generate data for the main figures and figure supplements. Additionally, copies of the uncropped images are shown a second time where blot strips shown in figures are highlighted with a red square and the protein that was blotted for is noted. [file elife-70691-supp2.zip › Source Data 1/Enrico-Fig2S1-source-data.pdf]

# Enrico\_Fig4 S1\_Source Data 1 For Fig4 S1A

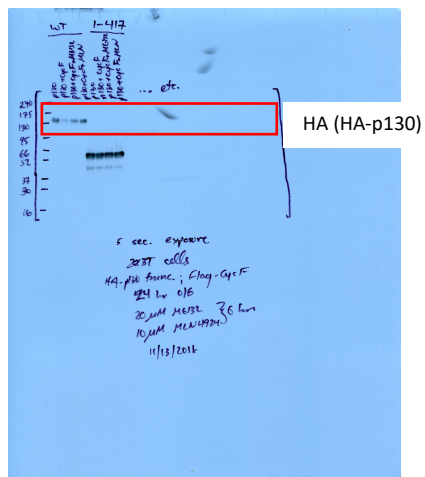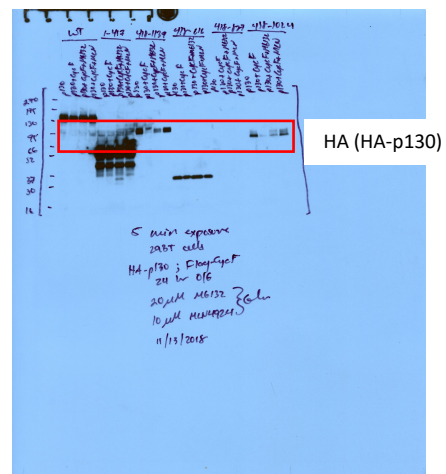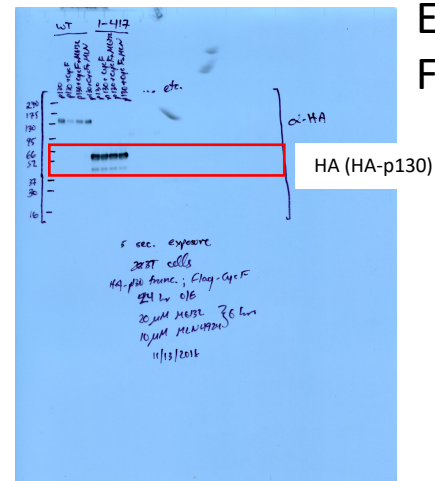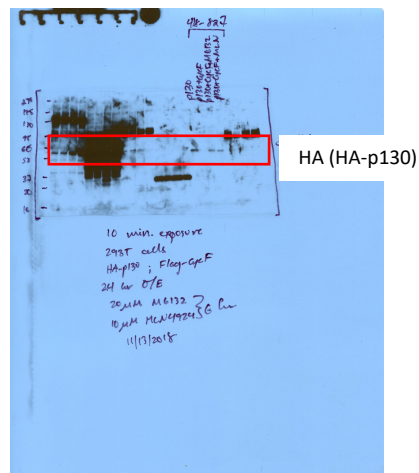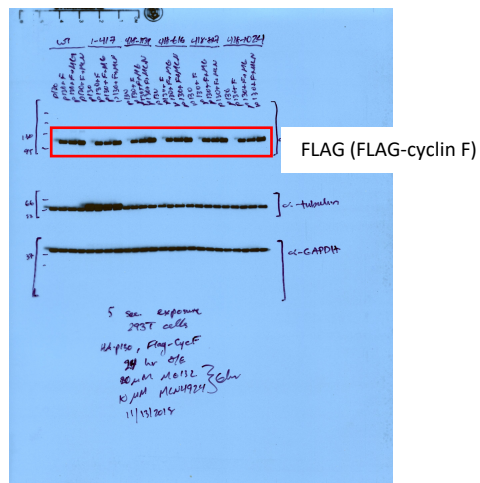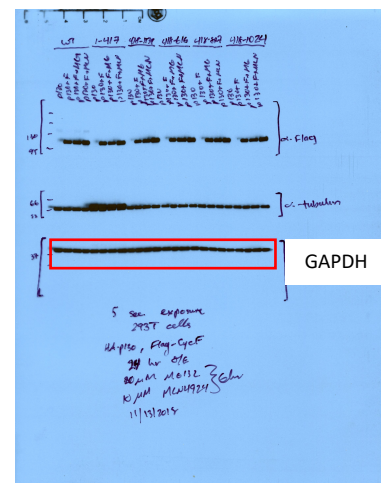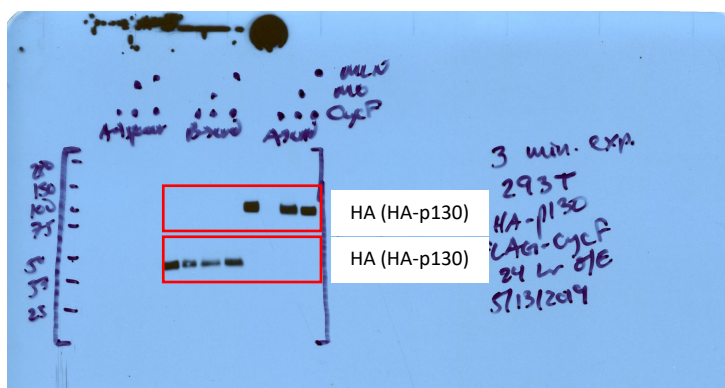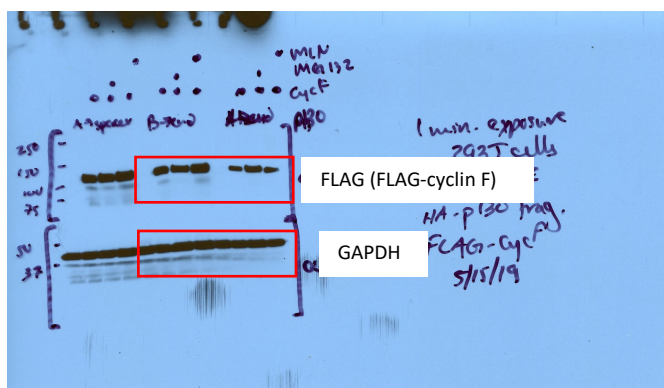

# Enrico\_Fig4 S1\_Source Data 2 For Fig4 S1A

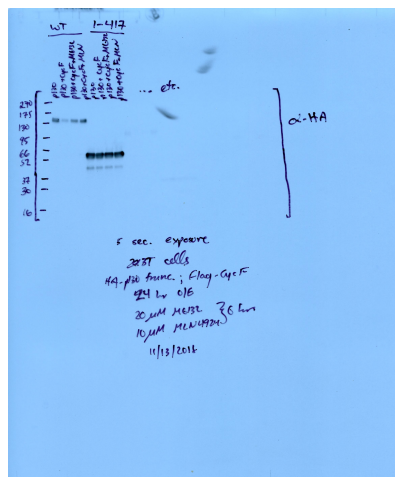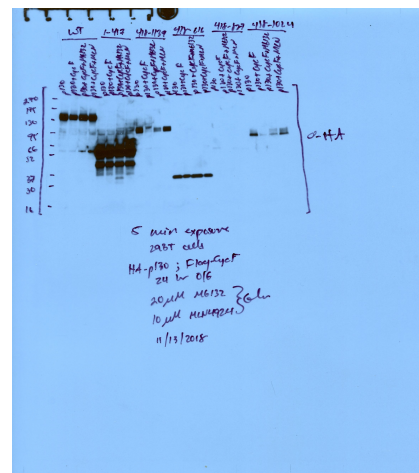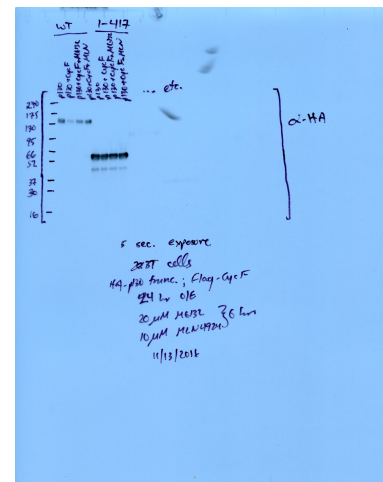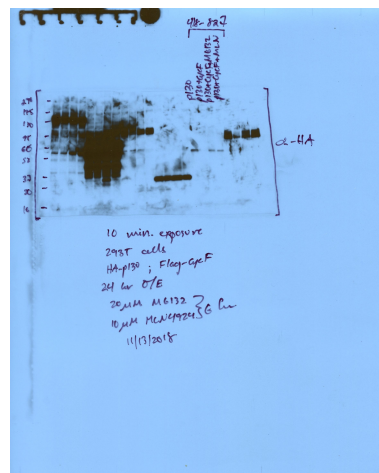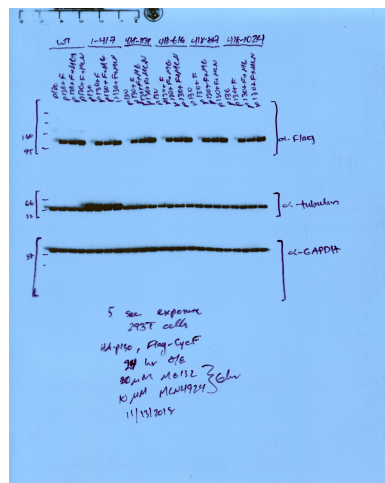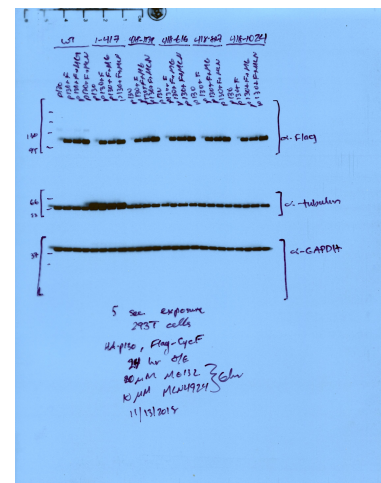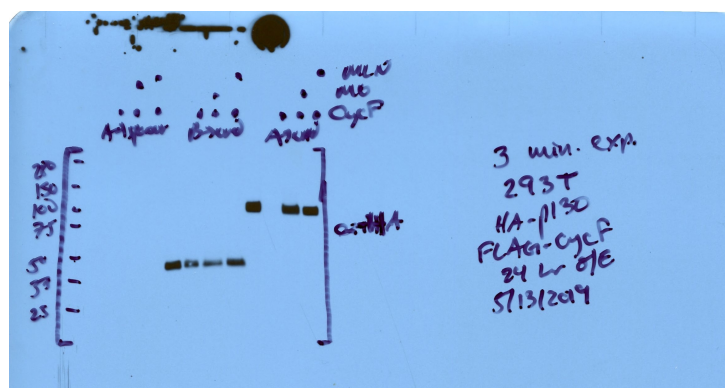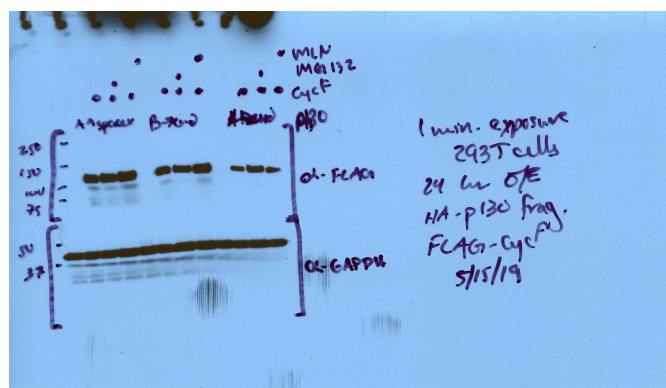

Supplement: Source data 1. — This source data file includes all uncropped blots used to generate data for the main figures and figure supplements. Additionally, copies of the uncropped images are shown a second time where blot strips shown in figures are highlighted with a red square and the protein that was blotted for is noted. [file elife-70691-supp2.zip › Source Data 1/Enrico-Fig4S1-source-data.pdf]

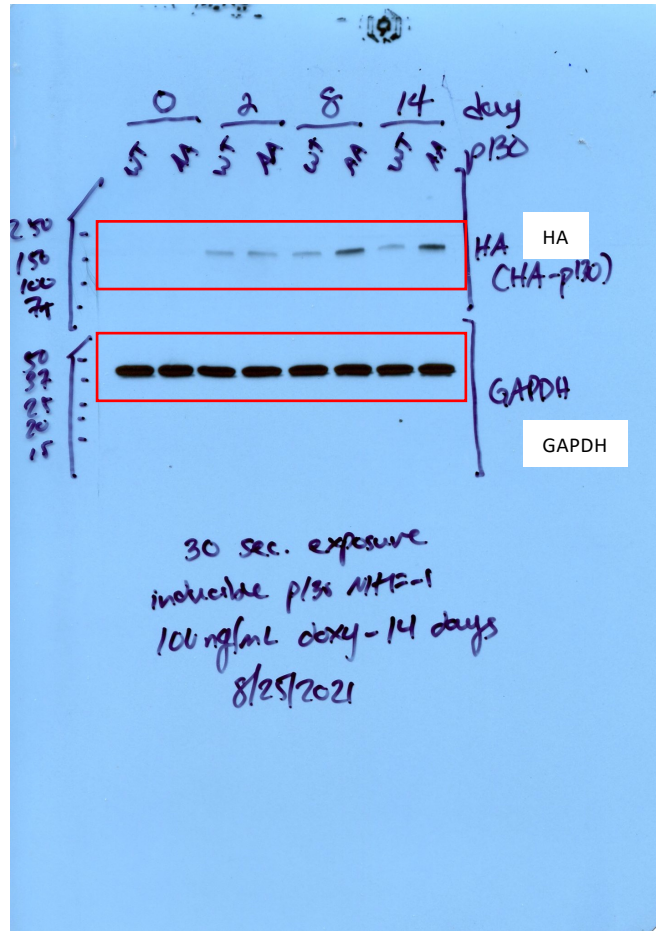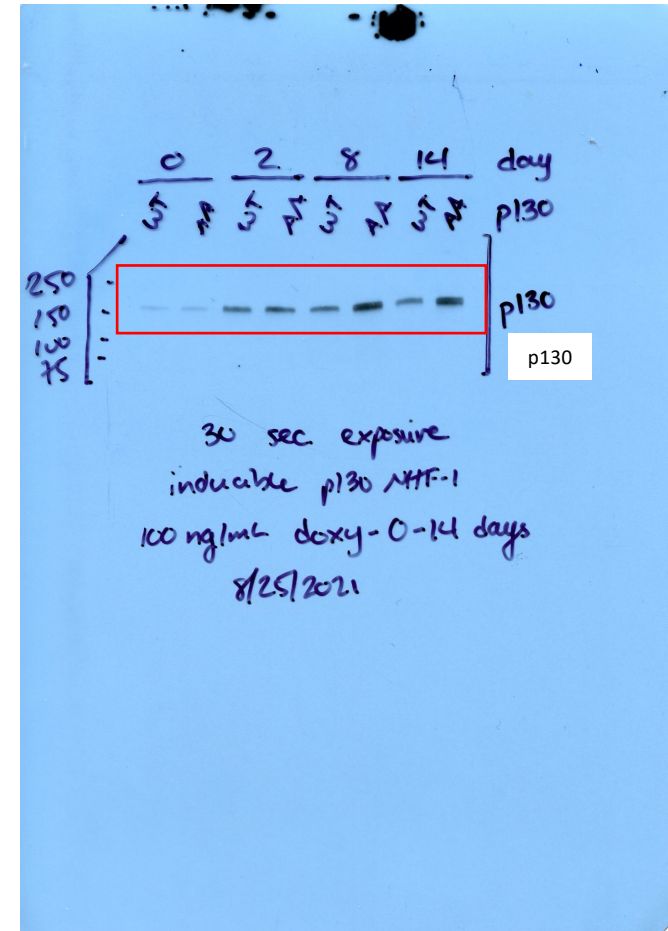

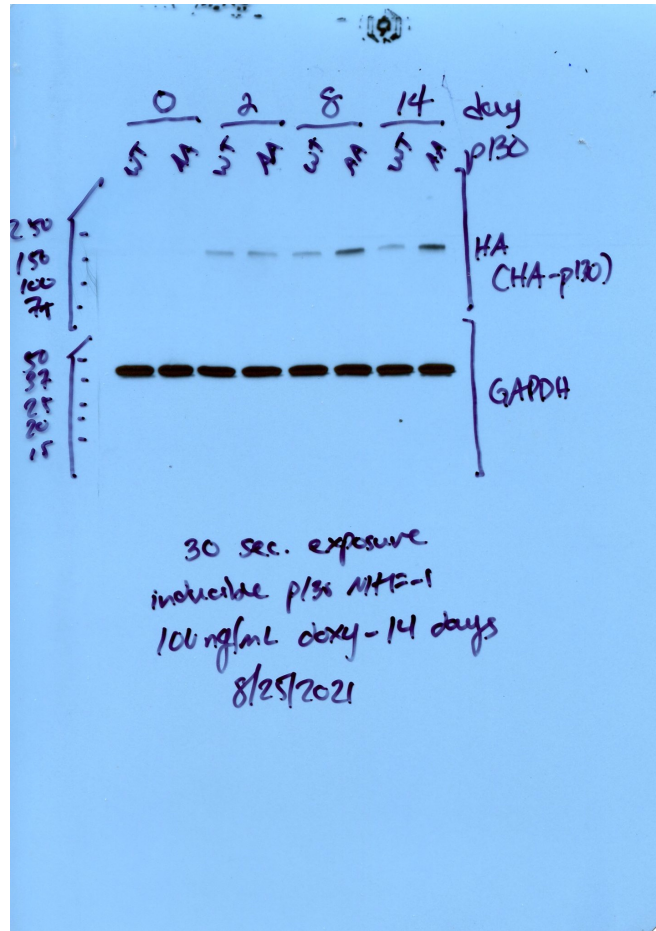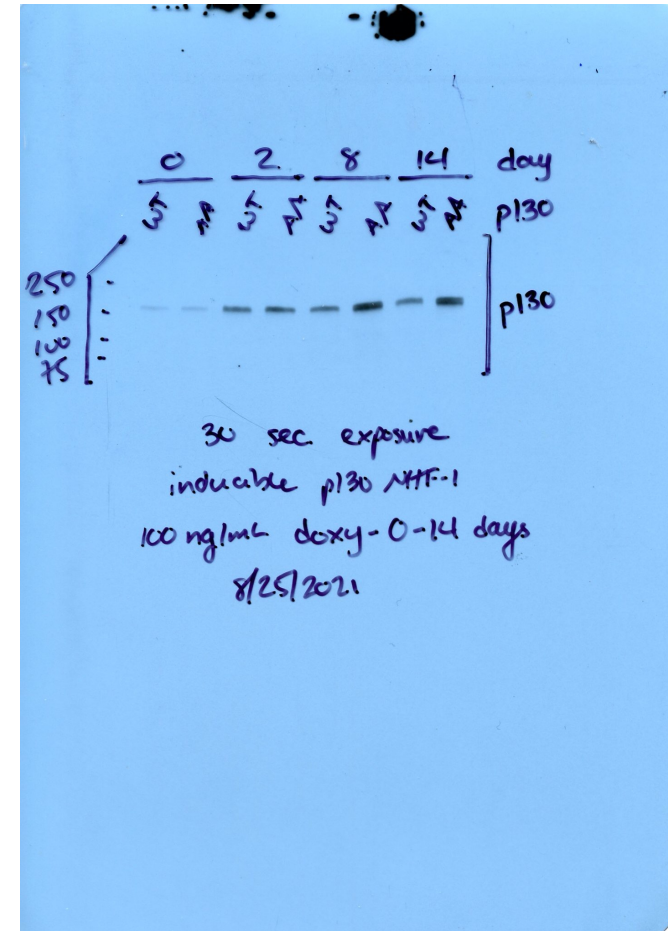

Supplement: Source data 1. — This source data file includes all uncropped blots used to generate data for the main figures and figure supplements. Additionally, copies of the uncropped images are shown a second time where blot strips shown in figures are highlighted with a red square and the protein that was blotted for is noted. [file elife-70691-supp2.zip › Source Data 1/Enrico-Fig6-source-data.pdf]

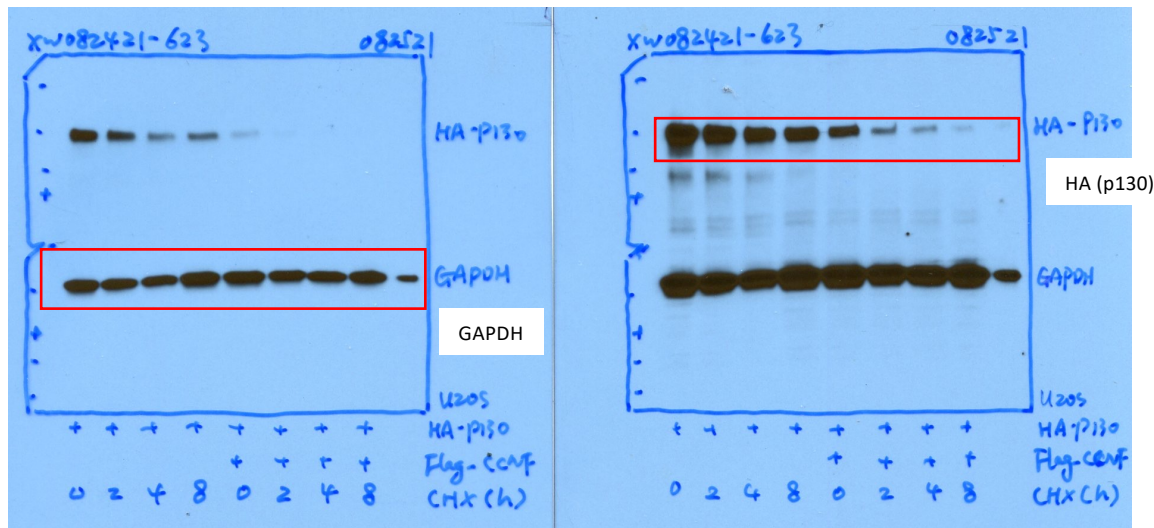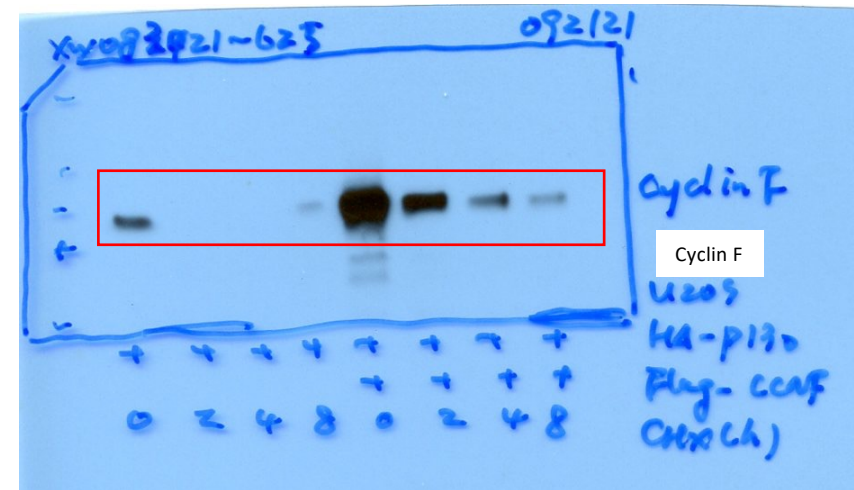

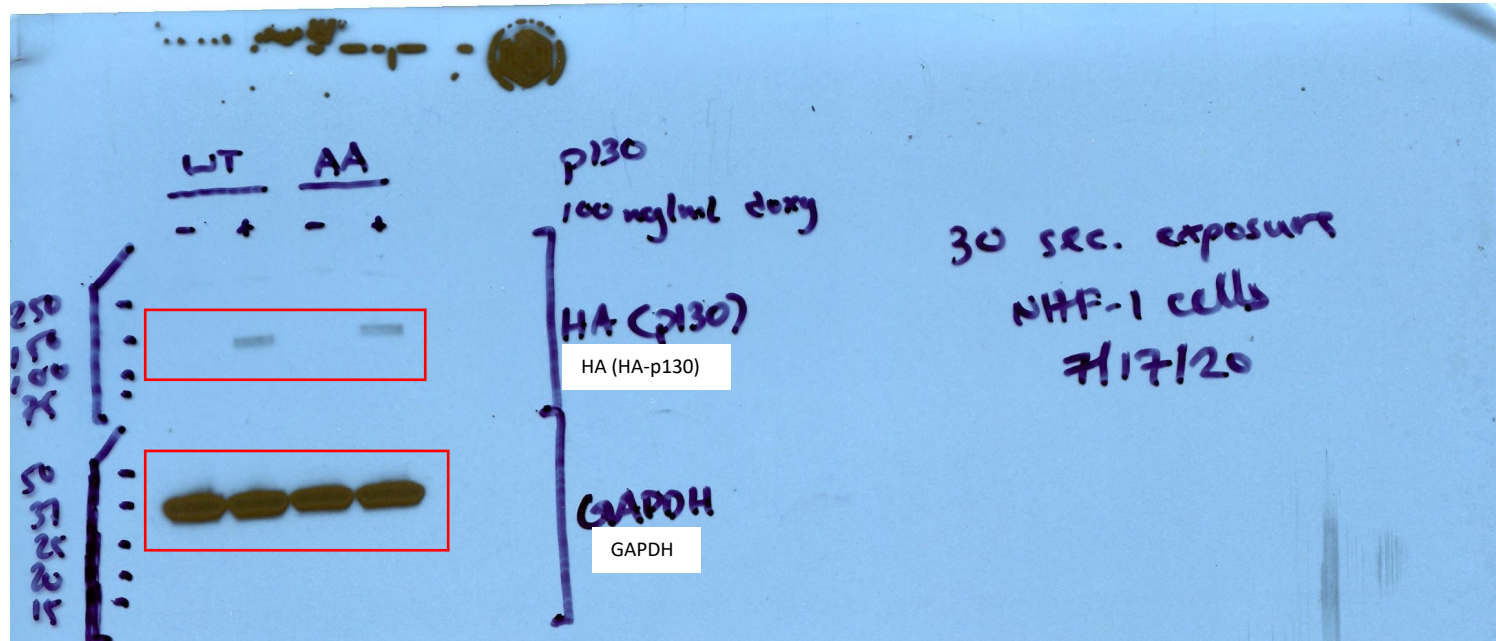

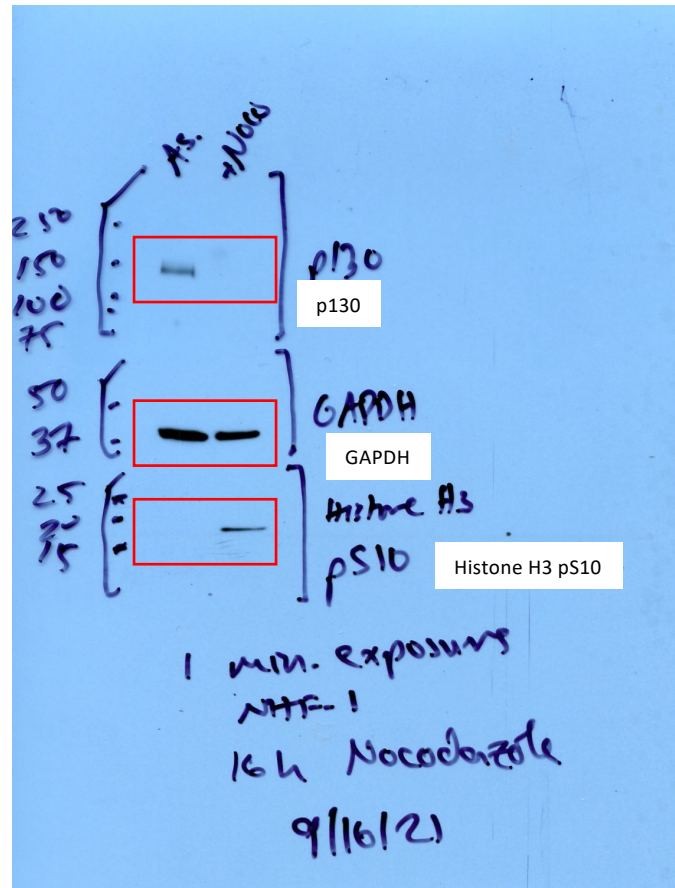

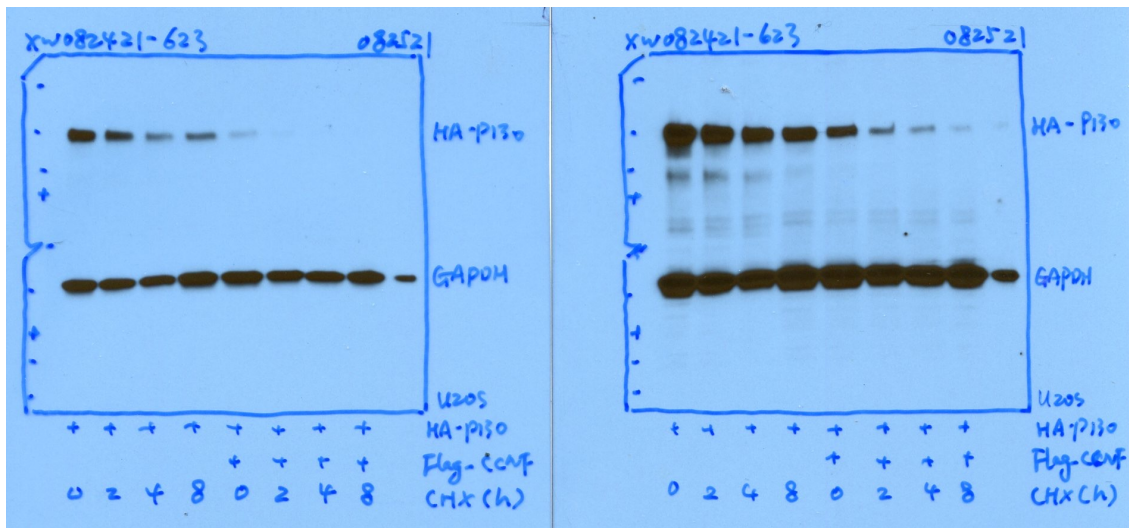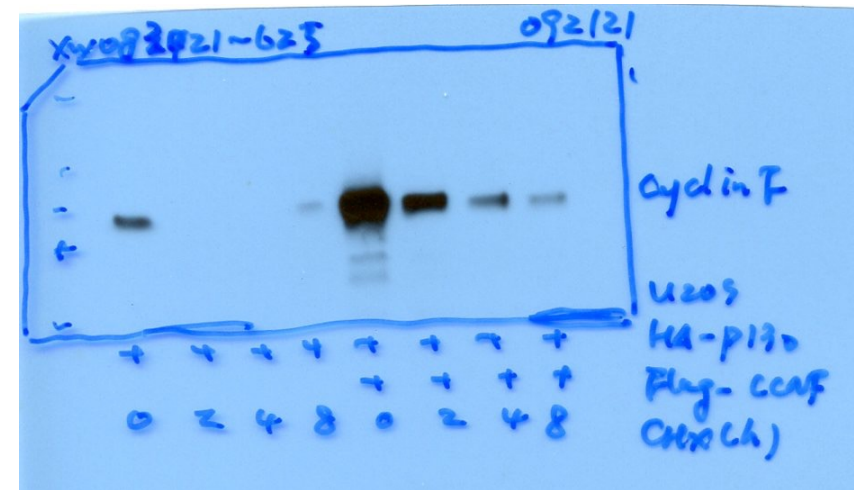

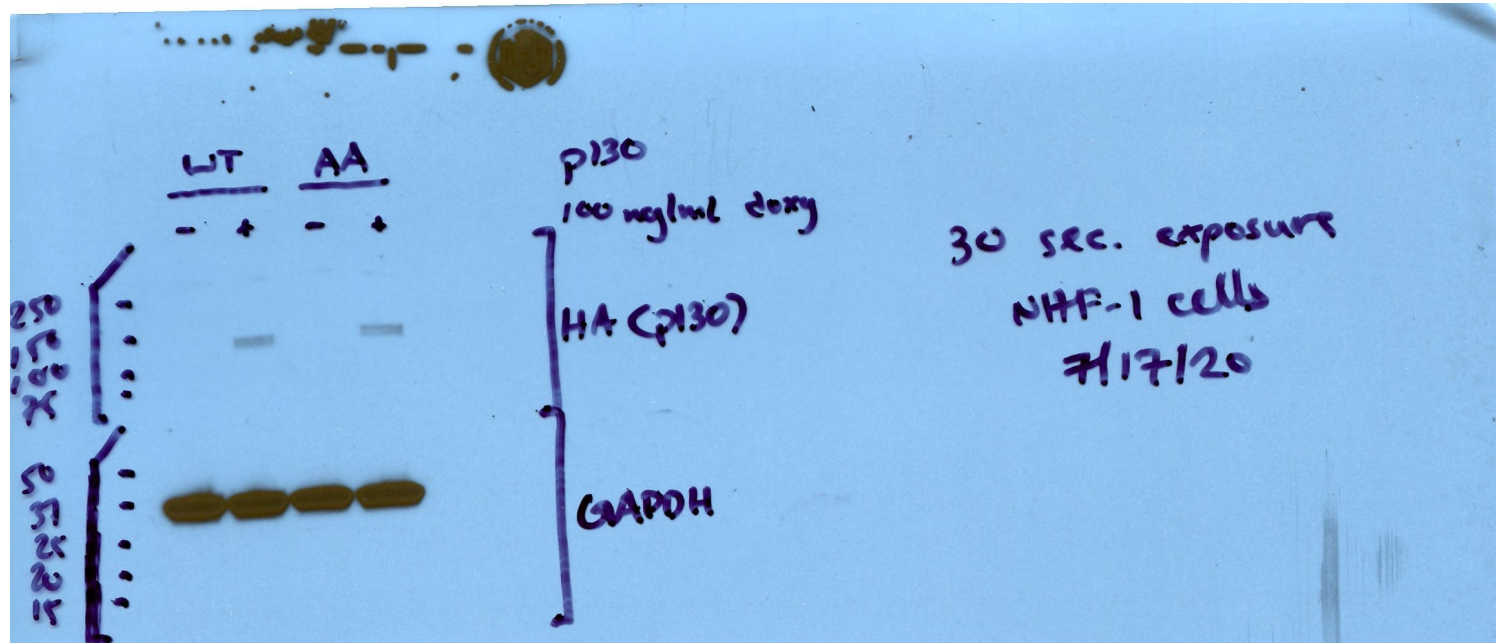

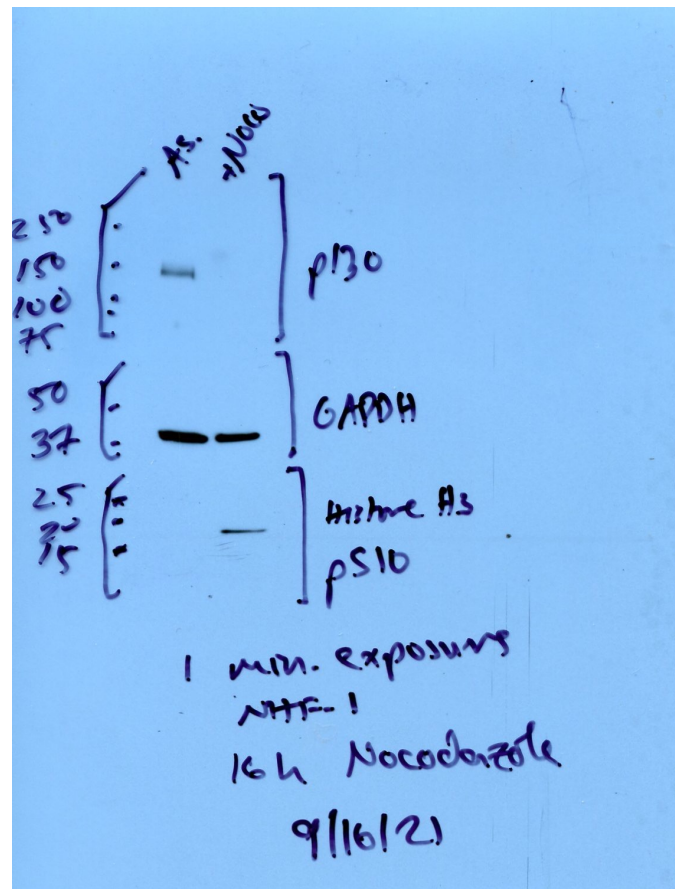

Supplement: Source data 1. — This source data file includes all uncropped blots used to generate data for the main figures and figure supplements. Additionally, copies of the uncropped images are shown a second time where blot strips shown in figures are highlighted with a red square and the protein that was blotted for is noted. [file elife-70691-supp2.zip › Source Data 1/Enrico-Fig5S1-source-data.pdf]

# Enrico\_Fig1\_Source Data 1 For Fig1D

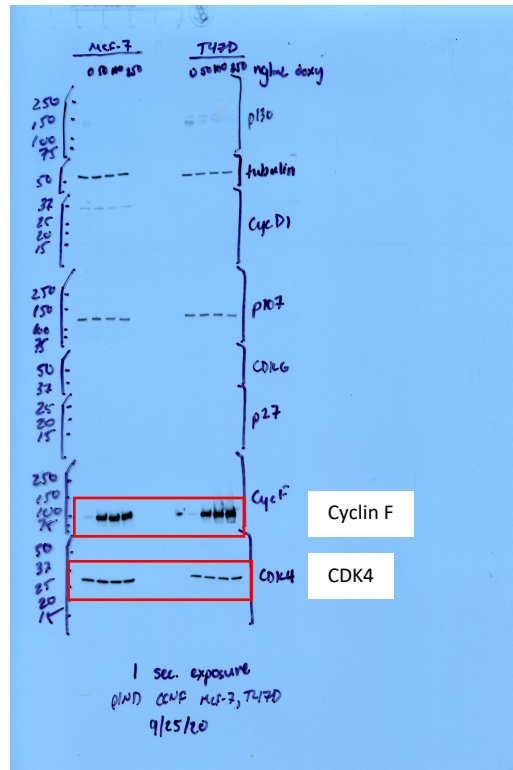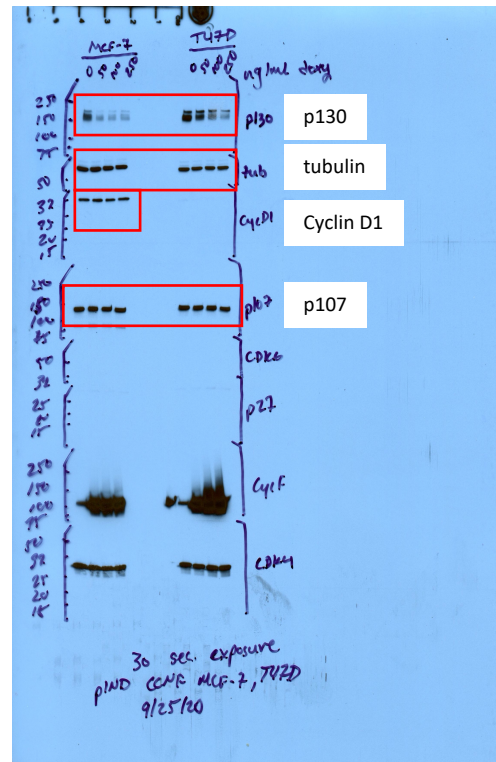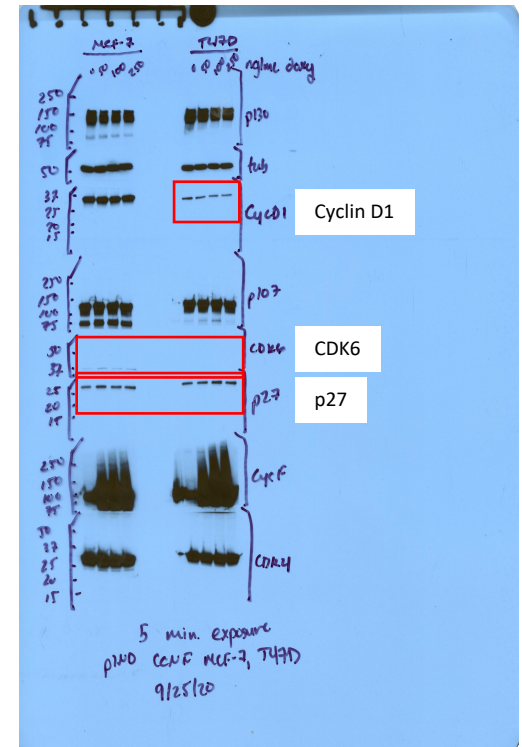

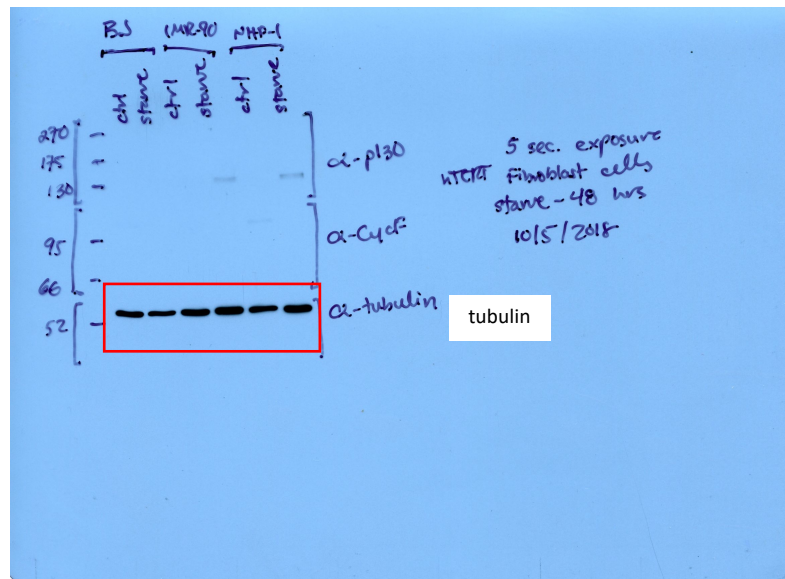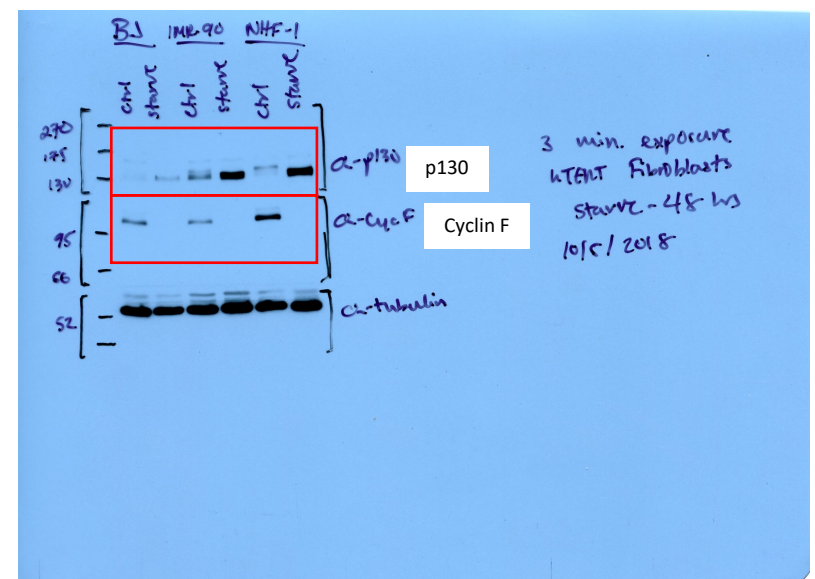

# Enrico\_Fig1\_Source Data 3 For Fig 1F

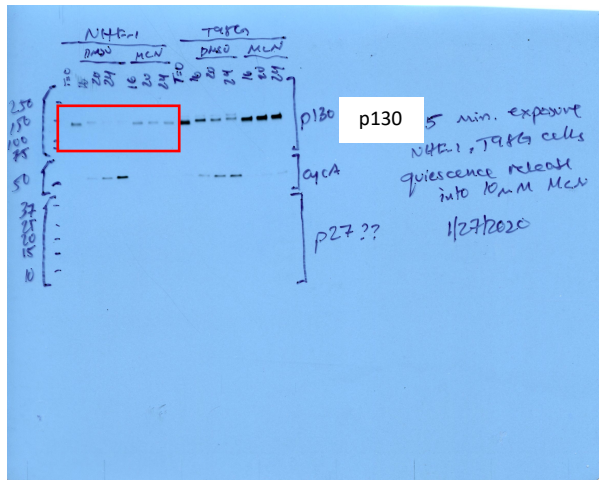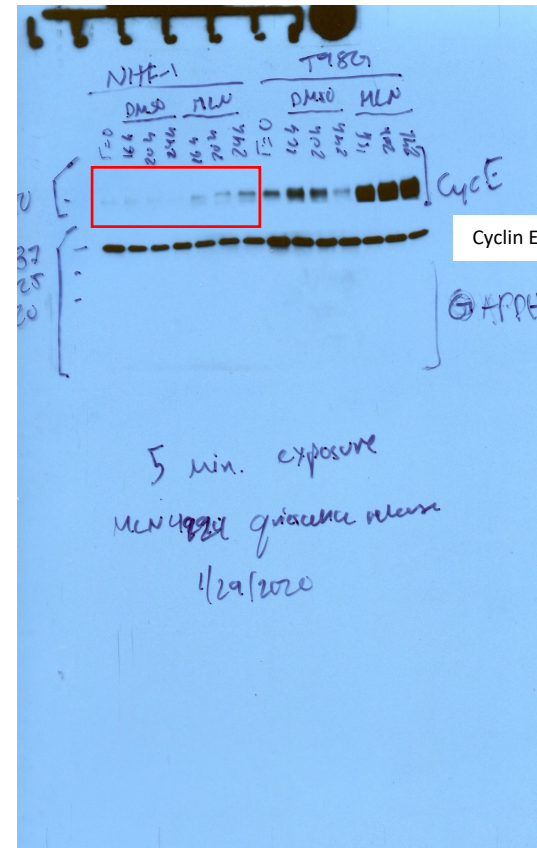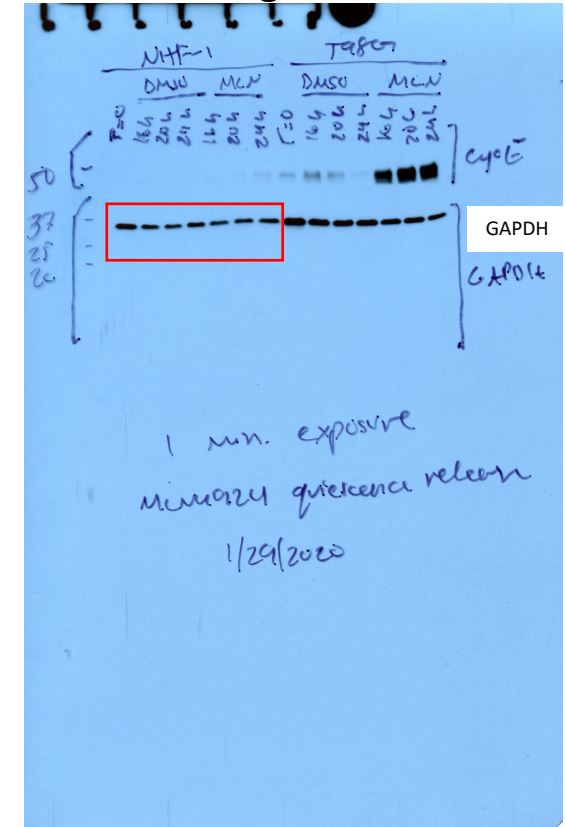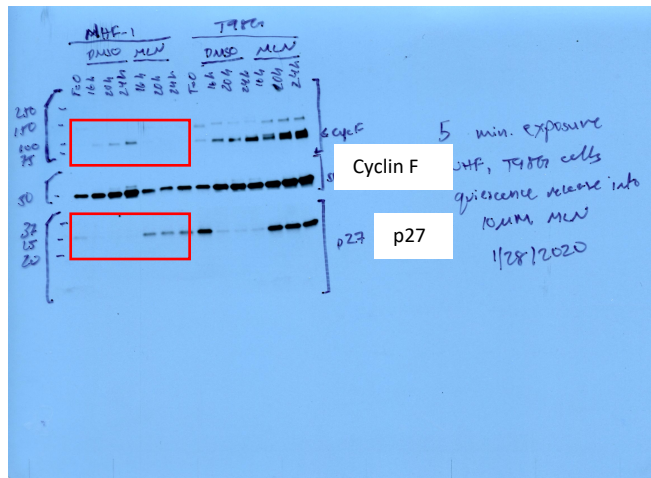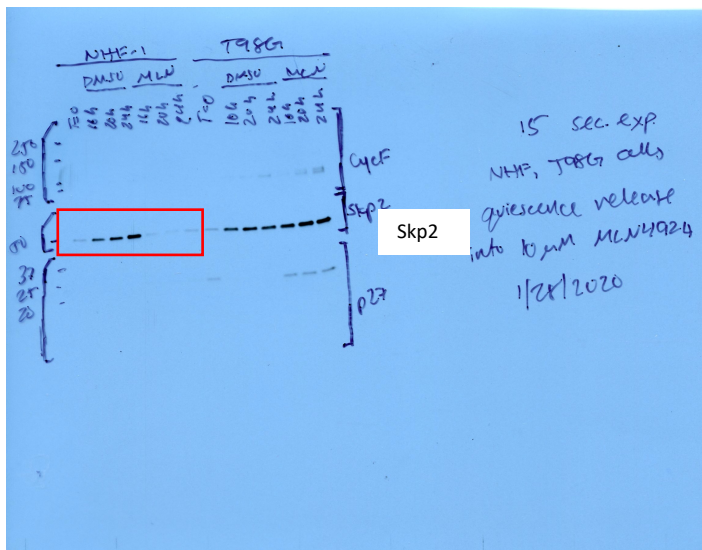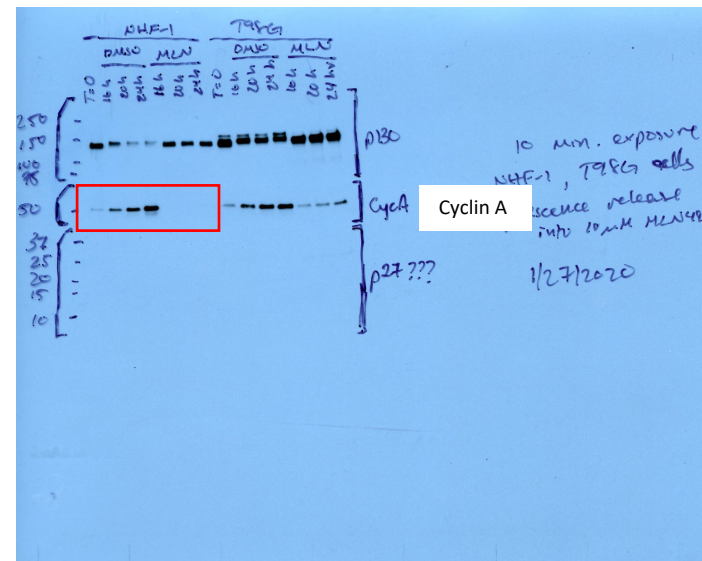

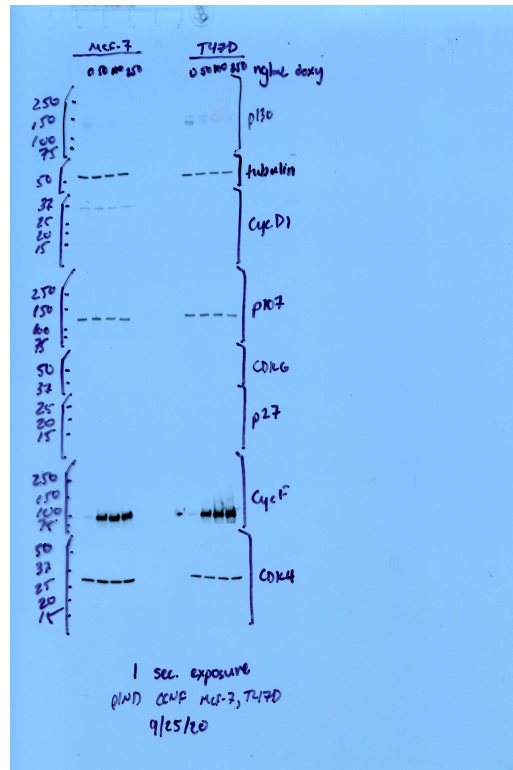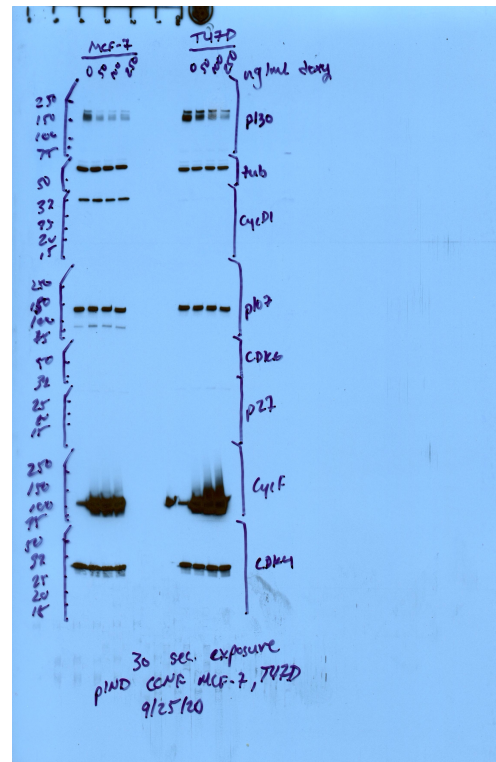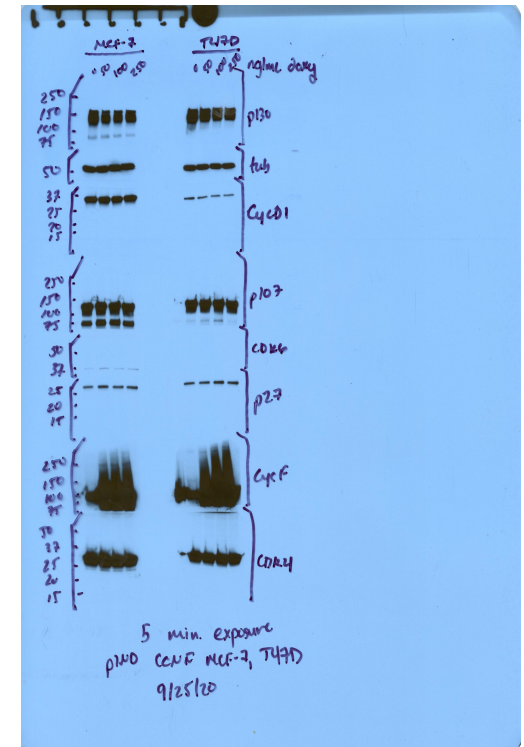

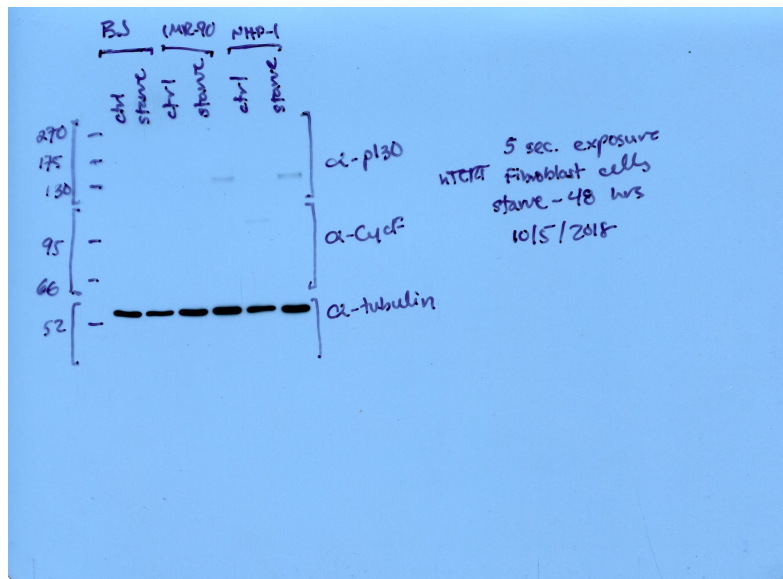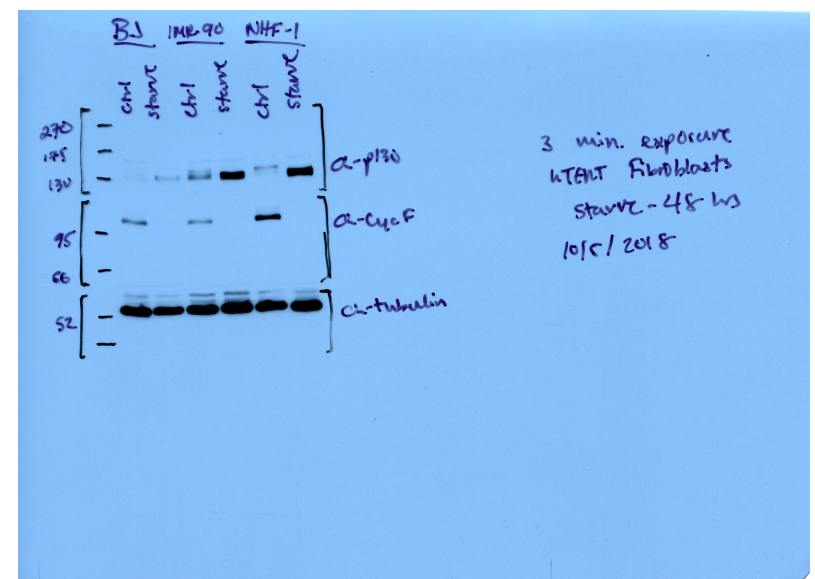

# Enrico\_Fig1\_Source Data 6 For Fig 1F

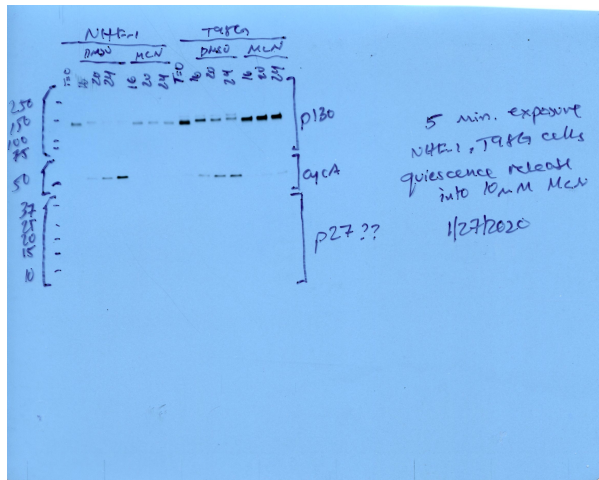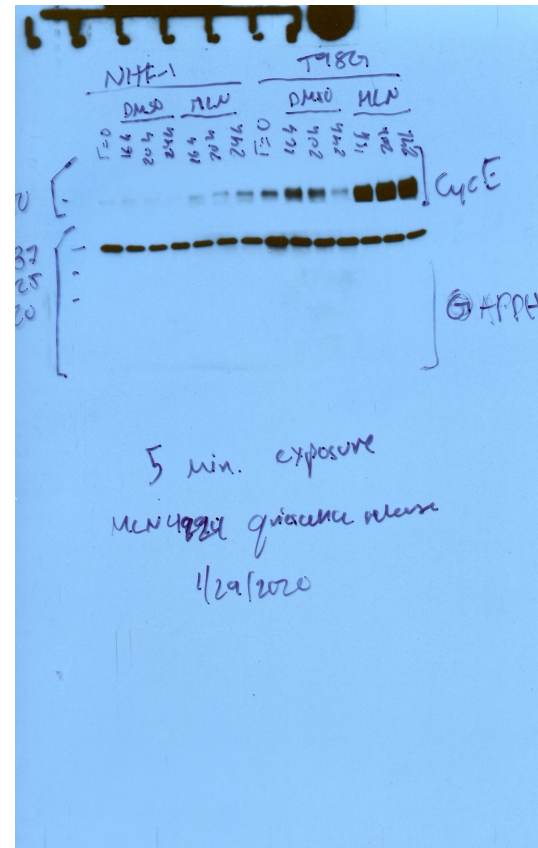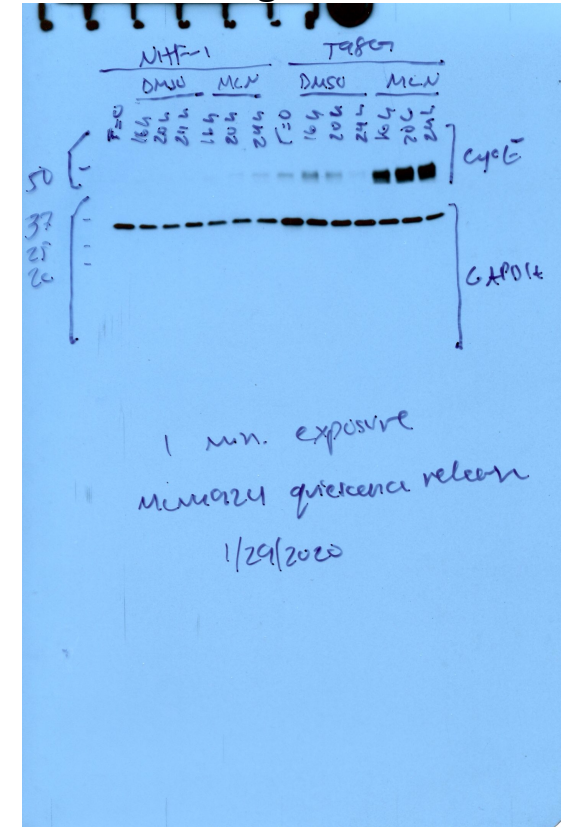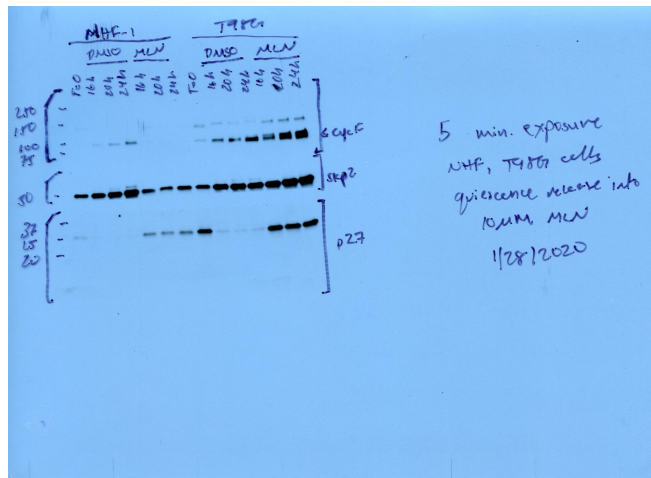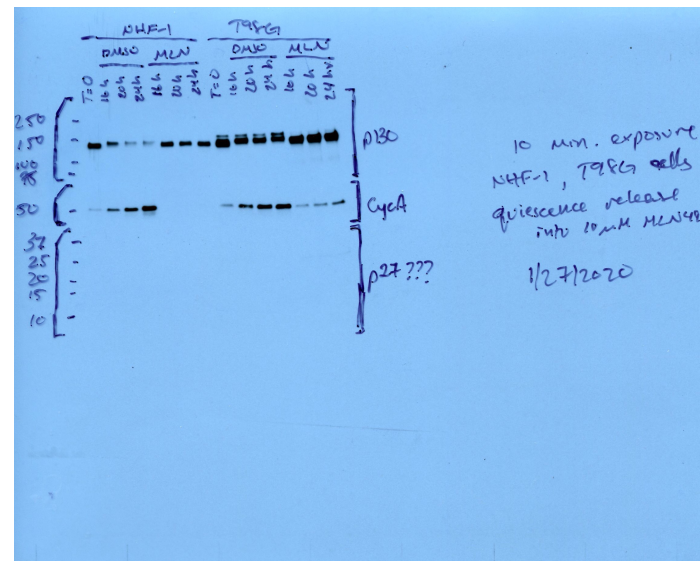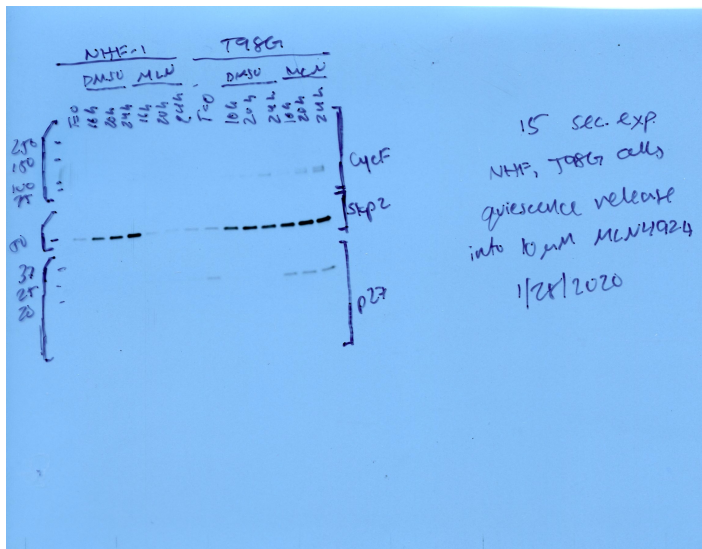

Supplement: Source data 1. — This source data file includes all uncropped blots used to generate data for the main figures and figure supplements. Additionally, copies of the uncropped images are shown a second time where blot strips shown in figures are highlighted with a red square and the protein that was blotted for is noted. [file elife-70691-supp2.zip › Source Data 1/Enrico-Fig1-source-data.pdf]
